# Progressive long‐term spatial memory loss following repeat concussive and subconcussive brain injury in mice, associated with dorsal hippocampal neuron loss, microglial phenotype shift, and vascular abnormalities

**DOI:** 10.1111/ejn.14711

**Published:** 2020-03-12

**Authors:** Marcia G. Honig, Conor C. Dorian, John D. Worthen, Anthony C. Micetich, Isabelle A. Mulder, Katelyn B. Sanchez, William F. Pierce, Nobel A. Del Mar, Anton Reiner

**Affiliations:** ^1^ Department of Anatomy and Neurobiology The University of Tennessee Health Science Center Memphis TN USA; ^2^ Department of Ophthalmology The University of Tennessee Health Science Center Memphis TN USA

**Keywords:** hippocampus, memory, microglia, neurodegeneration, repeat TBI

## Abstract

There is considerable concern about the long‐term deleterious effects of repeat head trauma on cognition, but little is known about underlying mechanisms and pathology. To examine this, we delivered four air blasts to the left side of the mouse cranium, a week apart, with an intensity that causes deficits when delivered singly and considered “concussive,” or an intensity that does not yield significant deficits when delivered singly and considered “subconcussive.” Neither repeat concussive nor subconcussive blast produced spatial memory deficits at 4 months, but both yielded deficits at 14 months, and dorsal hippocampal neuron loss. Hierarchical cluster analysis of dorsal hippocampal microglia across the three groups based on morphology and expression of MHCII, CX3CR1, CD68 and IBA1 revealed five distinct phenotypes. Types 1A and 1B microglia were more common in sham mice, linked to better neuron survival and memory, and appeared mildly activated. By contrast, 2B and 2C microglia were more common in repeat concussive and subconcussive mice, linked to poorer neuron survival and memory, and characterized by low expression levels and attenuated processes, suggesting they were de‐activated and dysfunctional. In addition, endothelial cells in repeat concussive mice exhibited reduced CD31 and eNOS expression, which was correlated with the prevalence of type 2B and 2C microglia. Our findings suggest that both repeat concussive and subconcussive head injury engender progressive pathogenic processes, possibly through sustained effects on microglia that over time lead to increased prevalence of dysfunctional microglia, adversely affecting neurons and blood vessels, and thereby driving neurodegeneration and memory decline.

## Graphical abstract

We found that mice exhibited spatial memory deficits and hippocampal neuron loss a year after repeat weekly head trauma. These adverse outcomes were associated with an increased prevalence of dysfunctional microglia and vascular abnormalities. Our results suggest that repeat brain injury can engender progressive pathogenic processes, through sustained effects on microglia that lead to their dysfunction, which adversely affects neurons and blood vessels, and drives neuronal degeneration and memory loss.

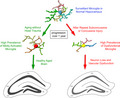

**Abbreviations**
AD: Alzheimer's disease
BBB: blood‐brain barrier
CA: cornu ammonis
CS: conditioned stimulus
CTE: chronic traumatic encephalopathy
CX3CL1: fractalkine
EDTA: ethylenediaminetetraacetic acid
eNOS: endothelial nitric oxide synthase
HCA: hierarchical cluster analysis
IBA1: ionized calcium‐binding adaptor molecule 1
MHCII: major histocompatibility complex II
NeuN: neuronal nuclear antigen
OD: optical density
PAP: peroxidase‐antiperoxidase
PB: sodium phosphate buffer at pH 7.4
PECAM: platelet‐endothelial cell adhesion molecule
PLP: 4% paraformaldehyde, 0.1 M lysine‐0.1 M sodium periodate in 0.1 M PB
PLSD: Protected Least Significant Difference
psi: pounds per square inch
p‐tau: phosphorylated tau
TBI: traumatic brain injury

## 1 INTRODUCTION

About two million Americans annually suffer a traumatic brain injury (TBI) requiring emergency treatment or hospitalization, from sports, recreational activities, vehicular accidents, or military combat, and many more experience TBI but do not seek immediate treatment (Faul, Xu, Wald, & Coronado, 2010; Weiner et al., 2014). TBI can result in a variety of adverse sensory, motor, cognitive and neuropsychiatric deficits, whose nature and severity in the immediate aftermath depend on the characteristics of the initial injury. Importantly, repeat mild‐moderate TBI can also have long‐term adverse consequences that emerge late in life and increase the risk for progressive neurodegenerative diseases, notably Alzheimer's disease (AD), Parkinson's disease, and amyotrophic lateral sclerosis (Washington, Villapol, & Burns, 2016). Much attention has focused on the neurodegenerative syndrome called chronic traumatic encephalopathy (CTE), which is associated with a history of repeated concussive (mild TBI) and/or subconcussive brain trauma (Bailes, Petraglia, Omalu, Nauman, & Talavage, 2013) and defined by the “accumulation of abnormal hyperphosphorylated tau (p‐tau) in neurons and astroglia distributed around small blood vessels” (McKee et al., 2016). However, dementia without the p‐tau pathology characteristic of CTE or without AD‐like β‐amyloid deposition is a far more common outcome (Daneshvar, Goldstein, Kiernan, Stein, & McKee, 2015; Washington et al., 2016). Deficits in episodic, working and spatial memory are among the characteristics of the dementia that often occur in people with a prior history of brain trauma (Esopenko & Levine, 2017; Karr, Areshenkoff, Duggan, & Garcia‐Barrera, 2014; Kasahara et al., 2011; Newsome et al., 2015; Walker & Tesco, 2013).

Mild TBI involves either brief or no loss of consciousness and causes minimal overt brain destruction. However, the compressive, tensile, and shear forces exerted on the brain by a blow to the head, a blast shock wave, or a rapid head acceleration/deceleration (Namjoshi et al., 2013) produce widespread axonal injury that is commonly referred to as “diffuse,” and set in motion a series of secondary degenerative events (Bazarian et al., 2013; Johnson, Stewart, & Smith, 2013; Smith, Hicks, & Povlishock, 2013). Recovery from a single mild TBI is typically clinically complete within weeks (Chadehumbe, 2016; Williams, Puetz, Giza, & Broglio, 2015), and single subconcussive injuries produce no obvious neurological symptoms. The means by which repeated brain trauma, even when widely spaced in time, causes a progressive slow neural decline that can ultimately lead to dementia is uncertain (Ojo, Mouzon, Algamal, et al., 2016; Washington et al., 2016). It is especially unclear why repeated subconcussive trauma is insidious, when each event by itself does not produce any obvious damage (Belanger, Vanderploeg, & McAllister, 2016). The limited understanding of the processes underlying the delayed brain degeneration causing memory impairment has made its progression difficult to detect in humans and to model in animals, resulting in the absence of suitable diagnostic and treatment approaches.

Among the secondary injury cascades following the initial axonal injury produced by mild TBI is microglial activation, which in turn causes further damage and worsens the outcome of the initial trauma (Donat, Scott, Gentleman, & Sastre, 2017; Kumar, Alvarez‐Croda, Stoica, Faden, & Loane, 2016; Kumar & Loane, 2012). Consistent with this, we have found that drugs acting specifically on microglia, biasing them away from the harmful M1 to the helpful M2 state, rescue the functional deficits and neuron loss that would otherwise occur over the first few months following a single mild TBI event (Bu et al., 2016; Guley et al., 2019; Honig et al., 2019; Liu et al., 2017; Reiner et al., 2015). Considerable attention has also focused on the role of microglial alterations in driving delayed neurodegeneration and memory loss (Brown & Vilalta, 2015; Cao, Thomas, Ziebell, Pauly, & Lifshitz, 2012; Loane & Kumar, 2016; Redell & Dash, 2007). A number of investigators have suggested that persistent activation drives the delayed neurodegeneration and memory loss that can occur with a history of repeat TBI (Norden, Muccigrosso, & Godbout, 2015; Witcher, Eiferman, & Godbout, 2015). Paradoxically, other researchers have suggested that a decline in microglia function, resulting from their persistent activation, can render the brain susceptible to delayed degeneration (Biber, Owens, & Boddeke, 2014; Grabert et al., 2016; Khan et al., 2015; Mosher & Wyss‐Coray, 2014; Ojo, Rezaie, Gabbott, & Stewart, 2015; Stojiljkovic et al., 2019; Streit, Xue, Tischer, & Bechmann, 2014).

Here, we sought to examine the long‐term effects of multiple closed‐head brain trauma. Previous studies using a wide range of animal models have generally reported that when spaced more than 2–5 days apart, TBI events are not additive over the short term (Friess et al., 2009; Longhi et al., 2005; Prins, Alexander, Giza, & Hovda, 2013; Selwyn et al., 2016; Vagnozzi et al., 2007; Weil, Gaier, & Karelina, 2014). Because repeat TBI in humans typically occurs outside this window for synergy, in the present study we examined the long‐term impact of TBI events spaced a week apart. As described more fully below, we used a closed‐head focal cranial air‐blast model of mild TBI in mice that produces widespread axonal injury without any contusive brain injury or hemorrhaging (Guley et al., 2016; Heldt et al., 2014). Mice that received a single 50 pounds per square inch (psi) blast using this approach exhibit a variety of functional deficits during the first weeks (up to 2 months), similar to mild TBI in humans, and which we thus refer to here as concussive blast, while 30‐psi blasts are largely asymptomatic, which we thus refer to as subconcussive blast. In the present study, we evaluated the effects of 4 50‐psi or 4 30‐psi blasts spaced a week apart, to model repeat concussive and subconcussive injury, respectively. Given the view that microglia play a key role in the delayed neurodegeneration and memory loss that can occur with a history of repeat TBI, we focused much of our histological analyses on characterizing microglia. We found that both sets of mice exhibited late‐onset spatial memory deficits and hippocampal neuron loss, which were associated with a shift in microglial phenotypes and vascular abnormalities. Surprisingly, repeat subconcussive injury produced greater memory deficits than repeat concussive injury and was accompanied by larger changes in the morphology of microglial processes.

## 2 METHODS

### 2.1 Animals

C57BL/6 male mice were purchased from Jackson Laboratories (Bar Harbor, ME). A single cohort of 60 male mice was subjected to four repeat air blasts at 50‐psi, 30‐psi, or 0‐psi (20 per group), spaced a week apart, starting when the mice were 3 months of age. These mice were tested behaviorally at 4 months and 13–14 months, as described below, and euthanized for histological analysis 15 months after the last blast to determine the long‐term effects of repeat concussive (50‐psi) or subconcussive (30‐psi) blast compared with sham (0‐psi). One mouse from each group died before the 4‐month time point and two mice from each group died between 4 and 13 months. A second set of 3‐month old male mice was subjected to a single blast (12 50‐psi, 14 30‐psi, and 16 sham), and tested behaviorally at 4 and 12 months. A third set of 3‐month old male mice was subjected to a single blast (8 50‐psi, 6 30‐psi, and 6 sham) and euthanized 3 days later for histological analysis to determine if hippocampal microglia show short‐term responses to both concussive and subconcussive blast. All animal studies were performed in accordance with an UTHSC Institutional Animal Care and Use Committee approved protocol (16–133.0 C) and complied with the National Institutes of Health and Society for Neuroscience guidelines. Morphological analyses were performed by individuals who were blinded as to experimental group.

### 2.2 TBI methods

The blast device consists of a modified paintball gun that emits a brief high‐pressure air blast calibrated to the desired pressure. To produce TBI, the air blast is delivered to a 7.5 mm diameter area halfway between the ear and the eye on the left side of the head, encompassing the skull overlying the forebrain (Guley et al., 2016; Heldt et al., 2014). As in most of our prior studies, we used a blast amplitude of 50‐psi above atmospheric pressure to produce a “concussive” injury, which we previously noted yields 98.6% survival in the first few days (Guley et al., 2016). For the current studies, we also used 30‐psi as a “subconcussive” blast level since it is in the range of pressures that produce minimal axonal injury and no significant behavioral deficits at 2–8 weeks after blast (Guley et al., 2016; Heldt et al., 2014). Mice were anesthetized with ketamine/xylazine (90 mg/kg ketamine, 4.5 mg/kg xylazine) injected intraperitoneally (ip) for the repeat TBI studies, or with avertin (400 mg/kg ip) for the single‐injury studies, as described previously (Guley et al., 2016). Anesthetized mice were placed on a cushioned sling, and then inserted into protective tubing that together served to shield and stabilize the mouse. Mice that received a sham (0‐psi) blast were handled in the identical way, including anesthesia and acetaminophen (see below) administration, but with a metal plate inserted between the barrel of the paintball gun and the mouse holder to prevent the air blast from reaching the animal. Mice were subsequently kept warm and typically recovered from anesthesia in 15–30 min. Acetaminophen was added to their drinking water for 2 days, starting the day before the blast was administered, to alleviate any possible pain or discomfort. Based on an average water intake of 4.8 ml/day (https://phenome.jax.org/measureset/40401), we estimate that each mouse received 9.6 mg of acetaminophen a day.

### 2.3 Behavioral studies

#### 2.3.1 Fear acquisition, contextual fear, and conditioned fear tests

Contextual fear spatial memory and cue‐dependent conditioned fear memory following fear conditioning were tested using a Pavlovian fear‐learning paradigm, as described previously (Heldt et al., 2014; Reiner et al., 2015). The fear‐conditioning chamber possesses a stainless steel grid floor (MED Associates, Model ENV‐008) and is fitted with a video camera interfaced with a computer. Automated software (FreezeFrame, Coulbourn) controlled the foot shock and tone stimuli, and collected and analyzed mouse freezing. Mice were allowed to acclimate to the fear‐conditioning chamber for 5 min and then received five fear‐acquisition trials, each consisting of a 30‐s tone (12kH) co‐terminating with a 0.250‐s, 0.4‐mA foot shock, with an intertrial interval of 2 min. Mice were returned to the chamber the following day and given 3 min with no stimulus presentations, sampled over 20 s blocks, to allow assessment of contextual fear to the training chamber. The mice were then presented with tone, but no foot shock, for 15 trials with an intertrial interval of 2 min, to measure retention and extinction of the conditioned fear.

#### 2.3.2 X‐Maze working spatial memory test

As contextual fear testing has an aversive component because of the foot shock used as the unconditioned stimulus, we wanted to also assess performance of the repeat TBI mice in a non‐aversive spatial memory task. We decided to use a delayed spontaneous alternation X‐maze task because prior training is not required, the sensitivity of the test is high (Dudchenko, 2004), and the untimed nature of the assessment makes it relatively unaffected by any possible motor impairment. Spontaneous alternation tasks rely on rodents showing a strong tendency to choose a novel arm over an arm that was previously explored due to their natural exploratory behavior. To choose a novel arm, the animal must retain a memory of previously visited arms (Mohler et al., 2007). Introducing a delay between trials increases the sensitivity of the task (Dudchenko, 2004). For these studies, we used an X‐maze with opaque walls and an eight trial test, with a 120‐s delay after manual return of the mouse after each trial to the start arm. Choosing an arm that the mouse had not selected on either of the two prior choices was considered a “correct” triad choice. Mice were scored for the number of unique triads per test session and the number of hard errors (repeating a choice).

### 2.4 Morphological studies

#### 2.4.1 Tissue fixation and sectioning

Mice were deeply anesthetized (avertin; 400 mg/kg ip), the chest opened, and 0.1 ml of heparinized saline (800 U.S.P. units/ml) injected into the heart. Mice were then perfused transcardially with 30 ml of 0.9% NaCl in 0.1 M sodium phosphate buffer at pH 7.4 (PB), followed by 60 ml of 4% paraformaldehyde, 0.1 M lysine‐0.1 M sodium periodate in 0.1 M PB at pH 7.4 (PLP). Brains were removed, and a pin inserted longitudinally into the right side of each brain, so that the left and right sides of the brain could be distinguished after sectioning by the pinhole. The brain was placed in PLP overnight 4°C to post‐fix, transferred the following day to a 20% sucrose/10% glycerol solution, and stored at 4°C until sectioned. Brains were sectioned frozen on a sliding microtome in the coronal plane at 35 µm, and each brain was collected as 12 separate series in 0.1 M PB with 0.02% sodium azide. Sections from one series were mounted during the sectioning process and stained with cresyl violet. Sections from other series were immunostained as described below.

#### 2.4.2 Immunohistochemical staining

Sections through the hippocampus were selected and processed for single label immunohistochemistry with peroxidase‐antiperoxidase (PAP) staining or for multiple immunofluorescence, as described previously (Guley et al., 2016; Reiner et al., 2015). Sections from some brains and for some antibodies in repeat blast mice required antigen retrieval, which was performed with 1 mM EDTA (ethylenediaminetetraacetic acid), pH 8 at 80°C. Primary antibodies are listed in Table 1. In brief, antibodies detecting the neuronal nuclear antigen (NeuN) antigen, doublecortin, and IBA1 (ionized calcium‐binding adaptor molecule 1) were used in conjunction with PAP immunolabeling to visualize neurons, newly generated immature neurons, and microglia, respectively, and determine their abundance in repeat focal cranial blast mice relative to the matched sham controls. The CP13 monoclonal antibody (kindly provided by Peter Davies, Albert Einstein College of Medicine) was used to detect tau phosphorylated at Ser202. An antibody recognizing IBA1 (guinea pig) was used in combination with antibodies against CD68 (rat), CX3CR1 (rabbit) and/or major histocompatibility complex II (MHCII) (mouse) to characterize microglia by multiple immunofluorescence labeling in repeat focal cranial blast mice and their matched sham controls. Antibodies recognizing CD31/PECAM (rat), collagen IV (rabbit), and endothelial nitric oxide synthase (eNOS) (mouse) were similarly used to examine the vasculature. For multiple immunofluorescence labeling of microglia or the vasculature, the sections were incubated in a mixture of two or three primary antibodies, washed and then incubated with appropriate species‐specific secondary antibodies conjugated to Alexa 488, 555, or 594 (Invitrogen). Primary mouse monoclonal antibodies (i.e., CP13, MHCII, and eNOS) were visualized using subclass‐specific secondary antibodies (goat anti‐mouse IgG1‐HRP for CP13 and goat anti‐mouse IgG1‐AL488, for MHCII and eNOS, both from Thermo Fisher Scientific, Waltham, MA) to minimize non‐specific background staining (Del Mar et al., 2015). Immunohistochemical staining of brain sections from the single focal cranial blast mice and their matched sham controls was limited to double immunofluorescence labeling of IBA1 and CD68.

**Table 1:** Antibodies used in this study

Antibody	Manufacturer, catalog number	Host	RRID#	Dilution
NeuN	Chemicon #MAB377	Mouse monoclonal	177621	1:2,500
NeuN	Abcam #ab177487	Rabbit polyclonal	2532109	1:6,000
doublecortin	Abcam #ab18723	Rabbit polyclonal	732011	1:2,000
IBA1	Wako #019−19741	Rabbit polyclonal	839504	1:2,000
IBA1	Synaptic Systems #234 004	Guinea pig polyclonal	2493179	1:500
CD68	Abcam #ab53444	Rat monoclonal	869007	1:200
CX3CR1	Torrey Pines #TP501	Rabbit polyclonal	10892355	1:1,000, 1:2,000
MHC II	Abcam #ab23990	Mouse monoclonal	447796	1:100
CD31	Novus #NB600−1475	Rat polyclonal	789108	1:100
collagen IV	Biorad # 2150–1470	Rabbit polyclonal	2082660	1:200
eNOS	Transduction Laboratories #N30020	Mouse monoclonal	2314378	1:400
CP13	Dr. Peter Davies	Mouse monoclonal	2314223	1:200, 1:400

Sections prepared for single PAP immunolabeling were mounted onto gelatin‐coated slides, dried, dehydrated, and coverslipped with Permount. Sections prepared for immunofluorescence were mounted onto Superfrost/Plus slides (Fisher), with 2–3 sections through dorsal hippocampus from each of the three experimental groups being mounted onto the same slide. Sections were allowed to dry and then, in the case of sections from mice >1 year old, the slides were briefly immersed in 0.1% Sudan Black in 70% ethanol to quench autofluorescent lipofuscin granules. Slides were washed, stained with DAPI to visualize nuclei, washed again, and then coverslipped in Fluoromount‐G (SouthernBiotech, Birmingham, AL). Image acquisition was specific for the type of analysis and is further described below. All image analysis was performed by individuals blinded as to treatment group.

#### 2.4.3 Volume measurements

The series of sections stained with cresyl violet was used to measure the volume of the dorsal hippocampus in the repeat blast mice and the matched shams. The sections were scanned at 4,800 dpi resolution (Epson Perfection 4490 Photo Scanner), and the hippocampus in those images was outlined in separate layers in Adobe Photoshop and their areas measured. A line from the center of the diencephalon passing through the dorsolateral angle of the thalamus was used to define the border between dorsal and ventral hippocampus. The average area for the 5–7 sections encompassing dorsal hippocampus in the 1 in 12 series was calculated and multiplied by section thickness (35 µm) and the section spacing (35 µm × 12 = 420 µm) to determine volume.

#### 2.4.4 Neuron counts

A one‐in‐twelve series of coronal sections in the repeat blast mice and the matched sham mice was immunolabeled for NeuN. Slides were scanned using an Aperio ScanScope XT scanner (Aperio Technologies). A standard level of dorsal hippocampus, centered on Bregma −2.0, was used for blinded neuron counts for dentate gyrus and cornu ammonis 1 (CA1). For dentate gyrus, 10 evenly spaced counting boxes, each 40 µm in the medial‐lateral axis and spanning the entire dorsal‐ventral width of the upper leaflet, were overlain on the image in a separate Photoshop layer. The most medial box was not used for neuron counts since the upper and lower leaflets sometimes overlapped in this location. The lateral boundary of CA1 was drawn as shown in the Allen Mouse Brain Atlas, based on the expression of markers specific for CA1 (epithelial membrane protein 1) versus CA2 (membrane protein, palmitoylated 3), and a series of 10 evenly spaced counting boxes were similarly applied to the image. For each counting box, neurons falling on the top and left boundaries were included in the counts, but neurons on the bottom and right boundaries were not. The average number of neurons per box was calculated, and the area of each counting box occupied by neurons and the total area of the upper leaflet of dentate or CA1 were measured using Photoshop tools. These values were then used to calculate overall neuron density and total neuron number per length of dentate or CA1.

#### 2.4.5 IBA1 counts

A one‐in‐twelve series of coronal sections in repeat blast mice and the matched shams was immunolabeled for IBA1, and slides were scanned using an Aperio ScanScope XT digital slide scanner. Microglia were counted in sections at the same standard level of dorsal hippocampus as above, by marking IBA1+ cellular profiles in an overlying layer in Photoshop. The hippocampus in the selected section was outlined; its area was measured and used to calculate microglial density.

#### 2.4.6 Doublecortin counts

A one‐in‐twelve series of coronal sections was immunolabeled for doublecortin, and images of the dentate gyrus were captured and doublecortin+ neurons counted throughout the rostrocaudal extent of dorsal hippocampus in repeat blast mice and the matched shams using a 40x objective on an Olympus BH2 series light microscope with S Plan Apochromat objectives, an achromatic condenser (Olympus Corporation) and SPOT idea camera (Diagnostic instruments, Inc.) running on SPOT Advanced software (Version 4.6). Doublecortin+ cells with obvious cell bodies were counted, regardless of the presence of stained dendritic processes, through the entirety of dorsal hippocampus, using the same boundary between dorsal and ventral hippocampus described above for volume measurements.

#### 2.4.7 Capture of immunofluorescent labeling

Sections through dorsal hippocampus that had been prepared for immunofluorescence were viewed with a Zeiss 710 confocal microscope (Carl Zeiss AG) using a 20x, 0.8 numerical aperture objective. For each side of the brain, images of a 1,234‐µm by 830‐µm area covering most of the central portion of stratum radiatum of CA1 through stratum moleculare of the dentate were acquired using the tile capture function of the Zen software (Zen Black Version 2.1, Carl Zeiss AG). As our analysis of microglia and the vasculature included optical density measurements to determine the relative expression of different markers, it was essential to standardize the capture conditions for each marker across sets of images. To accomplish this, laser power and gain were adjusted to optimize image quality for each marker (for the sections on a given slide) and images from each of the 3 experimental groups that had been mounted on the same slide were acquired under identical conditions in the same capturing session. A short series of z‐stacks (3 for analysis of microglia, 4 for the vasculature) were acquired at 2‐µm intervals, using a pinhole setting of 2 Airy units, to expedite the capturing process. The individual z‐stack images were used to generate maximum intensity projection images with the Zen software, and exported as tiff files for analysis with Adobe Photoshop and FIJI (FIJI is Just ImageJ; https://imagej.net/ImageJ). For subsequent morphometric analysis of microglia in repeat blast mice and the matched shams, the IBA1 immunolabeling was then captured from a smaller area (415‐µm by 830‐µm) within the same field of view at a higher resolution, a pinhole setting of 1 Airy unit, and 0.5 µm intervals between the individual z‐stacks.

#### 2.4.8 Analysis of microglial marker expression

To assess microglial expression, we combined IBA1 immunolabeling with immunolabeling for CD68 for short‐term single blast mice, long‐term repeat blast mice, and their respective sham groups, plus either MHCII or CX3CR1 for the repeat blast mice and their matched shams. To analyze the multiple labeling, the IBA1 signal was used to create a mask of individual microglial cells that was subsequently directed to the various channels to measure the optical density of each channel for each cell. The approach involved the following steps. 1) The DAPI image was used to determine the boundaries of the area of interest, specifically from the ventral border of the pyramidal cell layer of CA1 to the dorsal border of the granule layer of the upper leaflet of the dentate gyrus, outlined in a separate layer of Photoshop and the rest cropped so that microglia within the densely packed neuronal layers and within the corpus callosum would be excluded from the analysis. 2) The optical density (OD) for each channel was measured in 3–5 small areas within stratum lacunosum/moleculare to determine the level of background staining for that image, and the average of those ODs used to adjust that channel to a background optical density of 50 (1 = black, 255 = white) using FIJI. 3) The background‐subtracted IBA1 image was thresholded by eye to select microglia in a consistent manner across samples. The thresholded image was then imported as a separate layer onto the original image, and drawing tools in Photoshop were used to remove extraneous processes and perivascular microglia to produce a mask. 4) The mask and the background‐subtracted images for the individual channels were opened in FIJI, and the analyze particles function was directed to each channel to obtain the OD for each marker for each cell. Data from individual cells were imported into Excel. 5) To normalize the data from all the mice analyzed this way, the ODs for the microglia from the three mice with sections on the same slide were combined into a single Excel worksheet. The ODs for each channel were rank ordered, separately for each side of the brain, and then expressed on a 0–1 scale, with 0.01 indicating the lowest expression level and 1.00 the highest expression. Thousands of microglia were analyzed per group per side using this approach for repeat blast mice and the matched sham mice, and hundreds were analyzed in single blast mice and their matched shams.

#### 2.4.9 Analysis of microglial morphology

The high‐resolution images of IBA1 immunofluorescence for repeat blast and sham mice were imported as lsm or czi files into Neurolucida 360 (MicroBrightfield Bioscience). A minimum of 10 IBA1+ cells was chosen from each image of dorsal hippocampus between the granule cell layer of the dentate and the pyramidal cell layer of CA1 in an unbiased manner by an individual blinded to group membership and without regard to microglia features, albeit selecting from areas of low‐medium cell density to minimize overlap with the processes of neighboring microglia and avoiding microglia closely associated with vascular elements. Cell bodies and processes were traced, and numerous morphometric features were then analyzed with the software. The most useful parameters provided by the Neurolucida software and for which data are presented are as follows: 1) soma volume; 2) soma aspect ratio calculated by dividing the diameter of the major axis of the soma by the diameter of the minor axis, at the level of its largest perimeter, with cells having aspect ratios greater than 1.0 being ovoid (i.e., flattened), and cells having an aspect ratio of 1.0 being round; 3) soma form factor calculated using the formula [4π * area]/[perimeter]^2^ (A circle has a form factor of 1.0, and as the perimeter becomes more jagged, the form factor approaches 0); 4) total process length; 5) process complexity calculated using the formula [total number of branches + number of endings] * [total process length/ number of primary processes]; 6) process convex hull volume, which is the volume of the polygon generated by connecting the tips of all the processes, and serves as a measure of overall coverage. From measurements provided by the Neurolucida software, we calculated additional parameters: 7) process branchiness calculated by dividing the number of branch points by the number of primary processes; 8) the density of coverage within the territory covered by the cell (i.e., its convex hull), calculated by dividing the volume of the processes by the convex hull volume; 9) process distribution uniformity; and 10) process distribution circularity. For the latter two, the wedge analysis feature of the Neurolucida software was used to divide the area of interest centered on the cell body into eight wedges and determine the length of processes in each wedge, which was then expressed as a percentage of the total process length for that cell. The first parameter, the process distribution uniformity score, reflects the degree to which processes are uniformly distributed around the cell body and was calculated by measuring the extent to which each octant around the cell body deviates from possessing 1/8 of the processes. Perfect uniformity would be represented by a score of 1. If the processes occupied 4 of the 8 octants, the uniformity score would be 0, and if all the processes were limited to a single octant, the uniformity score would be −0.5. To calculate the process distribution circularity score, the percentages for processes in opposing quadrants were summed, and the two resulting sums divided into one another to derive a minimum/maximum ratio. For microglia with an equal abundance of processes in each quadrant, that is, a circular distribution, the score would equal 1, whereas for cells with processes disproportionately more abundant along the long axis than the short, that is, a flatter distribution, the score would approach 0. Note that for cells having intermediate uniformity scores, microglial processes could be arranged either circularly or in a more linear manner. Low values of either process distribution uniformity or process distribution circularity would suggest diminished surveillance of the territory the processes cover.

#### 2.4.10 Analysis of the vasculature

To assess the vasculature in repeat blast and sham mice, we used immunofluorescent labeling and antibodies recognizing CD31/PECAM, eNOS, and collagen IV. The approach for the analysis was modified from that described above for microglial expression and involved the following steps. 1) Hippocampal boundaries were drawn in a separate layer of Photoshop from the ventral border of pyramidal cell layer of CA1 to the dorsal border of the granule layer of the upper leaflet of the dentate gyrus, and the outlying area cropped and excluded from further analysis. The borders between strata were traced so that stratum moleculare of the dentate gyrus, stratum radiatum of CA1, and stratum lacunosum/moleculare (which contains afferent fibers from the entorhinal cortex and the arterial supply entering along the hippocampal sulcus; henceforth simply referred to as the sulcus) could each be analyzed separately. 2) The CD31 immunolabeling was used to create a mask using a combination of thresholding and drawing tools. The CD31 mask was imported into FIJI, and the length of capillaries in stratum moleculare of dentate gyrus and in stratum radiatum of CA1 was measured. In the case of the sulcus, only those arterioles that were cut transversely were analyzed; vessels sectioned longitudinally or obliquely were excluded. 3) The OD for each channel was measured in the sulcus to determine the level of background staining and used to adjust each channel to a background optical density of 50 (1 = black, 255 = white) using FIJI. 4) The CD31 mask and the background‐subtracted images for the individual channels were opened in FIJI, and the mask was directed to each channel to obtain the OD for each marker.

### 2.5 Statistics

One‐way ANOVA followed by planned comparisons using post hoc Fisher PLSD tests (Protected Least Significant Difference) was used to analyze fear behavior and histological data. Paired *t* tests were used to evaluate X‐maze performance for the repeat 50‐psi, the repeat 30‐psi, and the sham mice, separately, relative to chance performance (Paterno, Metheny, & Cohen, 2018). In the case of neuron counts, microglial abundance, and the vascular analysis, values from the left and right sides of the sham mice were pooled for statistical analysis, and the averages were used as the control values. Similarly, for analysis of expression and morphometry of individual microglia, the values from cells on the left and right sides of the sham mice were pooled for statistical analysis and the averages used as the control values.

### 2.6 Hierarchical cluster analysis

As we had collected expression data, cell body shape data, and process shape data for a minimum of ten microglia from each of six cases from each experimental group among the repeat blast and sham mice, we used hierarchical cluster analysis (HCA) to profile microglia across all groups to determine if the microglia partitioned into distinct clusters. Our goal was to determine how the relative proportions of the clusters differed between the repeat concussive mice, the repeat subconcussive mice, and the sham mice, and to determine if some clusters were linked to memory loss and hippocampal pathology after repeat blast. For this analysis, we pooled data from 429 microglia across the two sides of hippocampus and across experimental groups for the following 14 traits: 1) IBA1 expression; 2) CD68 expression; 3) MHCII expression; 4) CX3CR1 expression; 5) soma volume; 6) soma aspect ratio; 7) soma form factor; 8) total process length; 9) process branchiness (number of nodes/number of processes); 10) process complexity; 11) convex hull volume; 12) density of coverage; 13) process distribution uniformity; and 14) process distribution circularity. The traits chosen (or variations thereof) had been found to aid in defining microglia subtypes in prior HCA studies (Fernández‐Arjona, Grondona, Granados‐Durán, Fernández‐Llebrez, & López‐Ávalos, 2017; Verdonk et al., 2016; Yamada & Jinno, 2013) and/or had a multimodality index suitable for HCA (i.e., above 0.55). Missing values were imputed using the multiple imputation by chained equations method (Azur, Stuart, Frangakis, & Leaf, 2011; Van Buuren & Groothuis‐Oudshoorn, 2011). HCA was performed on z‐transformed data sets based on Ward's method using Euclidean distances as a measure of similarity, using the software program R. The Average Silhouette Method was used to determine the optimal number of clusters.

## 3 RESULTS

In these studies, we examined behavioral deficits after repeat 50‐psi focal cranial air blast (i.e., concussive) and repeat 30‐psi focal cranial air blast (i.e., subconcussive) at 4 months and at 13–14 months after the last blast, compared to sham mice. We found impaired spatial memory in mice subjected to both levels of repeat injury at the later time point, but not earlier, suggesting a progressive decline. Given the established role of the dorsal hippocampus in cognitive processes, particularly spatial learning (Fanselow & Dong, 2010), we focused the histological analysis we performed at 15 months on the dorsal hippocampus to determine if neuronal loss was associated with these deficits and to characterize any underlying microglial and vascular alterations. We also examined the effects of a single injury for a few endpoints relevant to the effects of repeat injury. Before discussing the cognitive deficits and associated hippocampal pathologies found with repeat injury, below we briefly describe some key features of our model.

### 3.1 Overview of Focal Cranial Blast Model and the Effects of Single Blasts

**Figure 1:**
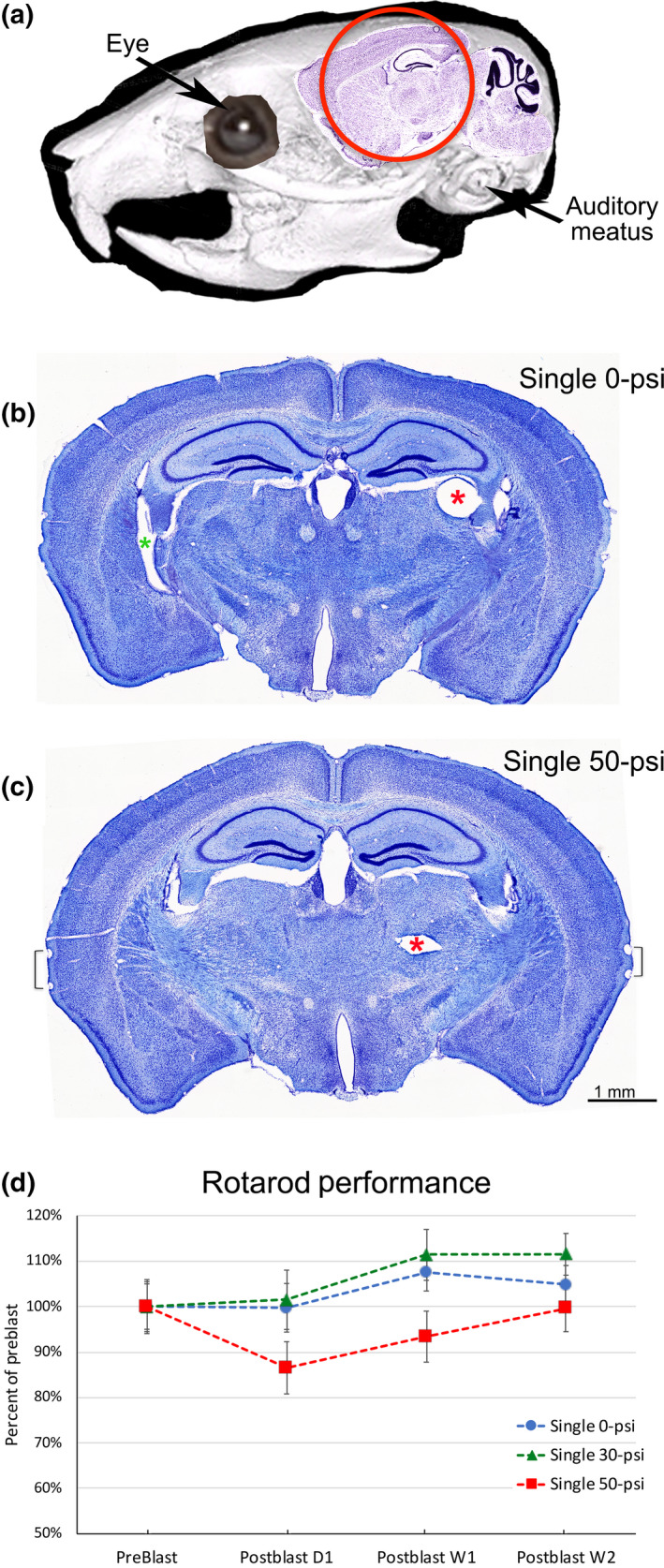
Focal cranial air‐blast model and effects of single blast. (a) Superimposition of images of a Nissl‐stained sagittal section (Allen Mouse Brain Atlas) and a mouse head to show the 7.5 mm area (red circle) targeted by the air blast (modified from Guley et al., 2016). Note that the dorsal hippocampus is positioned roughly in the center of the blast area. (b, c) Cresyl violet‐stained coronal sections from a sham mouse (b) and a mouse that had received a 50‐psi blast (c), 3 days prior. Note the absence of any overt brain damage, particularly contusive injury on the left side of the cerebral cortex, that is, that closest to the blast. The sections shown are at the standard level of the brain we used to analyze the dorsal hippocampus, Bregma −2.06 for b and Bregma −1.94 for c. The red asterisks in b and c mark holes left by pins inserted longitudinally into the right side of the brain before it was sectioned so that the two sides could later be distinguished. The green asterisk in b marks the left lateral ventricle, which appears larger than that on the right side due to the slight asymmetry with which the brain was sectioned. The black brackets in c mark blood vessels on the surface of the cerebral cortex; these are a normal feature as additional blood vessels are visible along the brain surface in b as well as in c. The scale bar in c applies to both images. (d) Rotarod performance of mice that received a single blast at 50, 30, or 0‐psi. Mice were trained on an accelerating rotarod 2 days before blast, and began testing the following day, each test consisting of two trials. The average latency to fall was determined for each mouse, the average calculated for each group, and that average normalized to the group's latency the day before blast. Mice were assigned to experimental groups so that the average time to fall would be similar between groups. Data are taken from mice tested in Guley et al., 2016 (Figure 6a). The single 30‐psi blast mice (*n* = 18) and the sham mice (*n* = 24) performed similarly across trials at 1 day, 1 week, and 2 weeks after blast. The 50‐psi blast mice (*n* = 16) showed a small but significant deficit compared with sham at 1 day and 1 week (*p* = .0121), but nearly full recovery by 2 weeks. Error bars are SEMs

We developed our focal cranial air‐blast model several years ago to produce mild TBI in mice using a closed‐head approach that does not involve the animal's body or damage to the skull and is characterized by widespread axonal injury (Guley et al., 2016; Heldt et al., 2014). The goal was to create an injury that would be analogous to concussion in humans in terms of functional deficits and pathology. The air blast is delivered to a small area on the left side of the head midway between the ear and the eye. The superimposition of images of a Nissl‐stained sagittal section and the mouse head shows that the dorsal hippocampus is positioned roughly in the center of the area targeted by the blast (Figure 1a). “Concussive” 50‐psi air blasts do not produce contusive injury in the region of cortex that the blast wave reaches first or any other overt brain damage (Figure 1b,c). As part of characterizing the dynamics of the blast forces and the associated functional deficits and pathologies, we had previously assessed motor function in the short‐term aftermath of the injury, comparing the results for mice that had received a single air blast in the 0–20 psi range, the 25–40 psi range, and the 50–60 psi range. The 50–60 psi mice exhibited significant motor deficits, whereas the 25–40 psi mice exhibited minimal or no deficits, depending on the parameter being reported (Guley et al., 2016). To address the issue of whether a single 30‐psi blast has a deleterious outcome, here we present the rotarod data for mice that had received a single blast at 50‐psi, at 30‐psi, or at 0‐psi (sham). As shown in Figure 1d, the single 30‐psi blast mice performed similarly to the sham mice across trials at 1 day, 1 week, and 2 weeks after blast, whereas single 50‐psi blast mice showed a small deficit in rotarod performance compared with sham at 1 day and 1 week (*p* = .0121), but noteworthy recovery by 2 weeks. The small, transient functional deficits exhibited by the single 50‐psi blast mice are consistent with studies of mild TBI produced by impact (Hylin et al., 2013; Longhi et al., 2005; Mouzon et al., 2012) and with the general consensus that, for the majority of concussed adults, symptoms largely resolve in 7–10 days (Blennow et al., 2016; McCrory et al., 2013). Our previous histological analyses revealed widespread (i.e., diffuse) axonal injury in white matter tracts in the form of swollen axonal bulbs during the first few days after a single 50‐psi blast (Guley et al., 2016, 2019; Honig et al., 2019), and the later degeneration of some of the injured axons.

### 3.2 Functional deficits

#### 3.2.1 Fear acquisition, contextual fear and conditioned fear results following repeated focal cranial air blast

**Figure 2:**
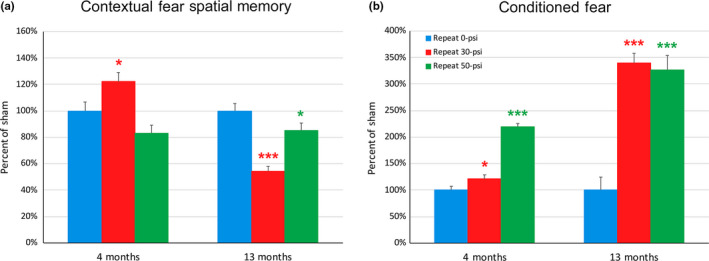
Contextual fear (a) and conditioned fear (b) after repeat focal cranial air blast. Repeat subconcussive mice showed a slight but significant elevation in contextual fear at 4 months but significant loss at 13 months, indicating a late‐onset spatial memory deficit. Their conditioned fear retention was, by contrast, enhanced at 13 months. Repeat concussive mice showed slightly reduced contextual fear that was significantly less than that for the matched sham mice at 13 months but not at 4 months. Conditioned fear responses were, however, elevated above sham at both time points. *n* = 19 mice/group at 4 months, 17 mice/group at 13 months. Error bars are SEMs. Statistics are by one‐way ANOVA across the 9 contextual fear trials or the 15 conditioned fear trials. Red asterisks indicate significant differences between the repeat subconcussive mice and the matched sham mice; green asterisks indicate significant differences between the repeat concussive mice and sham, *p < .05, ***p < .001

At 4 months after the last blast, the repeat 50‐psi and the repeat 30‐psi blast mice showed fear responses during the acquisition session that were similarly enhanced compared to the matched sham mice, with fear responses over the last three trials significantly greater than in the sham mice (*p* = .022 for repeat 30‐psi; *p* = .019 for repeat 50‐psi). The day after fear acquisition, the repeat 30‐psi mice exhibited significant increases both in contextual fear and in conditioned fear (Figure 2). By contrast, the repeat 50‐psi mice exhibited contextual fear over the nine test blocks that trended toward being less than in the shams (*p* = .069), but their conditioned fear retention over the 15 presentations of the conditioned stimulus (CS) was significantly heightened, compared with both the sham and the repeat 30‐psi blast mice. The results at 13 months after blast were very different than at 4 months. At 13 months, the repeat 50‐psi blast mice showed significantly increased responses over the last three trials of fear acquisition compared with sham mice (*p* = .006), while the repeat 30‐psi blast mice did not differ from sham. Most strikingly, and in contrast to the results at 4 months, the repeat 30‐psi blast mice exhibited a large deficit in contextual fear (to about 50% of sham) the day after fear acquisition and the repeat 50‐psi blast mice exhibited a small (to about 85% of sham), but now significant, deficit. Additionally, the two groups of repeat TBI mice exhibited large increases in conditioned fear at 13 months, compared to sham. The reduction in contextual fear, despite the increased fear‐learning and/or fear‐extinction responses, indicates a loss of spatial memory for both the repeat 30‐psi and the repeat 50‐psi blast mice at 13 months after blast.

#### 3.2.2 Fear acquisition, contextual fear, and conditioned fear following single focal cranial air blast

**Figure 3:**
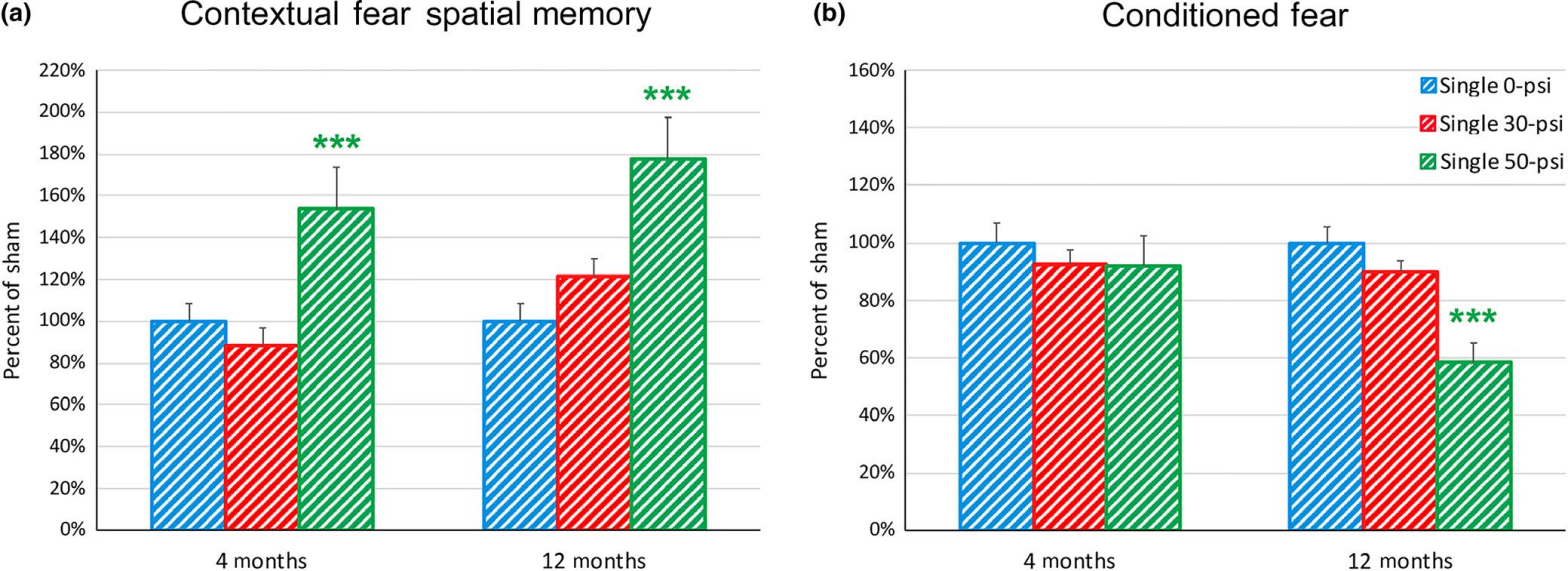
Contextual fear (a) and conditioned fear (b) after single focal cranial air blast. In contrast to the repeat TBI mice, neither the single 30‐psi nor the single 50‐psi mice exhibited contextual fear deficits 4 or 12 months after a single blast. Contextual fear for single‐blast 50‐psi mice was greater than that for the matched sham mice at both time points, while contextual fear for single‐blast 30‐psi mice was not significantly different than that for sham mice at either time point. Conditioned fear responses were similar for all 3 groups of mice at 4 months, whereas single 50‐psi mice, but not single 30‐psi mice, exhibited less conditioned fear than sham mice at 12 months. For both time points, *n* = 14 30‐psi mice, 12 50‐psi mice, and 16 sham mice. Error bars are SEMs. Statistics are by one‐way ANOVA across the 9 contextual fear trials or the 15 conditioned fear trials. Green asterisks indicate significant differences between single 50‐psi mice and sham mice, ***p < .001

To determine if the focal cranial air blast must be repeated to produce a long‐term decline in spatial memory, we evaluated contextual fear at 4 and 12 months after a single blast of 50‐psi, 30‐psi, or 0‐psi. At 4 months, the three groups of mice showed similar fear responses during the acquisition session, with the neither the 50‐psi nor 30‐psi mice differing significantly from sham across the last three acquisition trials (*p* = .8581 for 50‐psi; *p* = .1206 for 30‐psi). The day after fear acquisition, contextual fear was significantly greater across all trials in the single‐blast 50‐psi mice (*p* = .000023), but not in the single‐blast 30‐psi mice, as compared to sham mice, yet the three groups of mice exhibited similar levels of conditioned fear across the 15 CS trials (Figure 3). When fear was assessed at 12 months, the three groups of mice again showed similar fear acquisition, with no significant difference between the sham and concussive mice across the last three trials, and a small reduction in subconcussive compared to sham mice (*p* = .0347). However, the following day, the single 50‐psi mice showed significantly greater contextual fear across all trials than the sham mice (*p* = .00000001) and contextual fear for the single 30‐psi mice trended toward an increase (*p* = .0682). Conditioned fear responses for the single 50‐psi mice at 12 months showed different results than at 4 months, with them now exhibiting significantly less conditioned fear than the sham mice (*p* = .000001), but conditioned fear for the single 30‐psi mice still similar to that for sham mice. Thus, in contrast to the repeat TBI mice, neither the single 30‐psi nor the single 50‐psi mice exhibited contextual fear deficits at either 4 or 12 months. Accordingly, for the remainder of the studies reported here, we focus on the repeat TBI mice, unless otherwise noted.

#### 3.2.3 X‐Maze spatial working memory following repeated focal cranial air blast

We next used an X‐maze task to examine spatial memory in the repeat TBI mice and the sham controls. As rodents typically choose one of the novel arms over the previously explored arm when they are allowed to wander in an X‐maze, normal spatial memory is indicated by a level of performance greater than would occur simply by chance. The sham mice performed significantly better than chance, based on both the number of uniquely alternated triads and the number of hard errors (i.e., repeat arm entry on successive trials) in tests conducted 14 months after blast. The repeat 30‐psi mice, however, performed only at chance levels using both criteria, whereas the repeat 50‐psi mice showed a lesser impairment, since they were better than chance in terms of hard errors but at chance in terms of correct triads (Table 2). Thus, the repeat 30‐psi focal cranial air‐blast mice demonstrated more impairment in the spontaneous alternation working memory task than did the repeat 50‐psi focal cranial air‐blast mice, similar to the results for contextual fear spatial memory.

**Table 2:** X‐Maze results

Experimental group	Correct triads	Hard errors	Correct triads and hard errors
Repeat sham	**11.2%** (*p* = .031)	**14.8%** (*p* = .0005)	**26.0%** (*p* = .002)
Repeat 30‐psi	1.4% (*p* = .630)	6.4% (*p* = .168)	7.8% (*p* = .391)
Repeat 50‐psi	3.4% (*p* = .563)	**10.6%** (*p* = .015)	14.0% (*p* = .117)

Note

The number of correct triads (three consecutive unique arm choices) and the number of hard errors (repeating the same arm choice on a consecutive trial) on X‐maze were determined for mice with repeat focal cranial blast. A mouse performing randomly, that is, lacking any spatial memory, would make hard errors one‐third of the time and correct triads 22% of the time. In the Table, performance is presented as the percent better than chance, and performance significantly better than chance is shown bolded. The matched sham mice performed significantly better than chance (more correct triads, fewer hard errors), indicating that their spatial memory was intact. By contrast, the repeat 30‐psi mice performed at chance levels by both standards, indicating that their spatial memory was impaired. The repeat 50‐psi mice showed some, albeit less impairment, in that they performed significantly better than chance on hard errors, but at chance in correct triads.

### 3.3 Neuropathology following repeated focal cranial air blast

#### 3.3.1 Dentate gyrus

**Figure 4:**
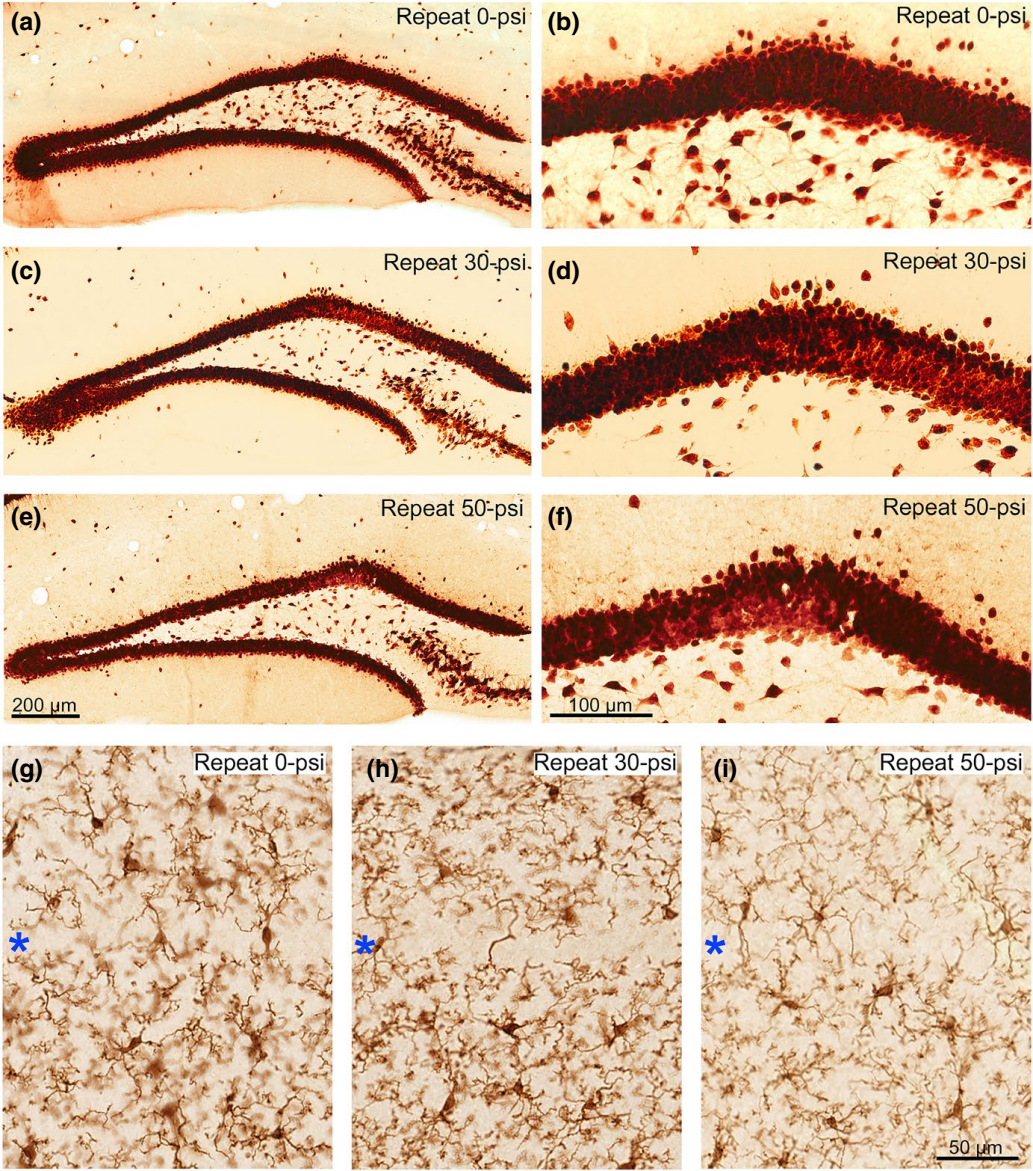
Loss of dentate gyrus neurons and microglia in dorsal hippocampus at 15 months after repeat focal cranial air blast. (a–f) Images show NeuN immunolabeling of the granule cell layer of the right dentate gyrus at a low magnification in the panels on the left and a higher magnification in the panels on the right. The localized loss of NeuN+ granule cells can be seen in the higher magnification views of the upper leaflet of the dentate in the repeat TBI mice. (g–i) Images show IBA1+ microglia in CA1 of the right dorsal hippocampus. Microglia are less abundant in the repeat subconcussive mice than in the sham and repeat concussive mice. Also note that the IBA1 immunolabeling is more intense in the repeat subconcussive mice than in the sham mice and less intense in the repeat concussive mice than in the sham mice. Blue asterisks in g–i mark the pyramidal cell layer of CA1. Scale bar in e applies to a, c, and e; scale bar in f applies to b, d, and f; scale bar in i applies to g–i

**Figure 5:**
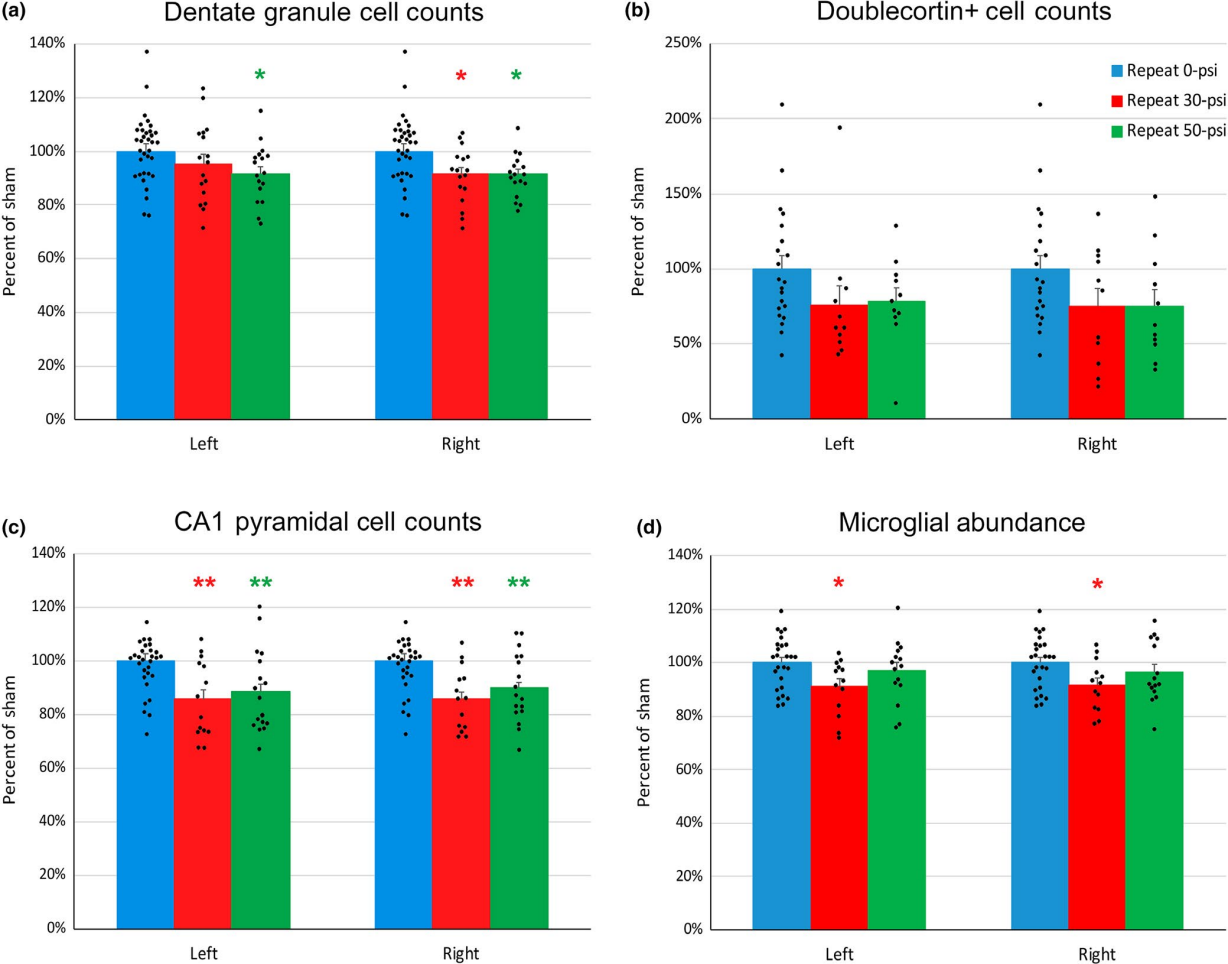
Reduced abundance of neurons and microglia in dorsal hippocampus at 15 months after repeat focal cranial air blast. (a) Repeat concussive mice show significant neuron loss in the upper leaflet of the dentate gyrus on both sides and repeat subconcussive only on the right. (b) The abundance of doublecortin+ neurons in dentate gyrus was decreased on both sides of hippocampus in both repeat concussive and subconcussive mice, with the losses trending toward, but not reaching, statistical significance (*p* = .078–.126). (c) Repeat subconcussive and repeat concussive mice both show significant neuron loss on both sides of CA1. (d) The density of IBA1+ microglia in dorsal hippocampus was significantly reduced on both sides in repeat subconcussive mice, but not different than sham for repeat concussive mice. The left and right sides for the matched sham mice were pooled for statistical analysis here and all other statistical comparisons. The number of mice per group per side that were analyzed was 17 for dentate, 11 for doublecortin, 14–17 for CA1, 13–15 for microglia. Error bars are SEMs. Red asterisks indicate significant differences between the repeat subconcussive mice and the matched sham mice; green asterisks indicate significant differences between the repeat concussive mice and the matched sham mice, *p < .05, **p < .01

We focused our histological analyses on the dorsal hippocampus and started by examining sections immunolabeled for the pan‐neuronal marker NeuN in the repeat TBI mice and the sham controls. We noted a thinning of the dentate in some of the repeat focal cranial air‐blast mice and/or instances of patches in the upper leaflet from which dentate granule cells appeared to be absent (Figure 4a‐f). Adjacent cresyl violet‐stained sections showed shrunken, pyknotic neurons occupying these patches, thus suggesting the presence of dying neurons that no longer expressed NeuN. We then performed blinded analysis at a standard level of dorsal hippocampus, counting from 40‐µm long sampling boxes placed at uniform intervals along the medial‐lateral axis of the upper leaflet, and expressed the data as neuron abundance per 100‐µm length of the dentate. The results showed an 8.6% loss of dentate granule cells on the contrecoup right side for both the repeat 50‐psi mice and the repeat 30‐psi mice. On the left side, we observed a similar loss of dentate granule cells for the repeat 50‐psi mice and a smaller, insignificant reduction for the repeat 30‐psi mice (Figure 5a).

Dentate granule cells are generated throughout the life span from precursor cells in the underlying subgranular zone, and newly generated immature neurons can be recognized by doublecortin immunolabeling (Bregy et al., 2012; Brown et al., 2003; Kunimoto, Nakamura, Wada, & Inoue, 2010). The number of doublecortin+ neurons throughout the rostrocaudal extent of the dorsal hippocampus was decreased by nearly 25% on the right side for both the repeat 30‐psi and the repeat 50‐psi mice, and by ~ 22% on the left (Figure 5b). Although the decline in doublecortin+ neurons trended toward but did not achieve statistical significance (*p* = .078–.126), we found a significant correlation between the numbers of doublecortin+ neurons and dentate granule cells (*r* = .3355; *p* = .0063) across the three experimental groups and the two sides of hippocampus. It thus seems likely that the production and/or survival of new neurons was diminished as a consequence of repeat focal cranial air‐blast injury and this decrease, over time, contributed to the reduced abundance of dentate neurons.

#### 3.3.2 CA1

We counted neurons in CA1 at a standard level of dorsal hippocampus, expressing the data as neuron abundance per 100‐µm length to compare between experimental groups. The results showed ~14% bilateral loss of CA1 pyramidal neurons for the repeat 30‐psi mice and 10%–11% bilateral CA1 neuron loss for the repeat 50‐psi mice (Figure 5c). Thus, neurons in CA1 were lost to fairly similar extents on both sides of the brain at the two blast levels.

### 3.4 Microglial abundance, expression, and morphology following repeated focal cranial air blast

#### 3.4.1 Microglial abundance

We next evaluated microglia, because of their important roles in healthy, injured, and diseased brains. To assess microglial abundance, we examined a standard level through dorsal hippocampus (as for neuron counts) and counted cells immunolabeled for IBA1. The results showed an 8%–9% reduction in microglial density on both sides of dorsal hippocampus for the repeat 30‐psi mice, but no significant change for the repeat 50‐psi mice (Figures 4g–i and 5d). As dorsal hippocampal volume was not significantly different between repeat blast and sham mice, the reduced density of microglia in the repeat 30‐psi mice presumably stems from their loss. Interestingly, we found microglial abundance was significantly correlated with neuron abundance in CA1 (*r* = .2551; *p* = .0224) across the three experimental groups and the two sides of hippocampus, suggesting an adverse effect of microglial loss on neuronal survival, at least for CA1.

#### 3.4.2 Microglial expression

**Figure 6:**
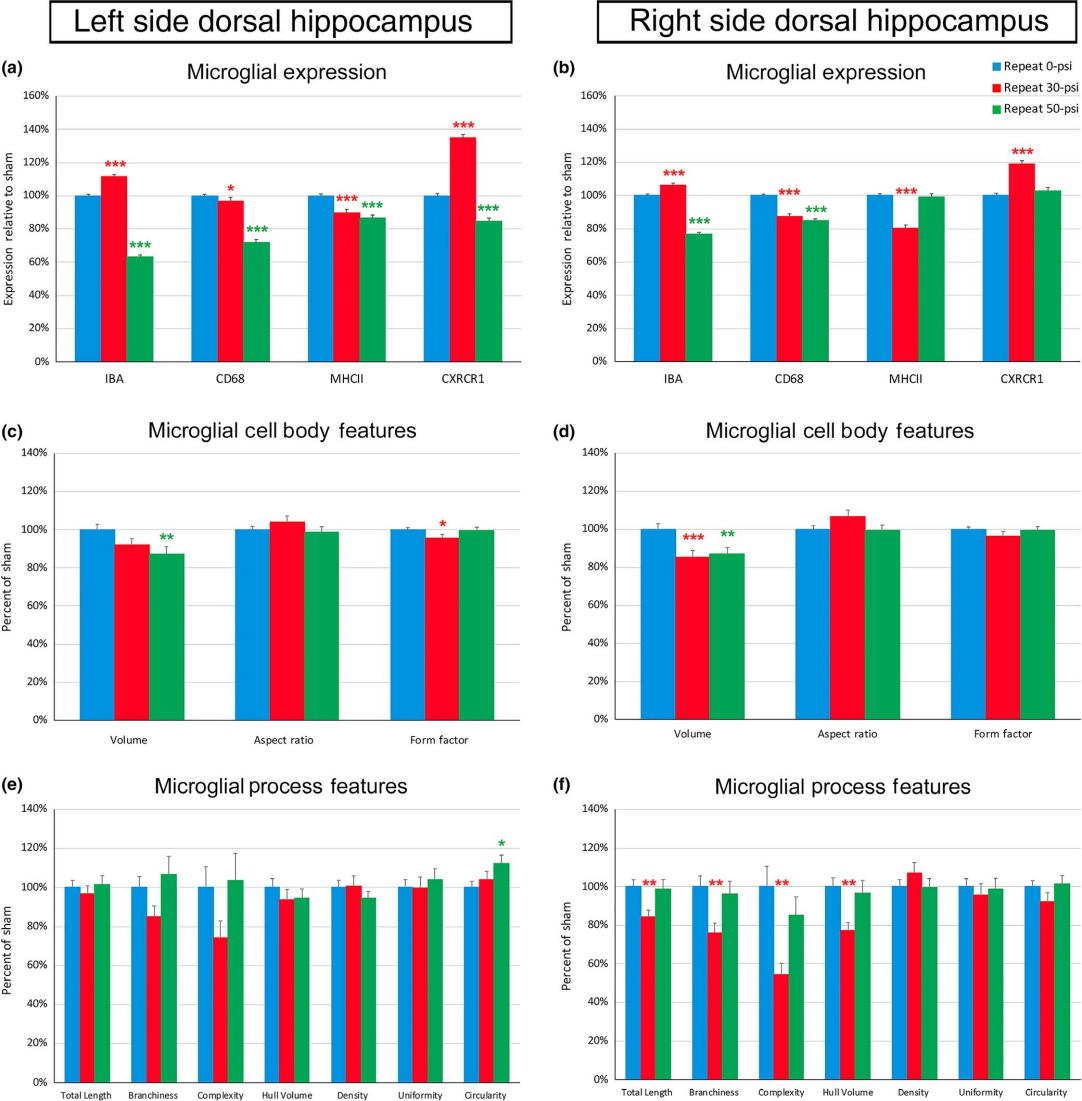
Alterations in microglial features after repeat focal cranial air blast. (a, b) Relative expression of IBA1, CD68, MHCII, and CX3CR1 by microglia in dorsal hippocampus for ~1,800 cells/group/side for IBA1 and CD68 and ~900 cells/group/side for MHCII and CX3CR1. Microglia in the repeat 50‐psi mice showed large reductions in IBA1 and CD68 expression on both sides of the dorsal hippocampus, and decreased expression of MHCII and CX3CR1 on the left. In contrast, microglia in the repeat 30‐psi mice showed increased IBA1 and CX3CR1 expression and decreased MHCII and CD68 expression on both sides of the dorsal hippocampus. (c–f) Morphometric features of ~ 60 microglia/group/side analyzed with Neurolucida 360. c and d show cell body characteristics; e and f show features of microglial processes. (c, d) Soma volume was significantly decreased for microglia on both sides of the repeat 50‐psi mice and on the right for the repeat 30‐psi mice, with a trend toward a decrease on the left (*p* = .058), together with a significant decrease in form factor. (e, f) Microglia in the repeat 30‐psi mice showed large, significant decreases in total process length, branchiness, complexity, and convex hull volume on the right. The only significant change for microglial processes in the repeat 50‐psi mice was an increase in circularity on the left. Thus, different changes were seen for microglial processes in the repeat subconcussive mice versus the repeat concussive mice, and between the two sides of the brain for the repeat subconcussive mice. Error bars are SEMs. Red asterisks indicate significant differences between the repeat subconcussive mice and the matched sham mice; green asterisks indicate significant differences between the repeat concussive mice and the matched sham mice, *p < .05, **p < .01, ***p < .001

**Figure 7:**
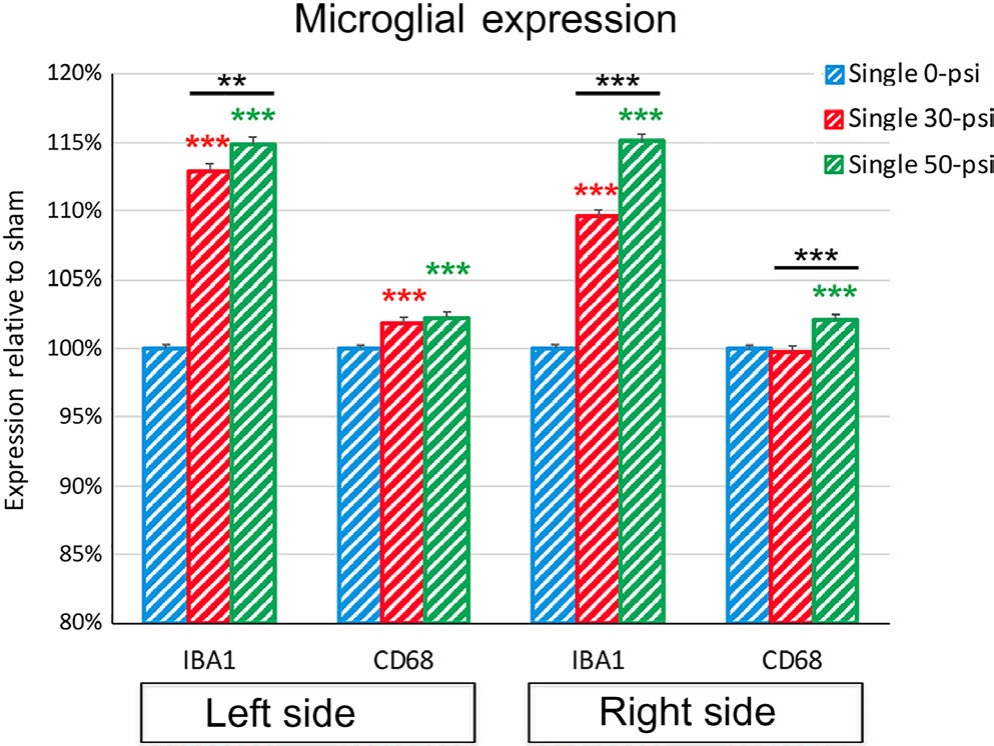
Microglial activation after single focal cranial air blast. Relative expression of IBA1 and CD68 by microglia in dorsal hippocampus 3 days after a single blast. The data are for >700 cells/group/side. Microglia in the single 50‐psi mice increased their expression of IBA1 and CD68 on both sides of the hippocampus. Microglia in the single 30‐psi mice also showed increased IBA1 expression, more so on the left than on the right (*p* < .001), but not as much on either side as in the single 50‐psi blast mice. CD68 expression was increased in the left hippocampus of the 30‐psi blast mice, but not on the right. Error bars are SEMs. Red asterisks indicate significant differences between the 30‐psi mice and the matched sham mice; green asterisks indicate significant differences between the 50‐psi mice and the matched sham mice; black asterisks indicate significant differences between the 30‐psi and the 50‐psi mice, **p < .01, ***p < .001

To further examine microglia, we triple labeled sections for immunofluorescence with IBA1 and CD68 in combination with either MHCII or CX3CR1 and examined the relative expression of these microglial markers. (Note that we initially chose these particular markers based on descriptions of primed and aged microglia in the literature, as explained below.) The IBA1 immunolabeling was used to create a mask of the individual microglial cells, and the mask was subsequently directed to the various channels to measure the OD of each channel (i.e., marker) for each cell. As explained more fully in the Methods section, to normalize the data from all the mice we analyzed this way, we rank ordered the ODs for each channel for all the microglia, and then expressed those values on a 0–1 scale, with 0.01 indicating the lowest expression level and 1.00 the highest expression. Figure 6a,b shows the data for the ~5,400 microglia analyzed this way for each side of the dorsal hippocampus, ~1,800 cells for each of the three experimental groups. Although IBA1 is considered a marker of the M0 (resting) state, IBA1 expression typically increases in the short‐term aftermath of brain injury (Cao et al., 2012; Wang et al., 2013; Kumar et al., 2016; Honig et al., 2019), and as shown in Figure 7. Unexpectedly, the expression of IBA1 was considerably reduced in microglia on both sides of dorsal hippocampus in the repeat 50‐psi mice compared with sham mice, whereas expression levels were increased bilaterally in the repeat 30‐psi mice. The CD68 protein is localized primarily to lysosomes, is implicated in phagocytosis, is typically upregulated in the pro‐inflammatory M1 state and with aging (Spittau, 2017), and is also considered to be a marker of “primed” microglia (Norden & Godbout, 2013; Norden et al., 2015; Witcher et al., 2015). However, as with IBA1 expression, CD68 expression was decreased bilaterally in the repeat 50‐psi mice. CD68 expression was also decreased in the repeat 30‐psi mice, particularly on the right side, in contrast to the bilateral increase in their IBA1 expression. The MHCII complex is involved in antigen presentation, is upregulated in the pro‐inflammatory M1 state, in the protective M2b state (Cherry, Olschowka, & O'Banion, 2014), in putatively “primed” microglia (Norden & Godbout, 2013; Norden et al., 2015; Witcher et al., 2015), and with aging (Dubbelaar, Kracht, Eggen, & Boddeke, 2018; Ojo et al., 2015; Spittau, 2017). The results showed decreased MHCII expression in the left hippocampus for both the repeat 50‐psi and the repeat 30‐psi mice, and on the right for the repeat 30‐psi mice. CX3CR1 is the receptor for CX3CL1 (i.e., fractalkine), which is expressed on neuronal cell surfaces, with CX3CR1 signaling acting to restrain microglial pro‐inflammatory responses (Deczkowska, Amit, & Schwartz, 2018). CX3CR1 expression is reduced in “primed” microglia, thereby augmenting their responses to pro‐inflammatory stimuli (Niraula, Sheridan, & Godbout, 2017; Norden et al., 2015), with aging (Stojiljkovic et al., 2019) and in microglial phenotypes associated with neurodegenerative diseases (Deczkowska et al., 2018; Dubbelaar et al., 2018; Keren‐Shaul et al., 2017; Krasemann et al., 2017). We found that CX3CR1 expression was decreased in left hippocampus for the repeat 50‐psi mice, but changed little on the right, and was increased on both sides for the repeat 30‐psi mice. Taken together, the results show different patterns of changes in expression levels for the repeat 50‐psi mice as compared to the repeat 30‐psi mice. In addition, the changes for the left side (blast side) of the repeat 50‐psi mice were generally in the same direction but larger than for the right side (contrecoup side), whereas the direction of changes in expression levels on the two sides for the repeat 30‐psi mice varied for the different markers, with IBA1 and CX3CR1 expression increased bilaterally and MHCII and CD68 expression decreased bilaterally. Importantly, the large reductions in IBA1 and CD68 levels on both sides of the repeat 50‐psi mice, together with the decreased levels of MHCII and CX3CR1 on the left, did not appear to be consistent with the “standard” profiles of activated, hyperactivated, “primed,” aged, or neurodegenerative/neurotoxic microglia. Similarly, to the best of our knowledge, the increased IBA1 and CX3CR1 expression, and the decreased CD68 and MHCII expression on both sides of the repeat 30‐psi mice is not consistent with any previously identified microglial phenotype. The implications of these results will be considered further in the Discussion.

#### 3.4.3 Microglial morphology

We next selected a minimum of 10 microglia on each side of the hippocampus from 6 mice for each experimental group, and used the IBA1 immunolabeling and Neurolucida 360 to analyze cell morphology. Numerous characteristics of cell bodies and processes were measured and the results for the repeat blast mice compared with values for the repeat sham mice, as shown in Figure 6c–f. The results showed an ~ 13% decrease in soma volume for both sides of the dorsal hippocampus in the repeat 50‐psi mice. We found a similar decrease in soma volume on the right in the repeat 30‐psi mice, but only a smaller and not significant decrease on the left. Interestingly, these changes were opposite in direction to the increased soma size characteristic of microglial activation in the short term. Further, the repeat 50‐psi mice did not exhibit any changes in cell body shape, whereas microglia in the repeat 30‐psi mice became slightly less rounded on both sides of hippocampus, but the only statistically significant change was a decrease in form factor, indicative of a more jagged soma perimeter, on the left.

Microglial processes are known to exhibit obvious morphological changes, typically becoming shorter and less ramified with activation (e.g., Verdonk et al., 2016; Walker et al., 2014), but becoming hyper‐ramified when inflammation is persistent (e.g., Beynon & Walker, 2012; Walker et al., 2014). The number of primary processes was not significantly different on either side of dorsal hippocampus for the repeat 50‐psi mice or the repeat 30‐psi mice as compared to sham (averaging 5.19 for sham, 5.27–5.53 for repeat blast). The repeat 50‐psi mice were also similar to the repeat sham mice in other microglial process features, except for an increase in the process distribution circularity score on the left side. Microglial processes in the repeat 30‐psi mice similarly showed no significant changes in left hippocampus, whereas microglia on the right exhibited large decreases in total process length, branchiness, complexity, and convex hull volume. Interestingly, the diminished territorial coverage by microglia in the right hippocampus of repeat 30‐psi mice was not associated with a decrease in the number of primary processes or by cell body enlargement, as typically occurs when microglia become activated and acquire ameboid or rod‐like morphologies (Beynon & Walker, 2012; Walker et al., 2014; Witcher et al., 2015). The expression and morphological data taken together thus suggest that repeat concussive (50‐psi) and repeat subconcussive (30‐psi) brain trauma differ in their long‐term effects on microglia, with microglia seemingly functioning aberrantly in both cases.

### 3.5 Short‐term microglial responses after single blast

Mice that have been subjected to a single 30‐psi focal cranial blast exhibit minimal axonal injury and minimal, if any, behavioral deficits (Guley et al., 2016; Heldt et al., 2014), compared to mice that had received a single 50‐psi blast. Based on the current findings of long‐term neuron loss and altered microglial phenotypes in the repeat 30‐psi mice, we decided to evaluate if single 30‐psi blasts affect microglia in the short term. To do this, we subjected mice to a single 50‐psi, 30‐psi, or sham blast, sacrificed the mice 3 days later when microglial activation is especially prominent in the white matter tracts after a 50‐psi blast (Guley et al., 2016, 2019; Honig et al., 2019), and immunolabeled for both IBA1 and CD68 to examine microglia in the dorsal hippocampus. We did not observe any of the obvious morphological changes we typically find for microglia in white matter tracts (Guley et al., 2016, 2019; Honig et al., 2019), and so we assessed microglial activation by measuring the intensity of the immunolabeling of both markers for each cell. We found that IBA1 and CD68 expression was significantly increased in microglia in the single 50‐psi blast mice, and to similar extents on the two sides of the hippocampus (Figure 7). IBA1 expression was also significantly increased in the single 30‐psi blast mice, to a greater extent in the left hippocampus than on the right (*p* = .00001), but not as much on either side as in the single 50‐psi blast mice. CD68 expression was also increased in the left hippocampus of the 30‐psi blast mice, to a similar extent as in the single 50‐psi blast mice, but showed no increase on the right. Thus, microglia appear to become activated on both sides of dorsal hippocampus after a single 50‐psi blast, whereas microglia show some activation after a single 30‐psi blast, albeit to a lesser extent than after 50‐psi blast, and more on the left than on the right.

### 3.6 Microglial profiling by hierarchical cluster analysis of expression and morphology

**Figure 8:**
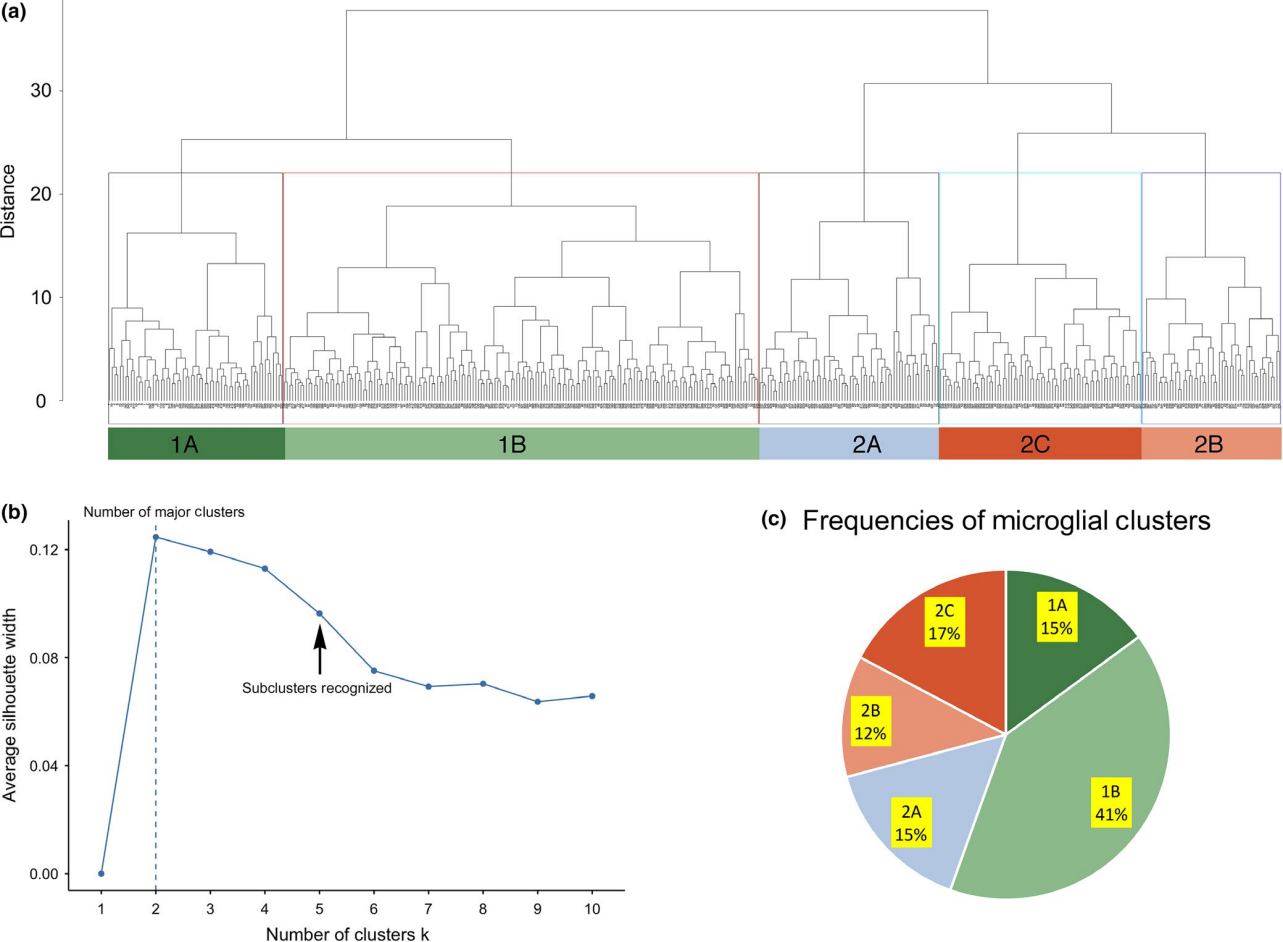
Classification of microglia according to relative expression levels and morphometric features. (a) Hierarchical cluster analysis (HCA) of microglial cells sampled from dorsal hippocampus in repeat concussive mice, repeat subconcussive mice and matched sham control mice, based on 14 parameters selected according to their multimodality index and/or prior microglia classification studies. The dendrogram is for 429 cells, performed on z‐transformed data sets, based on Euclidean distance between groups using Ward's method. The abscissa represents individual microglia and the ordinate indicates the distance between clusters. Each of the five identified clusters (1A, 1B, 2A, 2B, and 2C) is surrounded by colored lines, with the horizontal line on top showing the cut off for the five clusters. The color code for each type employed used in panel C and Figures 9, 10, 11, 12 is shown below the dendrogram. (b) Plot showing the results for the Average Silhouette Method used to determine the optimal number of clusters. The graph indicates the optimal number of clusters as 2, which we have designated as types 1 and 2. Inspection of the dendrogram and silhouette shows that these two primary types can be divided further, with type 1 divided into 1A and 1B subtypes, and type 2 divided into 2A, 2B, and 2C subtypes, with each distinctly different than the others (see Figure 9). (c) Pie chart showing the relative frequencies of the five types of microglial clusters for all 429 analyzed microglia

**Figure 9:**
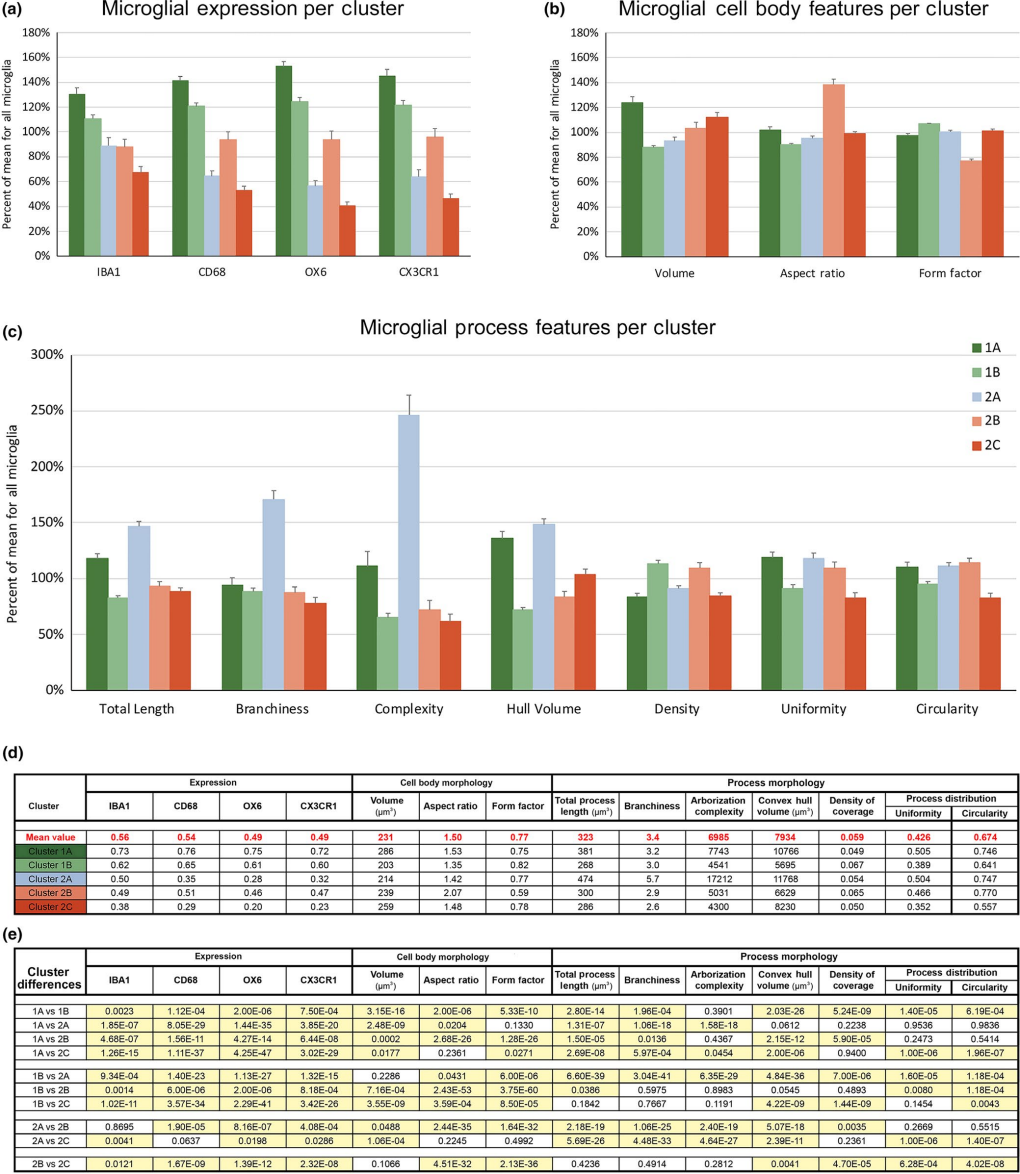
Microglial clusters. (a) Relative expression of IBA1, CD68, MHCII, and CX3CR1 for each cluster subtype. (b) Cell body characteristics for each cluster type. (c) Morphometric features of microglial processes for each cluster subtype. (d) Average values for each feature for all 429 microglia and for each of the five clusters. (e) Statistical comparisons between clusters. Comparisons yielding *p* < .05 are highlighted in yellow. Note that each cluster differed significantly in at least 9 of the 14 traits from each of the other clusters

**Figure 10:**
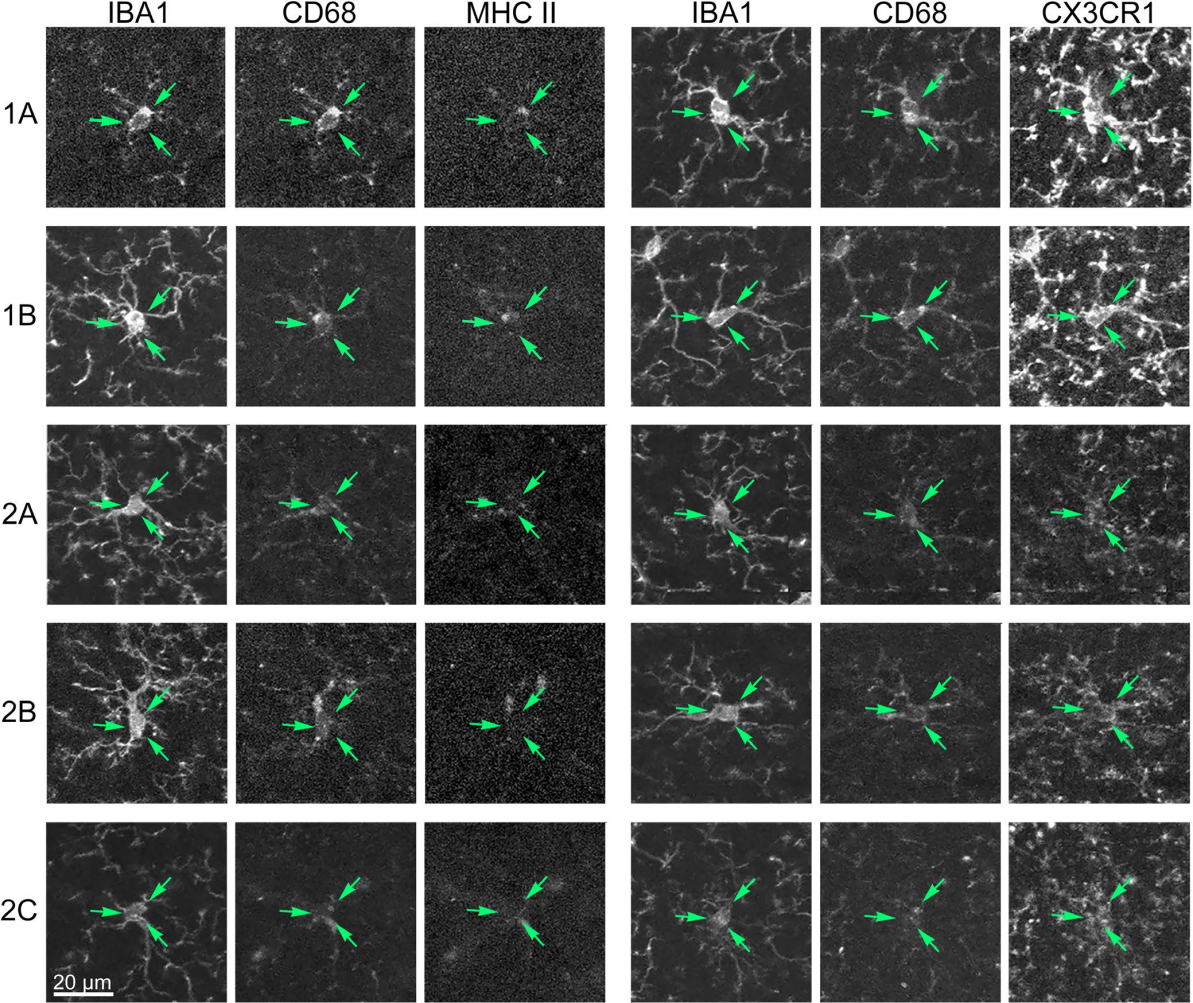
Marker expression for microglia assigned to the five subtypes of microglial clusters. For each row of images, the three panels on the left show the same cell immunolabeled for IBA1, CD68, and MHCII, and the three panels on the right show another cell immunolabeled for IBA1, CD68, and CX3CR1. The scale bar applies to all the panels

**Figure 11:**
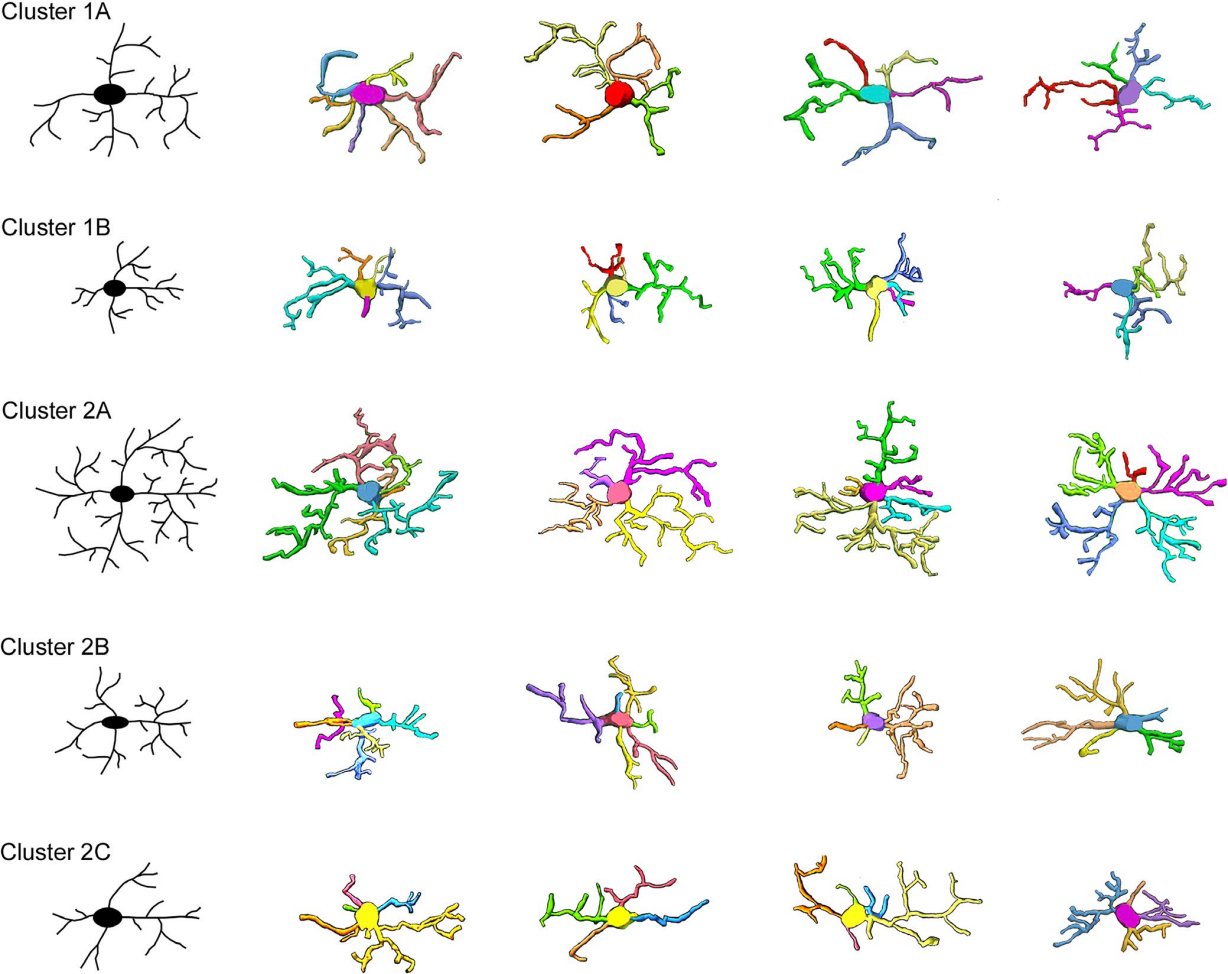
Process morphology and soma size and shape for microglia assigned to the five subtypes of microglial clusters. Each row shows a schematic on the left drawn to replicate the major morphometric features for the cluster and is followed by four examples of cells traced in 3‐dimensions with the Neurolucida 360 software, collapsed into 2‐dimensions

The analysis described above revealed that hippocampal microglia in the repeat concussive and repeat subconcussive mice differed from one another and from microglia in the sham mice at 15 months after blast. To determine if the microglia partitioned into distinct clusters, we employed hierarchical cluster analysis (HCA), using the data for 14 traits from the 429 cells we had analyzed across both sides of the hippocampus and the three experimental groups. We identified 2 major clusters (termed 1 and 2), each with subclusters (termed 1A, 1B, 2A, 2B, and 2C) that differed significantly in at least nine of the 14 traits for each pairwise subcluster type comparison (Figures 8 and 9). Cluster 1A microglia had large (286 µm^3^) cell bodies, very high expression of IBA1, CD68, MHCII, and CX3CR1, and relatively long, branchy, uniformly disposed processes. Cluster 1B microglia had small (202 µm^3^) roundish cell bodies, high expression of IBA1, CD68, MHCII, and CX3CR1, and short, dense, and relatively unbranched processes that were largely non‐uniform and non‐circular in their distribution. Cluster 2A microglia had relatively small (214 µm^3^) cell bodies, low expression of IBA1, CD68, MHCII, and CX3CR1, and very long, branchy, uniformly disposed processes. Cluster 2B microglia had average size cell bodies (239 µm^3^) that were the flattest of the five cluster subtypes (that is, had the largest aspect ratios), moderate expression of IBA1, CD68, MHCII, and CX3CR1, and relatively short, dense, unbranched, circularly distributed processes. Cluster 2C microglia had relatively large cell bodies (259 µm^3^), very low expression of IBA1, CD68, MHCII, and CX3CR, and short, unbranched, sparse processes, that were the most non‐circular and non‐uniform in their distribution, suggesting diminished surveillance of the territory covered by the processes, despite the normal complex hull volume. Figures 10 and 11 show examples of the five subtypes of microglia. Note that the 1B subtype generally resembled the 2B and 2C subtypes in process characteristics, but had considerably higher marker expression. The 2A subtype resembled the 1A subtype, but was much branchier, with a much smaller cell body, and much lower marker expression. The 1B subtype was the most common (Figure 8), whereas the 1A, 2A, 2B, and 2C subtypes were similar in their abundance across the entire sample.

**Figure 12:**
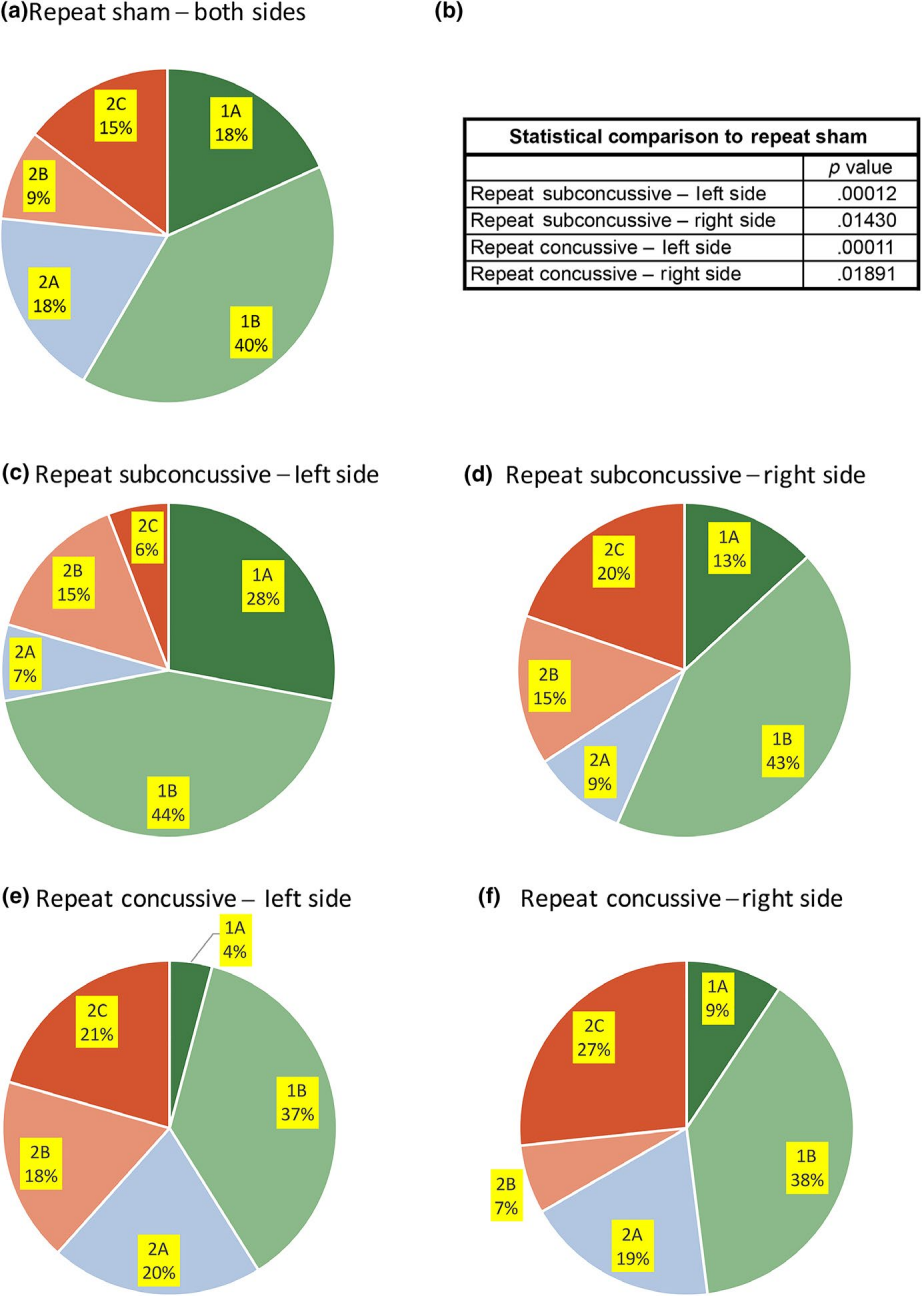
Relative frequencies of the five subtypes of microglial clusters. (a) Microglia in left and right sham dorsal hippocampus. (b) The pattern of relative frequencies for the 5 clusters differed significantly for each side of the repeat subconcussive and repeat concussive mice as compared to the respective cluster frequencies for the matched sham mice by chi‐square analysis. (c, d) Microglia in left and right hippocampus of repeat subconcussive mice. (e, f) Microglia in left and right hippocampus of repeat concussive mice. See the Results section for a description of how the pattern of relative frequencies of the subtypes of microglia for each side in the blast mice differed from their respective frequencies for the matched sham mice

The relative abundances of the five subtypes of hippocampal microglia differed among the three experimental groups, and by chi‐square the pattern of relative frequencies for each side in the blast mice differed significantly from their respective frequencies for the sham mice (Figure 12). Subtype 1B was predominant (~40%) in the sham mice, with subtypes 1A, 2A, and 2C at 15%–18%, and subtype 2B the least common, at 9%. For both sides of the repeat subconcussive mice, the abundance of subtype 2A declined (down to ~ 8%) and that of subtype 2B increased (up to 15%), and for the right hippocampus, there was also a decrease in subtype 1A and an increase in subtype 2C. For the repeat concussive mice, subtype 1A showed a large decrease and subtype 2C a large increase on both sides, and subtype 2B an increase on the left. Thus, pathological outcomes in the blast mice appeared to be most consistently associated with a reduction in subtype 1A microglia (bilaterally in repeat concussive and on the right side in repeat subconcussive mice), and increased frequencies of subtypes 2B microglia (on the left side in repeat concussive and bilaterally in repeat subconcussive mice) and 2C microglia (bilaterally in repeat concussive and on the right side in repeat subconcussive mice).

To examine how the frequency of the five subtypes of hippocampal microglia related to memory deficits and hippocampal pathology in the repeat focal cranial blast mice, we used correlation analysis. Across the 18 mice used in the HCA analysis and both sides of hippocampus, cluster 1B was positively correlated with better performance on X‐maze (more triads and fewer hard errors; *r* = .3844; *p* = .0206). To evaluate contextual fear memory, we used the frequency of microglial subtypes in the left hippocampus, based on the greater role of the left side in this subtype of memory task in mice (Shipton et al., 2014) and found an inverse correlation with the relative abundance of subtype 2B microglia (*r* = −.5030; *p* = .033). Thus, a higher prevalence of subtype 1B microglia and a lower prevalence of subtype 2B microglia appear to be associated with better spatial memory. We next evaluated the relationships between microglial subtype frequencies and neuron abundance for the right side of the hippocampus, since it tended to exhibit more overall pathology than the left. We found inverse correlations for the frequency of subtype 2B microglia with dentate granule cell abundance (*r* = −.6148, *p* = .0066) and for the frequency of subtype 2C microglia with CA1 neuron abundance (*r* = −.5121, *p* = .0298). By contrast, the frequency of subtype 1B microglia was positively correlated with CA1 neuron abundance (*r* = .5271, *p* = .0246) and that of subtype 1A microglia trended toward a positive association (*r* = .3742, *p* = .1261). Thus, subtypes 1A and 1B microglia prevalence was associated with less neuron loss and subtypes 2B and 2C prevalence with more neuron loss; the results for cluster 2A varied depending on the particular parameter. Since subtypes 1A and 1B consistently yielded positive correlations and subtypes 2B and 2C consistently yielded inverse correlations, we then combined the frequencies of subtypes 1A and 1B and of subtypes 2B and 2C and found even higher correlations for CA1 (1A and 1B: *r* = .6666, *p* = .0025; 2B and 2C: *r* = −.5963, *p* = .0090), but not for dentate. Taken together, these results suggest that subtypes 1A and 1B microglia are associated with long‐term benefit, and subtypes 2B and 2C with a long‐term adverse outcome. Why subtypes 1A and 1B microglia might be helpful and subtypes 2B and 2C microglia harmful is further considered in the Discussion.

### 3.7 Vascular abnormalities

**Figure 13:**
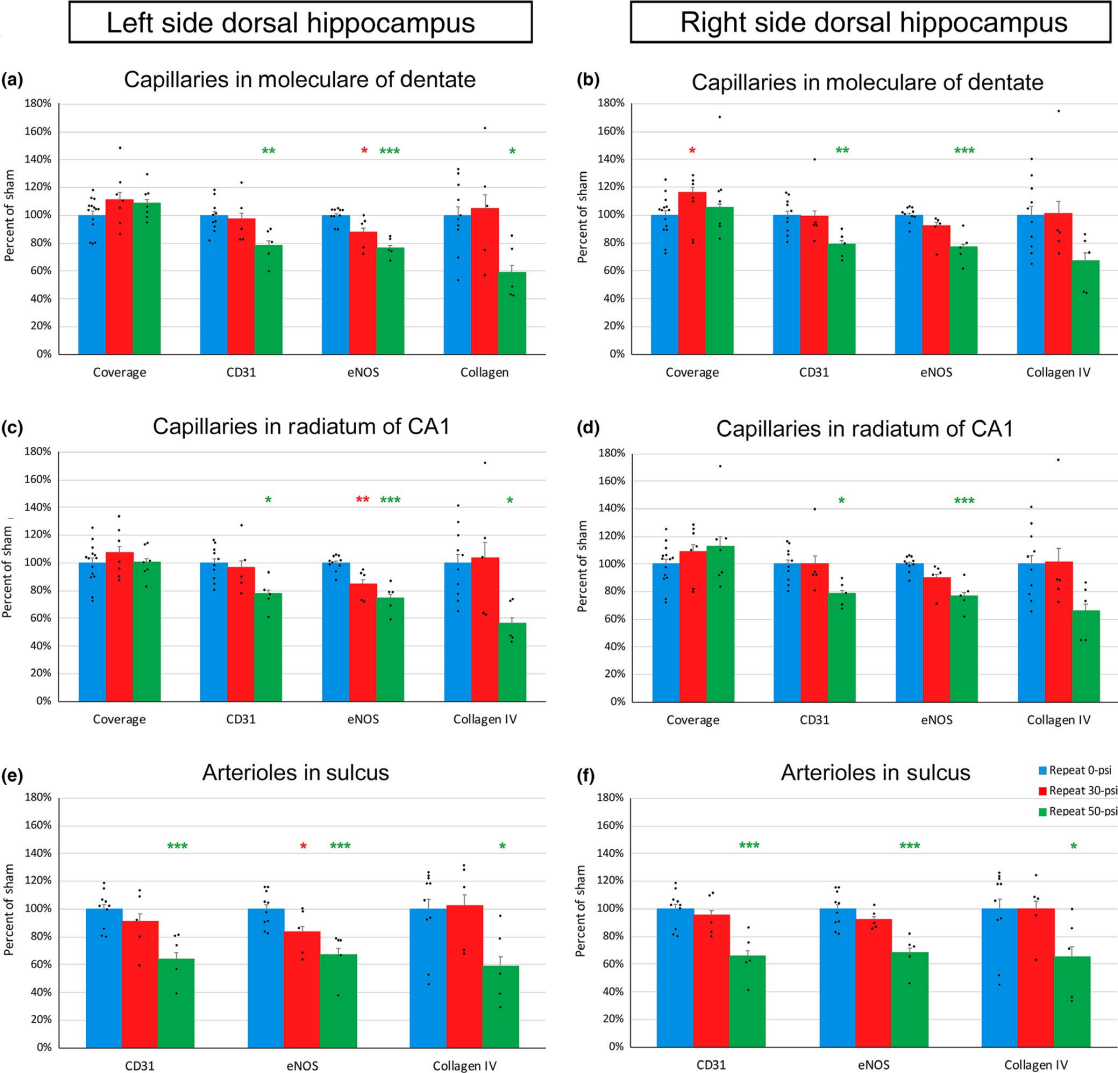
Vascular changes in dorsal hippocampus after repeat focal cranial air blast. (a–d) The relative coverage by capillaries and the relative expression of CD31, eNOS, and collagen IV in dorsal hippocampus for the left and right stratum moleculare of the dentate (a, b) and the left and right stratum radiatum of CA1 (c, d). (e–f) The relative expression of CD31, eNOS, and collagen IV by arterioles in the sulcal region. In the repeat concussive mice, the expression of CD31 and of eNOS was decreased for capillaries throughout the hippocampus, as well as for the arterioles in the sulcus. Collagen IV expression was also consistently decreased, significantly so for capillaries in left hippocampus and arterioles on both sides. By contrast, the repeat subconcussive mice showed only a significant decrease in eNOS expression for capillaries on the left side and a significant increase in vascular coverage for stratum moleculare on the right. *n* = 7/group for vascular coverage, 5/group for expression levels. Error bars are SEMs. Red asterisks indicate significant differences between the repeat subconcussive mice and the matched sham mice; green asterisks indicate significant differences between the repeat concussive mice and the matched sham mice, *p < .05, **p < .01, ***p < .001

Because vascular abnormalities may have adverse consequences for brain health, we examined blood vessels in sections of hippocampus from the repeat focal cranial blast mice and matched sham control mice at 15 months after blast (Figure 13). We started by using immunolabeling for the endothelial cell marker, CD31, to assess the abundance of vascular elements in stratum moleculare of the dentate and stratum radiatum of CA1, restricting our analysis to vessels less than 15 µm in caliber to focus on capillaries. Vascular coverage, expressed as total vessel length per unit area, was typically slightly greater in the repeat subconcussive mice than in the sham mice, but only stratum moleculare on the right side of the repeat 30‐psi mice showed a significant increase. Since the hippocampal vasculature gave the general impression of appearing impoverished in the repeat 50‐psi mice, we then assessed the expression of CD31, as well as that of eNOS and collagen IV in sections immunolabeled for these three proteins that are produced by endothelial cells. CD31, also known as platelet‐endothelial cell adhesion molecule (PECAM), functions in angiogenesis, thrombosis, and leukocyte transmigration. The enzyme eNOS, also known as nitric oxide synthase 3, catalyzes the production of nitric oxide, which acts as a vasodilator. Collagen IV is a major constituent of the basement membrane, which typically thickens in Alzheimer's disease (Nelson, Sweeney, Sagare, & Zlokovic, 2016; Thomsen, Routhe, & Moos, 2017) and in conditions of sustained vascular leak (Roy, Ha, Trudeau, & Beglova, 2010). We found significant decreases in the expression of CD31 and of eNOS for capillaries throughout the hippocampus, as well as for the arterioles in the hippocampal sulcus, in the repeat 50‐psi mice. The level of expression of collagen IV was also consistently decreased, and to a significant extent for capillaries in left hippocampus and arterioles on both sides. By contrast, the repeat 30‐psi mice showed only a few changes, specifically a significant decrease in eNOS expression for blood vessels on the left side. The decreased expression of CD31 in the repeat 50‐psi mice may reflect poor endothelial cell health, while the decreased eNOS expression is likely to indicate diminished production of nitric oxide (Park, Sorenson, & Shelbani, 2015), which might in turn result in hypoperfusion. The small increase in vascular coverage for the repeat 30‐psi mice suggests the possibility of vascular remodeling.

The combination of vascular changes we observed suggests that vascular support may have been diminished, and in turn, neuronal health, function, and survival may have been adversely affected, particularly for the repeat 50‐psi mice. Consistent with this suggestion, CD31 expression in stratum moleculare of the dentate was significantly correlated with the level of contextual fear at 13 months (*r* = .3618; *p* = .0495), suggesting that lower CD31 expression was associated with diminished spatial memory (at least for the repeat concussive mice). Moreover, the amount of vascular coverage in stratum moleculare was inversely associated with dentate neuron abundance (*r* = −.3209; *p* = .0383), suggesting that the increased vascular coverage in the repeat subconcussive mice was associated with an adverse effect on dentate neurons. Additionally, the eNOS reduction in stratum radiatum of CA1 was correlated with the loss of CA1 neurons (*r* = .4440; *p* = .0140). Vascular features also showed strong correlations with the relative abundance of the five microglial subtypes. The combined frequencies of clusters 1A and 1B were positively correlated with the overall expression of CD31, eNOS, and collagen (*r* = .5070; *p* = .0016), whereas the combined frequencies of clusters 2A, 2B, and 2C were inversely correlated with these vascular parameters (*r* = −.5070; *p* = .0005).

### 3.8 Phosphorylated tau

**Figure 14:**
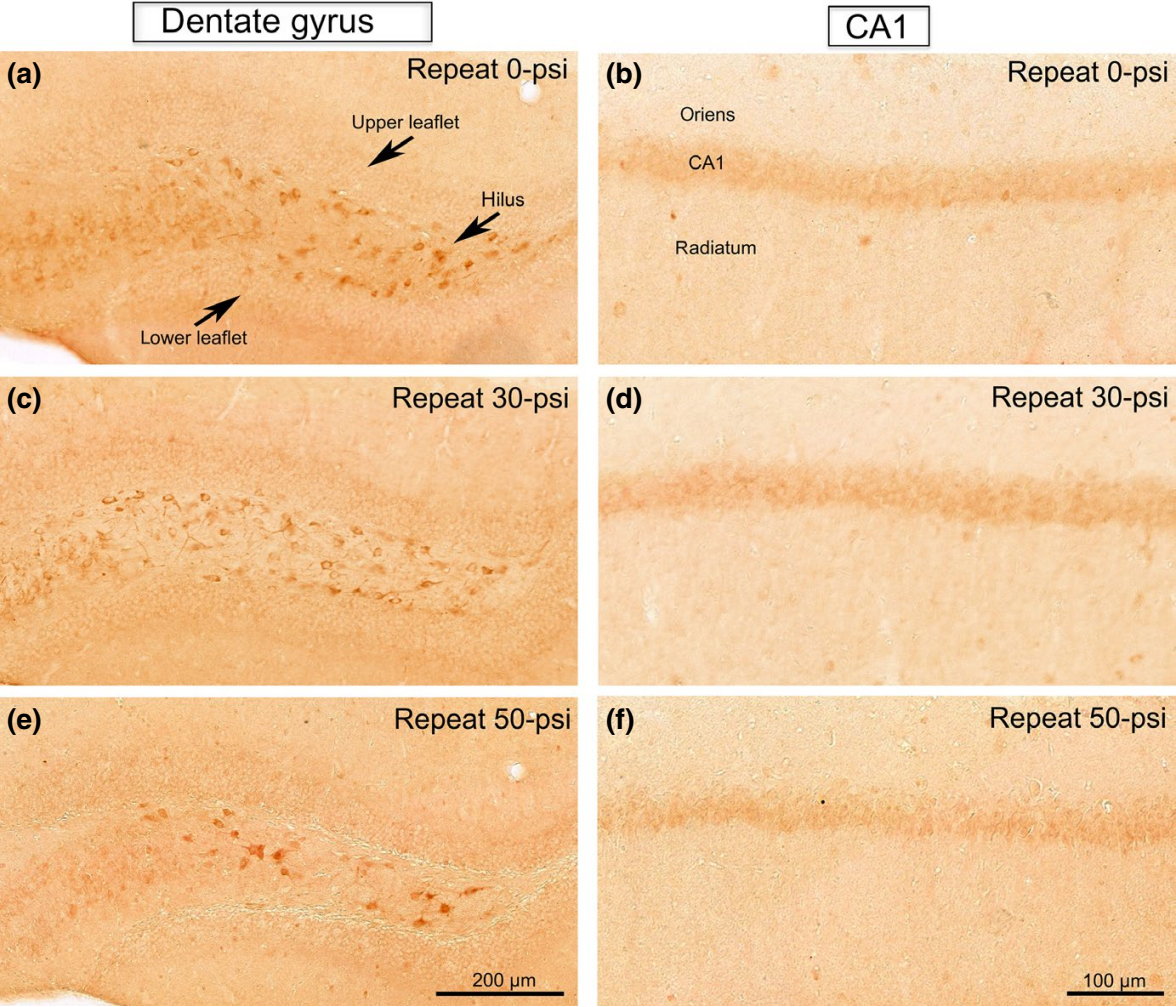
Absence of tau pathology after repeat focal cranial air blast. Images of the left side of the dorsal hippocampus immunolabeled for p‐tau using the CP13 monoclonal antibody. There is light labeling of neurons in the hilus of the dentate gyrus, pyramidal neurons in CA1, and scattered neurons in strata oriens and radiatum, but no labeling of dentate granule cells. Results for the right side were similar. The pattern and intensity of immunolabeling were similar for repeat concussive mice, repeat subconcussive mice and matched sham control mice, thereby indicating that the mice we subjected to repeat focal cranial blast did not exhibit p‐tau pathology. Medial is to the right and dorsal to the top for all images. Scale bar in e applies to a, c, and e; scale bar in f applies to b, d, and f

Due in large part to the prominent p‐tau pathology in CTE, numerous studies of repeat TBI have assessed tau phosphorylation. We immunolabeled sections from six repeat concussive mice, four repeat subconcussive mice, and nine matched sham mice with the CP13 monoclonal antibody to detect tau phosphorylated at Ser202 (Figure 14). Neurons in the hilus of the dentate gyrus were lightly labeled, as were pyramidal neurons in CA1 and scattered neurons in strata oriens and radiatum. We also observed light staining of pyramidal neurons in CA2‐4. The extent of labeling was similar for each of these regions on both sides of the brain and across the three groups of mice. We did not detect notably altered immunostaining in the overlying cerebral cortex in any of the repeat blast mice, nor did we see CTE‐like perivascular p‐tau pathology. Note that the pattern and intensity of the immunostaining we found in both repeat blast and sham mice resembled that shown for both single impact and sham aged mice overexpressing human tau (h‐tau) by Ojo et al. (2013).

## 4 DISCUSSION

In the work reported here, we found that repeat concussive and repeat subconcussive trauma both engendered long‐term processes involving neurodegeneration and leading to memory decline. The mice with repeat concussive brain injury showed spatial memory deficits at 13–14 months after the last trauma, and, surprisingly, the repeat subconcussive mice exhibited even greater spatial memory deficits, although neither group had demonstrated significant impairments at 4 months after injury. By contrast, the mice that had received a single injury, whether at a concussive or subconcussive level, did not show any deficits in spatial memory at either the early 4‐month or later 12‐month time point, as ascertained by the level of contextual fear they exhibited. Both repeat concussive and repeat subconcussive head trauma caused neuron loss in dentate gyrus and CA1 of hippocampus, with substantial loss on the contrecoup as well as the coup side in both cases. Microglial profiling by HCA partitioned microglia into five subtypes that differed in their relative abundance in hippocampus of repeat concussive and repeat subconcussive mice, as compared to matched sham mice. Notably, microglial subtypes characterized by deramification and de‐activation and thus likely to be dysfunctional were overrepresented in mice that had experienced repeat trauma and were associated with memory deficits and hippocampal neuron loss. We also found vascular abnormalities in dorsal hippocampus, particularly in the repeat concussive mice, that were linked to the microglial abnormalities, memory deficits, and hippocampal neuron loss and that were suggestive of impaired blood flow.

### 4.1 Characteristics of our focal cranial air‐blast model and comparisons to other animal models

A consistent histopathological feature of mild TBI in experimental animals and concussion in humans is widespread axonal injury, which occurs without any accompanying skull damage, brain contusion, or hemorrhaging. In the model we developed, the mice are shielded, stabilized, and cushioned, so as to protect their bodies and minimize head movement. We previously reported that air blasts delivered to the left side of the cranium that exceed 60‐psi in amplitude often result in subdural and/or intracranial bleeding, but the 50‐psi blasts used in the work reported here produce no overt brain damage (Guley et al., 2016). We have previously hypothesized that some of the energy of the blast force is used in displacing the animal's head (<1 cm during the ~15 ms duration of the blast), while the remaining energy produces a pressure wave that propagates across the skull and through the brain parenchyma (Guley et al., 2016). Due to its viscoelastic nature, this pressure wave causes rapidly alternating compression and stretching of brain tissue that, in turn, results in axonal injury. Single 50‐psi focal cranial air blasts damage axons in several white matter tracts, as indicated by the presence of swollen axonal bulbs during the first few days and the later degeneration of injured axons (Guley et al., 2016, 2019; Honig et al., 2019). The ensuing pathological events, for example, microglial activation, and the motor deficits that we observe using our focal cranial blast model are largely similar to those seen after brain injury produced using other types of experimental approaches, as further discussed below.

Although the different types of mild TBI in animal models and concussions in people are associated with variation in the types and duration of functional deficits, these differences are likely to stem more from differences in the axis, direction, amplitude, and duration of the forces than with the actual nature of the biomechanical forces themselves (Blennow et al., 2016; Namjoshi et al., 2013). Impact alone, impact‐acceleration, acceleration/deceleration, and explosive blast have all been shown to produce axonal damage (Browne, Chen, Meaney, & Smith, 2011; Chen, Gu, Zhu, & Feng, 2020; Hernandez et al., 2018; Longhi et al., 2005; Mouzon et al., 2012; Zakaria, Kallakuri, Bandaru, & Cavanaugh, 2012) that is very similar to what we find with focal cranial air blast. This observation is consistent with biomechanical, computer simulation, and finite element modeling studies demonstrating that blasts, head impact, and acceleration/deceleration all generate intracranial pressure gradients that deform brain tissue (Beckwith et al., 2018; Madhukar & Ostoja‐Starzewski, 2019; Tagge et al., 2018; Taylor, Ludwigsen, & Ford, 2014; Wright, Post, Hoshizaki, & Ramesh, 2013).

It should also be noted that the focal cranial air blast approach we use, nonetheless, differs from explosive blast injury, which is a major cause of mild TBI for military personnel and can result in axonal injury in the short‐term and neurodegeneration in the long‐term (Blennow et al., 2016). Explosive blast injury is most frequently modeled in rats and mice by the use of shock tubes (see review by Chandra & Sundaramurthy, 2015). Blast experiments are, however, conducted in diverse ways (using different types of shock tubes and gases, and with variations in the location, distance and orientation of the animal inside or outside the shock tube, shielding of the animal, stabilization of the head). Nonetheless, they typically produce a complex injury with the blast wave affecting the entire body and/or causing head acceleration/deceleration, and frequently damaging capillaries and producing blood‐brain barrier breakdown (BBB) in the short‐term (Agoston, 2017; Blennow et al., 2016; Chen, Huang, & Constantini, 2013; Elder et al., 2015). Thus, shock tube blast differs from the other types of injury discussed above in that whole body and vascular effects contribute to the injury process. One important consequence of blast injury being more complex is that it becomes additionally challenging to effectively study disease progression and relate it to underlying pathogenic mechanisms.

### 4.2 Progressive spatial memory and neuron loss after repeat head trauma

We found ~ 10% bilateral neuron loss in CA1 15 months after repeat concussive TBI and ~ 14% bilateral loss after repeat subconcussive trauma. For granule cells of the dentate gyrus, we observed ~ 8% bilateral cell loss after repeat concussive trauma, and a similar extent of loss for repeat subconcussive trauma on the right side, but a smaller (<5%) and not significant reduction on the left side. The lower magnitude of neuron loss in dentate gyrus, as compared to CA1, may stem from neurogenesis in the dentate, which serves to replenish the granule cells. Nonetheless, the diminished abundance of doublecortin+ immature neurons in repeat concussive and repeat subconcussive mice, suggesting reduced granule cell replenishment, may help explain why dentate neuron loss still occurred. The nearly identical extent of neuron loss bilaterally suggests that the higher “concussive” blast force has similar effects on the two sides of the brain, at least for juxtaposed pairs of structures such as the dorsal hippocampus, cortex, and striatum (but not basolateral amygdala; see Bu et al., 2016 for further discussion). By contrast, the neuron loss we observed with repeat subconcussive trauma was unexpected, and likely exceeded what results from a single blast and/or is found at an earlier time point after repeat blast. Further, the significant loss only on the contrecoup side, at least in the case of dentate, suggests that the presumably lesser contrecoup forces initiate processes that are not triggered (or perhaps are inhibited) on the coup side. As will be further discussed below, the effects on microglia were also more prominent on the contrecoup side in the repeat subconcussive mice.

Most surprisingly, we found that repeat subconcussive injury produced greater spatial memory deficits than repeat concussive injury, with the deficits following both types of injury becoming evident many months after the initial trauma. Although hippocampal neuron loss may have contributed to these spatial memory deficits (Christian, Song, & Ming, 2014; Gonçalves, Schafer, & Gage, 2016; Sierra et al., 2014), we did not find significant correlations between neuron abundance and performance on the memory tests. However, other hippocampal abnormalities that we did not evaluate may have been present and may help explain the memory loss, for example, disturbances in neuronal electrophysiological properties or synaptic connectivity. The latter possibility is of interest given the role of microglia in synaptic maintenance (Hong, Dissing‐Olesen, & Stevens, 2016) and the alterations in microglia we found in the repeat injury mice.

### 4.3 Microglial abnormalities

Microglia in the dorsal hippocampus of the repeat TBI mice exhibited a variety of abnormalities. For example, microglia in repeat subconcussive mice generally possessed smaller, flatter cell bodies with less branching than microglia in sham mice. Microglia in repeat concussive mice typically had smaller cell bodies than in sham mice but normal branching and expressed low levels of the markers we examined (IBA1, CD68, MHCII, CX3CR1). The HCA analysis revealed five major dorsal hippocampal microglial subtypes across the three groups, with an overrepresentation of microglia characterized by low marker expression and/or reduced branching (subtypes 2B and/or 2C) in repeat injury mice and a prevalence of microglial subtypes characterized by high marker expression and moderate branching (subtypes 1A and 1B) in sham mice. Importantly, the relative abundance of subtypes 2B and 2C relative to subtypes 1A and 1B was correlated with poorer spatial memory performance and greater neuron loss. Moreover, the repeat concussive and the repeat subconcussive mice differed in which deleterious microglia subtype was more common, with the 2C subtype more prevalent bilaterally in the repeat concussive mice but only on the right in the repeat subconcussive mice, and the 2B subtype more prevalent on both sides in the repeat subconcussive mice.

Many studies have shown that microglial morphology and expression are indicative of their functional characteristics. Microglia are normally in a resting M0 surveillance state, with long ramified processes by which they monitor their environment. With signaling initiated by brain disease or trauma (Bailes et al., 2013; Coughlin et al., 2015, 2017; Faden & Loane, 2015), microglia become activated, exhibiting morphological changes including cell body enlargement and process shortening (e.g.,Verdonk et al., 2016; Walker et al., 2014). Yamada and Jinno (2013) used HCA to categorize microglia in the hypoglossal nucleus based on their morphology at 3, 7, 14, and 28 days following axotomy of the hypoglossal nerve in mice, and identified four subtypes: 1) Ramified cells that were common in control mice and a month after axotomy; 2) cells with small somas and short thin processes called “small ramified cells” that were most abundant 2 weeks after axotomy; 3) hypertrophied microglia with thickened processes that were most abundant at 3 days; and 4) “bushy cells” with very short thick processes that were most abundant at 7 days. Subtypes 2–4 were all regarded as on a continuum of activation characterized by process shortening and thickening. We did not observe their “hypertrophied” subtype 3 microglia or “bushy” subtype 4 microglia, both regarded as typifying highly activated microglia, in all likelihood because the initial trauma in our studies was less severe and far in the past. Their subtype 1 microglia appear to represent the M0 state and resemble our subtype 2A microglia, while their subtype 2 microglia appear to be mildly activated and resemble our 1A and 1B subtypes. In another study, Fernández‐Arjona et al. (2017) classified microglia in mouse hippocampus 2, 4, and 12 hr after intraventricular injection of an inflammatory agent, and identified 4 major subtypes. Their subtype 2 microglia, which predominated in control mice, would be regarded as in the M0 state, and resemble our subtype 2A and their sparsely branched type 3 resembles our subtypes 1A and 1B. The suggestions that our subtype 2A microglia are M0 surveillant, whereas our subtypes 1A and 1B microglia are mildly activated, imply that mildly activated microglia predominated in hippocampus of the sham mice in our study. This, in fact, is consistent with findings showing that age increases overall microglial activation (Spittau, 2017). Notably, none of our HCA cluster subtypes appeared to correspond to either of the two highly activated subtypes of microglia described by Fernández‐Arjona, that is, “bushy” type 1 microglia or the unbranchy, subtype 4 microglia.

Thus, our subtypes 2B and 2C microglia do not resemble microglial phenotypes defined in prior HCA studies, nor do we know of any correspondents from published studies that have employed transcriptomic analysis. Neither subtype obviously resembles the neurodegenerative/toxic (MGnD) microglial phenotype identified in transgenic mouse models of neurodegenerative diseases and human AD brains, which exhibits upregulation of both pro‐inflammatory and neuroprotective genes (Krasemann et al., 2017), the degenerative disease‐associated microglial (DAM) phenotype, which exhibits downregulation of CX3CR1 and MHCII but high expression levels of CD68 (Keren‐Shaul et al., 2017), or the microglial phenotype identified in a transgenic mouse model of severe neurodegeneration that shows high expression levels of MHCII (Mathys et al., 2017). Note that the phenotypes identified in these transcriptome‐profiling studies may, in fact, be the same, given their overlapping features.

### 4.4 Role of microglia in neuropathology after repetitive head trauma

Microglia are thought to play a role that evolves over time following brain trauma. Shortly after mild TBI, microglia are activated predominantly to the M1 state (Donat et al., 2017; Guley et al., 2019; Honig et al., 2019; Loane & Kumar, 2016). M1 microglia produce cytotoxic and pro‐inflammatory proteins that exacerbate neuronal damage directly, as well as indirectly through disruptive effects on the BBB and vascular function (Bailes et al., 2013; Elder et al., 2015; Glass, Saljo, Winner, Marchetto, & Gage, 2010; Loane, Kumar, Stoica, Cabatbat, & Faden, 2014; Smith, Das, Ray, & Banik, 2012). Activated microglia can also adopt an “alternative” M2 activation state, which is associated with tissue maintenance and repair, typically as M1 activation wanes (Donat et al., 2017; Loane & Kumar, 2016; Witcher et al., 2015), with microglia largely returning to the M0 state a week or two after the initial TBI event (Cherry et al., 2014; Wang et al., 2013).

Some investigators have proposed that microglia contribute to the long‐term adverse effects of TBI by being driven, under some circumstances, to a state of persistent activation, sometimes referred to as the “primed” state, characterized by long‐lasting transcriptional changes (Dubbelaar et al., 2018; Raj et al., 2014) and exaggerated pro‐inflammatory responses to subsequent challenges (Loane et al., 2014; Mouzon et al., 2014; Niraula et al., 2017; Witcher et al., 2015). Persistent, exaggerated pro‐inflammatory microglial responses would, in turn, perturb both homeostatic processes (Dubbelaar et al., 2018; Pluvinage et al., 2019) and vascular function, thereby adversely affecting neuronal health (Dudvarski Stankovic, Teodorczyk, Ploen, Zipp, & Schmidt, 2016; Privratsky & Newman, 2014; Stanimirovic & Friedman, 2012; Woodfin, Voisin, & Nourshargh, 2007; Zhao, Nelson, Betsholtz, & Zlokovic, 2015). Associated with their increased reactivity, “primed” microglia express high levels of IBA1, CD68, and MHCII (Niraula et al., 2017; Norden & Godbout, 2013; Norden et al., 2015; Raj et al., 2014; Witcher et al., 2015). “Primed” microglia are consistently described as possessing large cell bodies, but descriptions of their process characteristics vary (Norden et al., 2015; Raj et al., 2014; Torres‐Platas et al., 2014) and identification based solely on morphological criteria is thus not possible (Raj et al., 2014). Despite our initial expectation that “primed” microglia would be overrepresented in our repeat TBI mice, we found this was not the case. In fact, the deleterious 2B and 2C microglia exhibited low expression of IBA1, CD68, and MHCII, much lower than the “mildly activated” subtypes 1A and 1B microglia, and their cell bodies were not enlarged. Thus, the progressive memory decline exhibited by our repeat TBI mice was not associated with microglia being in a “primed” or “persistently activated” state at the time the mice were euthanized (15 months after the last blast), but rather with microglial de‐activation and dysfunction (Hefendehl et al., 2014).

Microglia that are continuously exposed to activating signals, for example, β‐amyloid in AD (Go, Kou, Lim, Yang, & Fukuchi, 2016) or during chronic unpredictable stress (Kreisel et al., 2014), can become moribund and eventually die. The trophic and protective support that microglia can provide (for example, by means of secreted brain‐derived neurotrophic factor) is gradually diminished, adversely affecting neuronal function, health, and survival (Hickman, Izzy, Sen, Morsett, & Khoury, 2018; Ojo et al., 2015; Stojiljkovic et al., 2019), as well as dentate granule cell neurogenesis (De Lucia et al., 2016; Kreisel et al., 2014; Tay, Savage, Hui, Bisht, & Tremblay, 2017). This may be especially problematic for the aged brain, in which microglia are M2 polarized and thus more neuroprotective (Hickman et al., 2013) and their loss thus of greater consequence. The loss of trophic support, together with the diminished ability to maintain homeostasis (Dubbelaar et al., 2018; Pluvinage et al., 2019), would then be the likely consequences of the decreased microglial abundance we found for dorsal hippocampus in the repeat subconcussive mice.

Microglia that are characterized by few, short, relatively unbranched processes, some of which may be beaded, fragmented or gnarled and thus considered “dystrophic,” have been identified in the aged brain (Bachstetter et al., 2015; Ojo et al., 2015; Streit, Braak, Xue, & Bechmann, 2009; Streit et al., 2014; Tischer et al., 2016). Although these dystrophic microglia have sometimes been referred to as “senescent” (e.g., Streit et al., 2009; Streit et al., 2014; Tischer et al., 2016), whether they fulfill the molecular criteria for “senescence” (Khan et al., 2015; Stojiljkovic et al., 2019), exhibiting irreversible proliferation arrest and shortened telomeres, for example, has not been ascertained, to the best of our knowledge. “Senescent” microglia reportedly tend to have large cell bodies, and express high levels of CD68 and MHCII, as well as IL1β (Safaiyan et al., 2016; Stojiljkovic et al., 2019). Our deleterious subtypes 2B and 2C microglia are thus not senescent, as their somas are small and their expression of CD68 and MHCII was low. They may, however, represent specific stages of microglial deramification, de‐activation, and dysfunction, with subtype 2B microglia showing higher levels of CD68 and MHCII expression and thus some signs of activation, as compared to subtype 2C (and subtype 2A) microglia, albeit less than subtypes 1A and 1B. Whether subtypes 2B and/or 2C microglia were preceded by an earlier stage of heightened and persistent activation that led to microglial dysfunction is uncertain. It is nonetheless clear that the 2B and/or 2C microglial phenotypes are associated with neuronal loss and memory decline in our repeat TBI mice, based on significant correlations between these parameters and the abundance of 2B and 2C microglia.

Hippocampal microglia appear to be especially susceptible to age‐related and activation‐related decline compared with microglia in other parts of the forebrain, thus likely rendering hippocampal neurons especially vulnerable to the challenges initiated by TBI (Askew et al., 2017; Grabert et al., 2016; Khan et al., 2015). The effects of prior brain trauma on microglia may be particularly pronounced in the pro‐inflammatory milieu of the aging brain when their protective role is most needed (Luo, Ding, & Chen, 2010; Niraula et al., 2017; Ojo et al., 2015; Ziebell et al., 2017). This may explain the late emergence of memory loss in people with a history of concussive and subconcussive brain trauma and in the studies described here. One possible explanation for how repetitive subconcussive blast could initiate a process that leads to a worse long‐term outcome than repetitive concussive blast is that microglia may be “engineered” so that the more severe concussive injury causes self‐terminating processes that do not occur after a subconcussive injury (Deczkowska et al., 2018; Frank, Fonken, Watkins, & Maier, 2019). For example, in the repeat concussive mice, microglia may initially become activated, but return to the M0 state (Cherry et al., 2014; Wang et al., 2013), perhaps as a consequence of the action of one or more checkpoint mechanisms (Deczkowska et al., 2018). Alternatively or in addition, adaptive changes triggered by mild TBI may potentially decrease the risk for cumulative damage with repeat, widely spaced concussive trauma, akin to the protection provided by ischemic pre‐conditioning (Bolton‐Hall, Hubbard, & Saatman, 2019). In contrast, the initial perturbation produced by a subconcussive blast may be too small to engage any of the checkpoint mechanisms and microglia may instead remain in a mildly activated phenotype after repeated subconcussive injury that ultimately leads to their functional decline and depletion (Khan et al., 2015; Norden et al., 2015). Note that we found microglia on both sides of the dorsal hippocampus indeed showed short‐term responses after a single subconcussive blast, albeit less than after a single concussive blast, indicating that microglia respond to both concussive and subconcussive trauma, but in different ways. How microglial behavior evolves differentially over time following repeated injury spaced one week apart, and whether it evolves differently with different spacings needs to be determined to better understand the long‐term consequences of concussive and subconcussive head trauma for neurodegeneration and memory loss.

### 4.5 Vascular abnormalities

Our analysis also showed that vascular pathology is associated with repeat head trauma and is likely to contribute to hippocampal pathology and memory deficits. The reduced blood flow implied by the decreased expression of CD31 and eNOS in the repeat concussive mice and to a lesser extent by the decreased eNOS expression on the left in the repeat subconcussive mice would result in the hippocampus being subjected to chronic hypoxic stress (Liu & Huang, 2008; Park et al., 2015). Importantly, vascular dysfunction is thought to contribute to cognitive decline in TBI, AD, and aging (Lynch et al., 2016; Nelson et al., 2016; Ojo et al., 2015; Pop & Badaut, 2011; Zhao et al., 2015), and eNOS deficiency in mice has been linked to cognitive impairment (Tan et al., 2015). Moreover, vascular leakiness may be a consequence of CD31 reduction, and in turn would disturb the ionic neuronal milieu and thereby impair neuronal function (Lertkiatmongkol, Liao, Mei, Hu, & Newman, 2016; Privratsky & Newman, 2014; Zhao et al., 2015). Our data suggest that the shift toward subtypes 2B and 2C microglia in the repeat head trauma mice is linked to the vascular abnormalities. Microglia, particularly those in the M2 state, can release vascular endothelial growth factor and transforming growth factor beta and thereby help maintain blood vessels (Dudvarski Stankovic et al., 2016). The impaired functioning of subtypes 2B and 2C microglia may thus contribute to the vascular abnormalities we observed in the repeat concussive and the repeat subconcussive mice.

### 4.6 Animal models of the long‐term effects of repeat TBI

#### 4.6.1 Effects on memory

Although memory loss has been reported in the days to weeks after a single mild TBI event (e.g., Cho, Sajja, Vandevord, & Lee, 2013; Creed, DiLeonardi, Fox, Tessler, & Raghupathi, 2011; Hoskison et al., 2009; Koliatsos et al., 2011; Watanabe et al., 2013), relatively few studies have examined more long‐term effects. Studies of repeat mild TBI using various blunt injury approaches in mice have also tended to examine relatively short‐term effects, many seeking to determine the time frame over which separate TBI events synergize to exacerbate the injury. These have typically shown that TBI events spaced more than 3–5 days apart do not synergize in the short term (e.g., Longhi et al., 2005; Prins et al., 2013; Weil et al., 2014), and that closely spaced repeat mild TBI has greater adverse consequences than single events or more widely spaced multiple events (Bolton‐Hall, Joseph, Brelsfoard, & Saatman, 2016; Buckley et al., 2015; Kane et al., 2012; Laurer et al., 2001; Mouzon et al., 2012; Prins, Hales, Reger, Giza, & Hovda, 2010; Uryu et al., 2002). Among the few studies published, repeat blast has been reported to produce more severe outcomes than single blast (Agoston, 2017; Effgen et al., 2016; Wang et al., 2011), but the effect of varying the time between injuries has received little attention and nearly all studies have been limited to the first week after injury.

Several studies of closely spaced repeat mild TBI have shown memory deficits for up to 6 months (Chen, Desai, & Kim, 2017; Luo et al., 2014; Lynch et al., 2016; Ojo, Mouzon, Algamal, et al., 2016; Petraglia, Plog, Dayawansa, Dashnaw, et al., 2014; Xu et al., 2016), and a few studies have shown memory deficits persisting beyond 6 months (Cheng et al., 2019; Ferguson et al., 2017; Gangolli et al., 2019; Mouzon et al., 2014). Comparisons to our own work are hindered, however, by the differing approaches used to produce mild TBI and the differing number and spacing of the repeated injuries (reviewed in Hoogenboom, Branch, & Lipton, 2019). Limiting the discussion below to studies employing closed‐head injury in mice and examining memory deficits, the key issues are as follows: 1) Is there prior evidence that an interval of a week or longer between successive TBI events yields an adverse outcome? 2) Is there prior evidence that repeat injury can lead to a progressive neurodegenerative process? 3) What pathogenic mechanisms mediate the long‐term injury?

With regard to TBI event spacing, Whalen and coworkers used a weight drop model and showed that mice that had received five head impacts spaced a day apart exhibited significant spatial memory deficits (as assessed by Morris water maze) 1, 6, and 12 months later. When the impacts were spaced a week apart, similar deficits were observed for at least as long as 6 months, whereas no deficits were seen when the five impacts were spaced 2 weeks or a month apart (Mannix et al., 2013; Meehan, Zhang, Mannix, & Whalen, 2012). Studies employing impactor devices have also used varying numbers of impacts and spacing. Winston et al. (2016) subjected mice to 30 total impacts, once a day 5 days a week for 6 weeks, but found normal performance in the Morris water maze both at 1 week and 1 year after the last impact. In contrast, several studies by the Crawford group demonstrated spatial memory deficits after repeat TBI that endured 6 months and beyond. For example, Mouzon et al. (2014), Ferguson et al. (2017), and Mouzon et al. (2017) all used the same paradigm of 5 total impacts, each 2 days apart, and tested performance on a Barnes maze. Repeat impact mice showed greater mean distance to target than single impact and sham mice at 6, 12, 18, and 24 months. Although escape latency and probe trial performance were poorer than in single impact mice at 6 and 12 months, they were similar to that in single impact mice at 18 and 24 months, suggesting some recovery in the repeat impact mice. Lynch et al. (2016) used more widely spaced impacts (twice a week) for 3 months and observed spatial memory deficits on Barnes maze at 1 and 6 months post‐injury, but did not examine later time points. Studies by the Wyss‐Coray group, using a different impact device and injury protocol, delivered 3 impacts, one a day for 3 days, and demonstrated contextual fear spatial memory deficits, but normal Y‐maze performance, at 6 months (Luo et al., 2014). Other researchers have combined impact and acceleration/deceleration brain injury using the closed‐head impact model of engineered rotational acceleration (CHIMERA), repeating the injury on a daily basis for 2 days (Cheng et al., 2019) or for 3 days (Chen et al., 2017), and found a nearly significant spatial memory impairment in Barnes maze at 8 months and a significant impairment in Morris water maze at 6 months, respectively, neither of which were progressive. Gangolli et al. (2019) used CHIMERA at either a concussive or subconcussive level, repeating the injury once a day for 20 consecutive days, and studied the outcomes 1 month, 3 months, and a year after the last injury. They found that repeat concussive, but not subconcussive, injury produced memory deficits in Morris water maze that were present acutely and persisted, but did not worsen over time. No other studies, to the best of our knowledge, have assessed the effect of repeat subconcussive injury. Our study thus appears to be the first showing that repeat subconcussive injury can have adverse long‐term effects, and the first to report progressive cognitive impairment following both repeat subconcussive and concussive injury.

#### 4.6.2 Possible mechanisms underlying progressive harm

Three processes that could be the basis of the progressive harm resulting from repeat brain injury have been noted previously: tau phosphorylation and accumulation, microglial activation, and cerebrovascular dysfunction. The focus on p‐tau driven pathogenesis stems from the prominence of p‐tau pathology in CTE, as well as in AD, although precisely which forms of p‐tau are neurotoxic is uncertain (Kimura, Sharma, Ishiguro, & Hisanaga, 2018). We did not observe any increase in overall p‐tau immunolabeling or p‐tau CTE‐like pathology in the subset of the repeat concussive and subconcussive mice we examined and did not further pursue this avenue. Our negative results are not surprising in light of most other repeat TBI studies in mice (see reviews by Bachstetter et al., 2020; Lynch et al., 2016; McAteer, Turner, & Corrigan, 2017; Ojo, Mouzon, & Crawford, 2016). In brief, although some investigators have reported increased p‐tau expression after a single mild TBI event in wild‐type mice (Goldstein et al., 2012; Huber et al., 2013; Iliff et al., 2014; Tagge et al., 2018; Turner et al., 2015) or following repeat mild TBI (Luo et al., 2014; Namjoshi et al., 2013; Petraglia, Plog, Dayawansa, Dashnaw, et al., 2014; Yang et al., 2015), more commonly p‐tau is not increased even after repeat mild TBI (Bolton‐Hall et al., 2016; Chen et al., 2017; Cheng et al., 2019, 2020; Mannix et al., 2013; Mouzon et al., 2014; Xu et al., 2016). The results for mice overexpressing human tau and subjected to multiple mild TBI events are also mixed (increased p‐tau reported by Ojo et al., 2013; Ojo, Mouzon, Algamal, et al., 2016; but not Brody, Benetatos, Bennett, Klemenhagen, & Mac Donald, 2015; Winston et al., 2016; Gangolli et al., 2019). Whether aged h‐tau mice subjected to our repeat blast paradigm would exhibit increased p‐tau expression or not is uncertain. In addition, some repeat TBI studies that examined more than one time point have reported that the p‐tau increase subsides over time (Namjoshi et al., 2013; Petraglia, Plog, Dayawansa, Chen, et al., 2014; Yang et al., 2015), and so it is possible that the mice in our studies might have shown p‐tau pathology at an earlier point in time. Finally, neurofibrillary and/or astrocytic tangles have rarely, if ever, been observed in repeat mild TBI wild‐type mice, and as noted by other investigators (Lynch et al., 2016; McAteer et al., 2017; Ojo, Mouzon, & Crawford, 2016) and to the best of our knowledge, no mouse studies of mild TBI have replicated the perivascular accumulations of p‐tau in neurons and astroglia that are characteristic of CTE.

Several studies have reported increased microglial immunoreactivity in white matter tracts such as the corpus callosum at 6 months or more following repeat injury (Hoogenboom et al., 2019), and there are occasional reports of persistent increases in microglial abundance in cerebral cortex and hippocampus (Chen et al., 2017). However, detailed examination of microglia in gray matter as a long‐term consequence of repeat TBI has been generally lacking. In fact, and in light of the generally negative p‐tau results, several researchers have suggested that the role of neuroinflammation in the long‐term deleterious sequelae following repeat mild TBI deserves more emphasis (Cheng et al., 2020; Lynch et al., 2016; Ojo, Mouzon, & Crawford, 2016).

Finally, persistent cerebrovascular dysfunction in the absence of direct damage to the vasculature by a mild TBI event or events is common in human subjects but largely unexplored in animal models (Sandsmark, Bashir, Wellington, & Diaz‐Arrastia, 2019). One notable exception to this is a study in which mice that had received many impacts (2 a week for 3 months) were shown to exhibit a decrease in cortical blood flow 7 months after the last impact (Lynch et al., 2016). This was accompanied by altered levels of expression for several vascular proteins (laminin, platelet‐derived growth factor receptor β, smooth muscle actin) and poor Barnes maze performance, but no evident BBB breakdown or microglial activation in gray matter. Indeed, BBB compromise seems to be a more frequent occurrence during the first few days after brain injury than at later time points (see review by Rodriguez‐Grande, Ichkova, Lemarchant, & Badaut, 2017) and more common in mice subjected to explosive or shock tube blasts than to impact or acceleration/deceleration TBI.

### 4.7 Human implications

In people with a history of concussive or subconcussive injury, cognitive function often worsens later in life. Although in some cases this is associated with the β‐amyloid deposition pathology of AD or perivascular p‐tau pathology of CTE, memory loss more commonly occurs without AD or CTE pathology (reviewed by Daneshvar et al., 2015; Washington et al., 2016). Genetic background and the number, frequency and severity of the head trauma may determine whether the individual exhibits AD, CTE, or non‐AD/CTE dementia. In the case of CTE, multiple subconcussive events are thought to be pathogenic, even in the absence of any evident concussion. Despite the prevalence of CTE in football and hockey players, CTE by no means occurs in all athletes in high‐contact sports, and so it seems likely genetic predispositions exist for CTE, as is known to be true for AD. For the repeat concussive and subconcussive mice we studied here, we did not observe any notable CTE‐like p‐tau pathology (as discussed above) or any AD‐like β‐amyloid pathology. Instead, our findings suggest that early microglial activation leading to premature microglial aging and dysfunction, as well as vascular dysfunction perhaps driven by the microglial abnormalities, plays an important role in the pathogenic process. According to this point of view, p‐tau or β‐amyloid pathology might be the consequence rather than the cause of a microglia‐driven pathogenic process, although we cannot rule out the possibility they would occur in our model with more repeated injury, some mix of concussive and subconcussive injury, and/or an even longer survival time. Nonetheless, a number of studies have reported persistent microglial activation in atrophied brain regions in post‐mortem specimens and in PET scans of former NFL players with memory decline (Cherry et al., 2016; Coughlin et al., 2017). Moreover, some have suggested that microglial activation leads to the p‐tau accumulation in CTE (Cherry et al., 2016), and a self‐sustaining adverse interaction with nearby blood vessels accounts for the preferential perivascular accumulation of p‐tau (Cherry et al., 2016; McKee et al., 2013). Our findings raise the possibility that progressive microglial de‐activation and/or loss may also or instead contribute to pathogenesis. Note that while we have focused our attention on cognitive deficits and their basis in hippocampal pathology, our repeat TBI mice also showed perseveration and worsening of fear. This aspect of our results is also relevant to humans, given that people with a history of repeat concussive or subconcussive injury frequently exhibit emotional disturbances (Daneshvar et al., 2015; Montenigro et al., 2017), and would be interesting to further explore at a future time.

In any case, our findings raise concerns about repeat subconcussive injury being particularly insidious, echoing the results of behavioral assessments and brain imaging of athletes playing contact sports (Bailes et al., 2013; Montenigro et al., 2017). Importantly, our studies suggest targeting microglia for treatment. If, in fact, persistent microglial activation leading to microglial de‐activation is pathogenic, then treatments to quell microglial activation or to replenish microglia may be therapeutic. Our own work shows that modulating microglia away from the M1 state toward the M2 state, by treating with CB2 inverse agonists, reduces functional deficits and neuron loss following TBI (Bu et al., 2016; Guley et al., 2019; Honig et al., 2019; Liu et al., 2017; Reiner et al., 2015). Alternatively or in addition, microglial depletion using inhibitors of colony‐stimulating factor 1 receptor, followed by naturally occurring microglial replenishment, may serve as an effective therapeutic strategy (e.g., Elmore et al., 2018).

## CONFLICT OF INTEREST

None of the authors has any interest or relationship that might be perceived as influencing their objectivity.

## AUTHOR CONTRIBUTIONS

Marcia G. Honig and Anton Reiner planned the studies, analyzed the data, and wrote the paper. Conor C. Dorian analyzed the data and edited the paper. John D. Worthen, Anthony C. Micetich, Isabelle A. Mulder, Katelyn B. Sanchez, and William F. Pierce analyzed the data. Nobel A. Del Mar performed the TBI and behavioral studies.

## Data Availability

The data used and/or analyzed during the current study are available from the corresponding author on reasonable request.

## References

[ejn14711-bib-0001] Agoston, D. V. (2017). Modeling the long‐term consequences of repeated blast‐induced mild traumatic brain injuries. Journal of Neurotrauma, 34, S44–S52. 28937952 10.1089/neu.2017.5317 PMC5610388

[ejn14711-bib-0002] Askew, K., Li, K., Olmos‐Alonso, A., Garcia‐Moreno, F., Liang, Y., Richardson, P., … Gomez‐Nicola, D. (2017). Coupled proliferation and apoptosis maintain the rapid turnover of microglia in the adult brain. Cell Reports, 18, 391–405. 28076784 10.1016/j.celrep.2016.12.041 PMC5263237

[ejn14711-bib-0003] Azur, M. J., Stuart, E. A., Frangakis, C., & Leaf, P. J. (2011). Multiple imputation by chained equations: What is it and how does it work? International Journal of Methods in Psychiatric Research, 20, 40–49. 21499542 10.1002/mpr.329 PMC3074241

[ejn14711-bib-0004] Bachstetter, A. D., Morganti, J. M., Bodnar, C. N., Webster, S. J., Higgins, E. K., Roberts, K. N., … Abisambra, J. F. (2020). The effects of mild closed head injuries on tauopathy and cognitive deficits in rodents: Primary results in wild type and rTg4510 mice, and a systematic review. Experimental Neurology, 326, 113180. 31930992 10.1016/j.expneurol.2020.113180 PMC7373372

[ejn14711-bib-0005] Bachstetter, A. D., Van Eldik, L. J., Schmitt, F. A., Neltner, J. H., Ighodaro, E. T., Webster, S. J., … Nelson, P. T. (2015). Disease‐related microglia heterogeneity in the hippocampus of Alzheimer's disease, dementia with Lewy bodies, and hippocampal sclerosis of aging. Acta Neuropathologica Communications, 3, 32. 26001591 10.1186/s40478-015-0209-z PMC4489160

[ejn14711-bib-0006] Bailes, J. E., Petraglia, A. L., Omalu, B. I., Nauman, E., & Talavage, T. (2013). Role of subconcussion in repetitive mild traumatic brain injury. Journal of Neurosurgery, 119, 1235–1245. 23971952 10.3171/2013.7.JNS121822

[ejn14711-bib-0007] Bazarian, J. J., Donnelly, K., Peterson, D. R., Warner, G. C., Zhu, T., & Zhong, J. (2013). The relation between posttraumatic stress disorder and mild traumatic brain injury acquired during operations Enduring Freedom and Iraqi Freedom. The Journal of Head Trauma Rehabilitation, 28, 1–12. 22647965 10.1097/HTR.0b013e318256d3d3

[ejn14711-bib-0008] Beckwith, J. G., Zhao, W., Ji, S., Ajamil, A. G., Bolander, R. P., Chu, J. J., … Greenwald, R. M. (2018). Estimated brain tissue response following impacts associated with and without diagnosed concussion. Annals of Biomedical Engineering, 46(6), 819–830. 29470745 10.1007/s10439-018-1999-5 PMC5935583

[ejn14711-bib-0009] Belanger, H. G., Vanderploeg, R. D., & McAllister, T. (2016). Subconcussive blows to the head: A formative review of short‐term clinical outcomes. Journal of Head Trauma Rehabilitation, 31, 159–166. 10.1097/HTR.0000000000000138 25931186

[ejn14711-bib-0010] Beynon, S. B., & Walker, F. R. (2012). Microglial activation in the injured and healthy brain: What are we really talking about? Practical and theoretical issues associated with the measurement of changes in microglial morphology. Neuroscience, 225, 162–171. 22824429 10.1016/j.neuroscience.2012.07.029

[ejn14711-bib-0011] Biber, K., Owens, T., & Boddeke, E. (2014). What is microglia neurotoxicity (Not)? Glia, 62, 841–854. 24590682 10.1002/glia.22654

[ejn14711-bib-0012] Blennow, K., Brody, D. L., Kochanek, P. M., Levin, H., McKee, A., Ribbers, G. M., … Zetterberg, H. (2016). Traumatic brain injuries. Nature Reviews Disease Primers, 2, 16084. 10.1038/nrdp.2016.84 27853132

[ejn14711-bib-0013] Bolton‐Hall, A. N., Hubbard, W. B., & Saatman, K. E. (2019). Experimental designs for repeated mild traumatic brain injury: Challenges and considerations. Journal of Neurotrauma, 36, 1203–1221. 30351225 10.1089/neu.2018.6096 PMC6479246

[ejn14711-bib-0014] Bolton‐Hall, A. N., Joseph, B., Brelsfoard, J. M., & Saatman, K. E. (2016). Repeated closed head injury in mice results in sustained motor and memory deficits and chronic cellular changes. PLoS ONE, 11(7), e0159442. 27427961 10.1371/journal.pone.0159442 PMC4948770

[ejn14711-bib-0015] Bregy, A., Nixon, R., Lotocki, G., Alonso, O. F., Atkins, C. M., Rsoulfas, P., … Dietrich, W. D. (2012). Posttraumatic hypothermia increases doublecortin expressing neurons in the dentate gyrus after traumatic brain injury in the rat. Experimental Neurology, 233, 821–828. 22197046 10.1016/j.expneurol.2011.12.008 PMC3272120

[ejn14711-bib-0016] Brody, D. L., Benetatos, J., Bennett, R. E., Klemenhagen, K. C., & Mac Donald, C. L. (2015). The pathophysiology of repetitive concussive traumatic brain injury in experimental models; new developments and open questions. Molecular and Cellular Neurosciences, 66(Pt B), 91–98. 25684677 10.1016/j.mcn.2015.02.005 PMC4461503

[ejn14711-bib-0017] Brown, G. C., & Vilalta, A. (2015). How microglia kill neurons. Brain Research, 1628(Pt B), 288–297. 26341532 10.1016/j.brainres.2015.08.031

[ejn14711-bib-0018] Brown, J. P., Couillard‐Després, S., Cooper‐Kuhn, C. M., Winkler, J., Aigner, L., & Kuhn, H. G. (2003). Transient expression of doublecortin during adult neurogenesis. The Journal of Comparative Neurology, 467, 1–10. 14574675 10.1002/cne.10874

[ejn14711-bib-0019] Browne, K. D., Chen, X. H., Meaney, D. F., & Smith, D. H. (2011). Mild traumatic brain injury and diffuse axonal injury in swine. Journal of Neurotrauma, 28(9), 1747–1755. 21740133 10.1089/neu.2011.1913 PMC3172883

[ejn14711-bib-0020] Bu, W., Ren, H., Deng, Y., Del Mar, N., Guley, N., Moore, B., … Reiner, A. (2016). Mild traumatic brain injury produces neuron loss that can be rescued by modulating microglial activation using a CB2 receptor inverse agonist. Frontiers in Neuroscience, 10, 449. 27766068 10.3389/fnins.2016.00449 PMC5052277

[ejn14711-bib-0021] Buckley, E. M., Miller, B. F., Golinski, J. M., Sadeghian, H., McAllister, L. M., Vangel, M., … Whalen, M. J. (2015). Decreased microvascular cerebral blood flow assessed by diffuse correlation spectroscopy after repetitive concussions in mice. Journal of Cerebral Blood Flow & Metabolism, 35, 1995–2000. 26154866 10.1038/jcbfm.2015.161 PMC4671120

[ejn14711-bib-0022] Cao, T., Thomas, T. C., Ziebell, J. M., Pauly, J. R., & Lifshitz, J. (2012). Morphological and genetic activation of microglia after diffuse traumatic brain injury in the rat. Neuroscience, 225, 65–75. 22960311 10.1016/j.neuroscience.2012.08.058 PMC3489473

[ejn14711-bib-0023] Chadehumbe, M. A. (2016). Neurologic care in concussive and post‐concussive encephalopathy. Current Problems in Pediatric and Adolescent Health Care, 46, 52–57. 26688461 10.1016/j.cppeds.2015.11.004

[ejn14711-bib-0024] Chandra, N., & Sundaramurthy, A. (2015). Acute pathophysiology of blast injury‐from biomechanics to experiments and computations implications on head and polytrauma. In F. H. Kobeissy (Ed.), Brain neurotrauma: Molecular, neuropsychological, and rehabilitation aspects (pp. 199–258). Boca Raton, FL: CRC Press/ Taylor & Francis, Chapter 18.

[ejn14711-bib-0025] Chen, H., Desai, A., & Kim, H. Y. (2017). Repetitive closed‐head impact model of engineered rotational acceleration induces long‐term cognitive impairments with persistent astrogliosis and microgliosis in mice. Journal of Neurotrauma, 34(14), 2291–2302. 28288551 10.1089/neu.2016.4870 PMC5510798

[ejn14711-bib-0026] Chen, K., Gu, H., Zhu, L., & Feng, D. F. (2020) A new model of repetitive traumatic brain injury in mice. Frontiers in Neuroscience, 13, 1417. 32038131 10.3389/fnins.2019.01417 PMC6985558

[ejn14711-bib-0027] Chen, Y., Huang, W., & Constantini, S. (2013). The differences between blast‐induced and sports‐related brain injuries. Frontiers in Neurology, 14(4), 119. 10.3389/fneur.2013.00119 PMC3743039 23966976

[ejn14711-bib-0028] Cheng, H., Deaton, L. M., Qiu, M., Ha, S., Pacoma, R., Lao, J., … Schumacher, A. M. (2020). Tau overexpression exacerbates neuropathology after repeated mild head impacts in male mice. Neurobiology of Diseases, 134, 104683. 10.1016/j.nbd.2019.104683 31765727

[ejn14711-bib-0029] Cheng, W. H., Martens, K. M., Bashir, A., Cheung, H., Stukas, S., Gibbs, E., … Wellington, C. L. (2019). CHIMERA repetitive mild traumatic brain injury induces chronic behavioural and neuropathological phenotypes in wild‐type and APP/PS1 mice. Alzheimer's Research & Therapy, 11(1), 6. 10.1186/s13195-018-0461-0 PMC6330571 30636629

[ejn14711-bib-0030] Cherry, J. D., Olschowka, J. A., & O'Banion, M. K. (2014). Neuroinflammation and M2 microglia: The good, the bad, and the inflamed. Journal of Neuroinflammation, 11, 98. 24889886 10.1186/1742-2094-11-98 PMC4060849

[ejn14711-bib-0031] Cherry, J. D., Tripodis, Y., Alvarez, V. E., Huber, B., Kiernan, P. T., Daneshvar, D. H., … Stein, T. D. (2016). Microglial neuroinflammation contributes to tau accumulation in chronic traumatic encephalopathy. Acta Neuropathologica Communications, 4, 112. 27793189 10.1186/s40478-016-0382-8 PMC5084333

[ejn14711-bib-0032] Cho, H. J., Sajja, V. S., Vandevord, P. J., & Lee, Y. W. (2013). Blast induces oxidative stress, inflammation, neuronal loss and subsequent short‐term memory impairment in rats. Neuroscience, 253, 9–20. 23999126 10.1016/j.neuroscience.2013.08.037

[ejn14711-bib-0033] Christian, K. M., Song, H., & Ming, G. L. (2014). Functions and dysfunctions of adult hippocampal neurogenesis. Annual Review of Neuroscience, 37, 243–262. 10.1146/annurev-neuro-071013-014134 PMC5531058 24905596

[ejn14711-bib-0034] Coughlin, J. M., Wang, Y., Minn, I., Bienko, N., Ambinder, E. B., Xu, X., … Pomper, M. G. (2017). Imaging of glial cell activation and white matter integrity in brains of active and recently retired National Football League players. JAMA Neurology, 74, 67–74. 27893897 10.1001/jamaneurol.2016.3764 PMC5504689

[ejn14711-bib-0035] Coughlin, J. M., Wang, Y., Munro, C. A., Ma, S., Yue, C., Chen, S., … Pomper, M. G. (2015). Neuroinflammation and brain atrophy in former NFL players: An in vivo multimodal imaging pilot study. Neurobiology of Diseases, 74, 58–65. 10.1016/j.nbd.2014.10.019 PMC4411636 25447235

[ejn14711-bib-0036] Creed, J. A., DiLeonardi, A. M., Fox, D. P., Tessler, A. R., & Raghupathi, R. (2011). Concussive brain trauma in the mouse results in acute cognitive deficits and sustained impairment of axonal function. Journal of Neurotrauma, 28, 547–563. 21299360 10.1089/neu.2010.1729 PMC3070143

[ejn14711-bib-0037] Daneshvar, D. H., Goldstein, L. E., Kiernan, P. T., Stein, T. D., & McKee, A. C. (2015). Post‐traumatic neurodegeneration and chronic traumatic encephalopathy. Molecular and Cellular Neurosciences, 66, 81–90. 25758552 10.1016/j.mcn.2015.03.007

[ejn14711-bib-0038] De Lucia, C., Rinchon, A., Olmos‐Alonso, A., Riecken, K., Fehse, B., Boche, D., … Gomez‐Nicola, D. (2016). Microglia regulate hippocampal neurogenesis during chronic neurodegeneration. Brain Behavior & Immunity, 55, 179–190. 10.1016/j.bbi.2015.11.001 PMC4907582 26541819

[ejn14711-bib-0039] Deczkowska, A., Amit, I., & Schwartz, M. (2018). Microglial immune checkpoint mechanisms. Nature Neuroscience, 21, 779–786. 29735982 10.1038/s41593-018-0145-x

[ejn14711-bib-0040] Del Mar, N., von Buttlar, X., Yu, A. S., Guley, N. H., Reiner, A., & Honig, M. G. (2015). A novel closed‐body model of spinal cord injury caused by high‐pressure air blasts produces extensive axonal injury and motor impairments. Experimental Neurology, 271, 53–71. 25957630 10.1016/j.expneurol.2015.04.023 PMC4586366

[ejn14711-bib-0041] Donat, C. K., Scott, G., Gentleman, S. M., & Sastre, M. (2017). Microglial activation in traumatic brain injury. Frontiers in Aging Neuroscience, 9, 208. 28701948 10.3389/fnagi.2017.00208 PMC5487478

[ejn14711-bib-0042] Dubbelaar, M. L., Kracht, L., Eggen, B. J. L., & Boddeke, E. W. G. M. (2018). The kaleidoscope of microglial phenotypes. Frontiers in Immunology, 9, 1753. 30108586 10.3389/fimmu.2018.01753 PMC6079257

[ejn14711-bib-0043] Dudchenko, P. A. (2004). An overview of the tasks used to test working memory in rodents. Neuroscience and Biobehavioral Reviews, 28, 699–709. 15555679 10.1016/j.neubiorev.2004.09.002

[ejn14711-bib-0044] Dudvarski Stankovic, N., Teodorczyk, M., Ploen, R., Zipp, F., & Schmidt, M. H. H. (2016). Microglia‐blood vessel interactions: A double‐edged sword in brain pathologies. Acta Neuropathologica, 131, 347–363. 26711460 10.1007/s00401-015-1524-y

[ejn14711-bib-0045] Effgen, G. B., Ong, T., Nammalwar, S., Ortuño, A. I., Meaney, D. F., Bass, C. R., & Morrison, B. (2016). Primary blast exposure increases hippocampal vulnerability to subsequent exposure: Reducing long‐term potentiation. Journal of Neurotrauma, 33, 1901–1912. 26699926 10.1089/neu.2015.4327 PMC6445278

[ejn14711-bib-0046] Elder, G. A., Gama Sosa, M. A., De Gasperi, R., Stone, J. R., Dickstein, D. L., Haghighi, F., … Ahlers, S. T. (2015). Vascular and inflammatory factors in the pathophysiology of blast‐induced brain injury. Frontiers in Neurology, 6, 48. 25852632 10.3389/fneur.2015.00048 PMC4360816

[ejn14711-bib-0047] Elmore, M. R. P., Hohsfield, L. A., Kramár, E. A., Soreq, L., Lee, R. J., Pham, S. T., … Green, K. N. (2018). Replacement of microglia in the aged brain reverses cognitive, synaptic, and neuronal deficits in mice. Aging Cell, 17(6), e12832. 30276955 10.1111/acel.12832 PMC6260908

[ejn14711-bib-0048] Esopenko, C., & Levine, B. (2017). Autobiographical memory and structural brain changes in chronic phase TBI. Cortex, 89, 1–10. 28189664 10.1016/j.cortex.2017.01.007

[ejn14711-bib-0049] Faden, A. I., & Loane, D. J. (2015). Chronic neurodegeneration after traumatic brain injury: Alzheimer disease, chronic traumatic encephalopathy, or persistent neuroinflammation? Neurotherapeutics: the Journal of the American Society for Experimental NeuroTherapeutics, 12, 143–150. 25421001 10.1007/s13311-014-0319-5 PMC4322076

[ejn14711-bib-0050] Fanselow, M. S., & Dong, H. W. (2010). Are the dorsal and ventral hippocampus functionally distinct structures? Neuron, 65, 7–19. 20152109 10.1016/j.neuron.2009.11.031 PMC2822727

[ejn14711-bib-0051] Faul, M., Xu, L., Wald, M. M., & Coronado, V. G. (2010). Traumatic brain injury in the United States: Emergency Department visits, hospitalizations, and deaths, 2002–2006. Atlanta, GA: Centers for Disease Control and Prevention.

[ejn14711-bib-0052] Ferguson, S., Mouzon, B. C., Paris, D., Aponte, D., Abdullah, L., Stewart, W., … Crawford, F. C. (2017). Acute or delayed treatment with anatabine improves spatial memory and reduces pathological sequelae at chronic timepoints after repetitive mild TBI. Journal of Neurotrauma, 34, 1676–1691. 27889957 10.1089/neu.2016.4636 PMC5749608

[ejn14711-bib-0053] Fernández‐Arjona, M. D. M., Grondona, J. M., Granados‐Durán, P., Fernández‐Llebrez, P., & López‐Ávalos, M. D. (2017). Microglia morphological categorization in a rat model of neuroinflammation by hierarchical cluster and principal components analysis. Frontiers in Cellular Neuroscience, 11, 235. 28848398 10.3389/fncel.2017.00235 PMC5550745

[ejn14711-bib-0054] Frank, M. G., Fonken, L. K., Watkins, L. R., & Maier, S. F. (2019). Microglia: Neuroimmune‐sensors of stress. Seminars in Cell & Developmental Biology, 94, 176–185. 30638704 10.1016/j.semcdb.2019.01.001 PMC6614020

[ejn14711-bib-0055] Friess, S. H., Ichord, R. N., Ralston, J., Ryall, K., Helfaer, M. A., Smith, C., & Margulies, S. S. (2009). Repeated traumatic brain injury affects composite cognitive function in piglets. Journal of Neurotrauma, 26, 1111–1121. 19275468 10.1089/neu.2008.0845 PMC2848948

[ejn14711-bib-0056] Gangolli, M., Benetatos, J., Esparza, T. J., Fountain, E. M., Seneviratne, S., & Brody, D. L. (2019). Repetitive concussive and subconcussive injury in a human tau mouse model results in chronic cognitive dysfunction and disruption of white matter tracts, but not tau pathology. Journal of Neurotrauma, 36, 735–755. 30136628 10.1089/neu.2018.5700 PMC6387572

[ejn14711-bib-0057] Glass, C. K., Saljo, K., Winner, B., Marchetto, M. C., & Gage, F. H. (2010). Mechanisms underlying inflammation in neurodegeneration. Cell, 140, 918–934. 20303880 10.1016/j.cell.2010.02.016 PMC2873093

[ejn14711-bib-0058] Go, M., Kou, J., Lim, J. E., Yang, J., & Fukuchi, K. I. (2016). Microglial response to LPS increases in wild‐type mice during aging but diminishes in an Alzheimer's mouse model: Implication of TLR4 signaling in disease progression. Biochemical and Biophysical Research Communications, 479, 331–337. 27641666 10.1016/j.bbrc.2016.09.073 PMC5048480

[ejn14711-bib-0059] Goldstein, L. E., Fisher, A. M., Tagge, C. A., Zhang, X. L., Velisek, L., Sullivan, J. A., … McKee, A. C. (2012). Chronic traumatic encephalopathy in blast‐exposed military veterans and a blast neurotrauma mouse model. Science Translational Medicine, 4(134), 134ra60. 10.1126/scitranslmed.3003716 PMC3739428 22593173

[ejn14711-bib-0060] Gonçalves, J. T., Schafer, S. T., & Gage, F. H. (2016). Adult neurogenesis in the hippocampus: From stem cells to behavior. Cell, 167, 897–914. 27814520 10.1016/j.cell.2016.10.021

[ejn14711-bib-0061] Grabert, K., Michoel, T., Karavolos, M. H., Clohisey, S., Baillie, J. K., Stevens, M. P., … McColl, B. W. (2016). Microglial brain region‐dependent diversity and selective regional sensitivities to aging. Nature Neuroscience, 19, 504–516. 26780511 10.1038/nn.4222 PMC4768346

[ejn14711-bib-0062] Guley, N. M., Del Mar, N. A., Ragsdale, T., Li, C., Perry, A. M., Moore, B. M., … Reiner, A. (2019). Amelioration of visual deficits and visual system pathology after mild TBI with the cannabinoid type‐2 receptor inverse agonist SMM‐189. Experimental Eye Research, 182, 109–124. 30922891 10.1016/j.exer.2019.03.013 PMC6504571

[ejn14711-bib-0063] Guley, N. G., Rogers, J. T., Del Mar, N. A., Deng, Y., Islam, R. M., D'Surney, L., … Reiner, A. (2016). A novel closed‐head model of mild traumatic brain injury using focal primary overpressure blast to the cranium in mice. Journal of Neurotrauma, 33, 403–422. 26414413 10.1089/neu.2015.3886 PMC4761824

[ejn14711-bib-0064] Hefendehl, J. K., Neher, J. J., Sühs, R. B., Kohsaka, S., Skodras, A., & Jucker, M. (2014). Homeostatic and injury‐induced microglia behavior in the aging brain. Aging Cell, 13, 60–69. 23953759 10.1111/acel.12149 PMC4326865

[ejn14711-bib-0065] Heldt, S. A., Elberger, A. J., Deng, Y., Guley, N. H., Del Mar, N., Rogers, J., … Reiner, A. (2014). A novel closed‐head model of mild TBI caused by primary overpressure blast to the cranium produces sustained emotional deficits in mice. Frontiers in Neurology, 5, 2. 24478749 10.3389/fneur.2014.00002 PMC3898331

[ejn14711-bib-0066] Hernandez, A., Tan, C., Plattner, F., Logsdon, A. F., Pozo, K., Yousuf, M. A., … Bibb, J. A. (2018). Exposure to mild blast forces induces neuropathological effects, neurophysiological deficits and biochemical changes. Molecular Brain, 11(1), 64. 30409147 10.1186/s13041-018-0408-1 PMC6225689

[ejn14711-bib-0067] Hickman, S., Izzy, S., Sen, P., Morsett, L., & El Khoury, J. (2018). Microglia in neurodegeneration. Nature Neuroscience, 21, 1359–1369. 30258234 10.1038/s41593-018-0242-x PMC6817969

[ejn14711-bib-0068] Hickman, S. E., Kingery, N. D., Ohsumi, T. K., Borowsky, M. L., Wang, L. C., Means, T. K., & El Khoury, J. (2013). The microglial sensome revealed by direct RNA sequencing. Nature Neuroscience, 16, 1896–1905. 24162652 10.1038/nn.3554 PMC3840123

[ejn14711-bib-0069] Hong, S., Dissing‐Olesen, L., & Stevens, B. (2016). New insights on the role of microglia in synaptic pruning in health and disease. Current Opinion in Neurobiology, 36, 128–134. 26745839 10.1016/j.conb.2015.12.004 PMC5479435

[ejn14711-bib-0070] Honig, M. G., Del Mar, N., Henderson, D. L., Ragsdale, T. D., Doty, J. B., Driver, J. H., … Reiner, A. (2019). Amelioration of visual deficits and visual system pathology after mild TBI via the cannabinoid type‐2 receptor inverse agonism of raloxifene. Experimental Neurology, 322, 13063. 10.1016/j.expneurol.2019.113063 31518568

[ejn14711-bib-0071] Hoogenboom, W. S., Branch, C. A., & Lipton, M. L. (2019). Animal models of closed‐skull, repetitive mild traumatic brain injury. Pharmacology & Therapeutics, 198, 109–122. 30822463 10.1016/j.pharmthera.2019.02.016 PMC6536340

[ejn14711-bib-0072] Hoskison, M. M., Moore, A. N., Hu, B., Orsi, S., Kobori, N., & Dash, P. K. (2009). Persistent working memory dysfunction following traumatic brain injury: Evidence for a time‐dependent mechanism. Neuroscience, 159, 483–491. 19167462 10.1016/j.neuroscience.2008.12.050 PMC4264540

[ejn14711-bib-0073] Huber, B. R., Meabon, J. S., Martin, T. J., Mourad, P. D., Bennett, R., Kraemer, B. C., … Cook, D. G. (2013). Blast exposure causes early and persistent aberrant phospho‐ and cleaved‐tau expression in a murine model of mild blast‐induced traumatic brain injury. Journal of Alzheimer's Disease, 37, 309–323. 10.3233/JAD-130182 PMC4126588 23948882

[ejn14711-bib-0074] Hylin, M. J., Orsi, S. A., Rozas, N. S., Hill, J. L., Zhao, J., Redell, J. B., … Dash, P. K. (2013). Repeated mild closed head injury impairs short‐term visuospatial memory and complex learning. Journal of Neurotrauma, 30(9), 716–726. 23489238 10.1089/neu.2012.2717

[ejn14711-bib-0075] Iliff, J. J., Chen, M. J., Plog, B. A., Zeppenfeld, D. M., Soltero, M., Yang, L., … Nedergaard, M. (2014). Impairment of glymphatic pathway function promotes tau pathology after traumatic brain injury. The Journal of Neuroscience, 34, 16180–16193. 25471560 10.1523/JNEUROSCI.3020-14.2014 PMC4252540

[ejn14711-bib-0076] Johnson, V. E., Stewart, W., & Smith, D. H. (2013). Axonal pathology in traumatic brain injury. Experimental Neurology, 246, 35–43. 22285252 10.1016/j.expneurol.2012.01.013 PMC3979341

[ejn14711-bib-0077] Kane, M. J., Angoa‐Pérez, M., Briggs, D. I., Viano, D. C., Kreipke, C. W., & Kuhn, D. M. (2012). A mouse model of human repetitive mild traumatic brain i7njury. Journal of Neuroscience Methods, 203, 41–49. 21930157 10.1016/j.jneumeth.2011.09.003 PMC3221913

[ejn14711-bib-0078] Karr, J. E., Areshenkoff, C. N., Duggan, E. C., & Garcia‐Barrera, M. A. (2014). Blast‐related mild traumatic brain injury: A Bayesian random‐effects meta‐analysis on the cognitive outcomes of concussion among military personnel. Neuropsychology Review, 24, 428–444. 25253505 10.1007/s11065-014-9271-8

[ejn14711-bib-0079] Kasahara, M., Menon, D. K., Salmond, C. H., Outtrim, J. G., Tavares, J. V., Carpenter, T. A., … Stamatakis, E. A. (2011). Traumatic brain injury alters the functional brain network mediating working memory. Brain Injury, 25, 1170–1187. 21932994 10.3109/02699052.2011.608210

[ejn14711-bib-0080] Keren‐Shaul, H., Spinrad, A., Weiner, A., Matcovitch‐Natan, O., Dvir‐Szternfeld, R., Ulland, T. K., … Amit, I. (2017). A unique microglia type associated with restricting development of Alzheimer's disease. Cell, 169, 1276–1290. 28602351 10.1016/j.cell.2017.05.018

[ejn14711-bib-0081] Khan, A. M., Babcock, A. A., Saeed, H., Myhre, C. L., Kassem, M., & Finsen, B. (2015). Telomere dysfunction reduces microglial numbers without fully inducing an aging phenotype. Neurobiology of Aging, 36, 2164–2175. 25892207 10.1016/j.neurobiolaging.2015.03.008

[ejn14711-bib-0082] Kimura, T., Sharma, G., Ishiguro, K., & Hisanaga, S. (2018). Phospho‐tau bar code: Analysis of phosphoisotypes of tau and its application to tauopathy. Frontiers in Neuroscience, 12, 44. 29467609 10.3389/fnins.2018.00044 PMC5808175

[ejn14711-bib-0083] Koliatsos, V. E., Cernak, I., Xu, L., Song, Y., Savonenko, A., Crain, B. J., … Lee, D. (2011). A mouse model of blast injury to brain: Initial pathological, neuropathological, and behavioral characterization. Journal of Neuropathology and Experimental Neurology, 70, 399–416. 21487304 10.1097/NEN.0b013e3182189f06

[ejn14711-bib-0084] Krasemann, S., Madore, C., Cialic, R., Baufeld, C., Calcagno, N., El Fatimy, R., … Butovsky, O. (2017). The TREM2‐APOE pathway drives the transcriptional phenotype of dysfunctional microglia in neurodegenerative diseases. Immunity, 47, 566–581. 28930663 10.1016/j.immuni.2017.08.008 PMC5719893

[ejn14711-bib-0085] Kreisel, T., Frank, M. G., Licht, T., Reshef, R., Ben‐Menachem‐Zidon, O., Baratta, M. V., … Yirmiya, R. (2014). Dynamic microglial alterations underlie stress‐induced depressive‐like behavior and suppressed neurogenesis. Molecular Psychiatry, 19, 699–709. 24342992 10.1038/mp.2013.155

[ejn14711-bib-0086] Kumar, A., Alvarez‐Croda, D. M., Stoica, B. A., Faden, A. I., & Loane, D. J. (2016). Microglial/macrophage polarization dynamics following traumatic brain injury. Journal of Neurotrauma, 33, 1732–1750. 26486881 10.1089/neu.2015.4268 PMC5065034

[ejn14711-bib-0087] Kumar, A., & Loane, D. J. (2012). Neuroinflammation after traumatic brain injury: Opportunities for therapeutic intervention. Brain, Behavior, and Immunity, 26, 1191–1201. 10.1016/j.bbi.2012.06.008 22728326

[ejn14711-bib-0088] Kunimoto, S., Nakamura, S., Wada, K., & Inoue, T. (2010). Chronic stress‐mutated presenilin 1 gene interaction perturbs neurogenesis and accelerates neurodegeneration. Experimental Neurology, 221, 175–185. 19896484 10.1016/j.expneurol.2009.10.020

[ejn14711-bib-0089] Laurer, H. L., Bareyre, F. M., Lee, V. M., Trojanowski, J. Q., Longhi, L., Hoover, R., … McIntosh, T. K. (2001). Mild head injury increasing the brain’s vulnerability to a second concussive impact. Journal of Neurosurgery, 95, 859–870. 11702878 10.3171/jns.2001.95.5.0859

[ejn14711-bib-0090] Lertkiatmongkol, P., Liao, D., Mei, H., Hu, Y., & Newman, P. J. (2016). Endothelial functions of platelet/endothelial cell adhesion molecule‐1 (CD31). Current Opinion in Hematology, 23, 253–259. 27055047 10.1097/MOH.0000000000000239 PMC4986701

[ejn14711-bib-0091] Liu, V. W., & Huang, P. L. (2008). Cardiovascular roles of nitric oxide: A review of insights from nitric oxide synthase gene disrupted mice. Cardiovascular Research, 77, 19–29. 17658499 10.1016/j.cardiores.2007.06.024 PMC2731989

[ejn14711-bib-0092] Liu, Y., McAfee, S. S., Guley, N. M., Del Mar, N., Bu, W., Heldt, S. A., … Heck, D. H. (2017) Abnormalities in dynamic brain activity caused by mild traumatic brain injury are partially rescued by the cannabinoid type‐2 receptor inverse agonist SMM‐189. Eneuro, 18(4), 4. 10.1523/ENEURO.0387-16.2017 PMC5562300 28828401

[ejn14711-bib-0093] Loane, D. J., & Kumar, A. (2016). Microglia in the TBI brain: The good, the bad, and the dysregulated. Experimental Neurology, 275, 316–327. 26342753 10.1016/j.expneurol.2015.08.018 PMC4689601

[ejn14711-bib-0094] Loane, D. J., Kumar, A., Stoica, B. A., Cabatbat, R., & Faden, A. I. (2014). Progressive neurodegeneration after experimental brain trauma: Association with chronic microglial activation. Journal of Neuropathology & Experimental Neurology, 73, 14–19. 24335533 10.1097/NEN.0000000000000021 PMC4267248

[ejn14711-bib-0095] Longhi, L., Saatman, K. E., Fujimoto, S., Raghupathi, R., Meaney, D. F., Davis, J., … McIntosh, T. K. (2005). Temporal window of vulnerability to repetitive experimental concussive brain injury. Neurosurgery, 56, 364–374. 15670384 10.1227/01.neu.0000149008.73513.44

[ejn14711-bib-0096] Luo, J., Nguyen, A., Villeda, S., Zhang, H., Ding, Z., Lindsey, D., … Wyss‐Coray, T. (2014). Long‐term cognitive impairments and pathological alterations in a mouse model of repetitive mild traumatic brain injury. Frontiers in Neurology, 5, 12. 24550885 10.3389/fneur.2014.00012 PMC3912443

[ejn14711-bib-0097] Luo, X. G., Ding, J. Q., & Chen, S. D. (2010). Microglia in the aging brain: Relevance to neurodegeneration. Molecular Neurodegeneration, 5, 12. 20334662 10.1186/1750-1326-5-12 PMC2852379

[ejn14711-bib-0098] Lynch, C. E., Crynen, G., Ferguson, S., Mouzon, B., Paris, D., Ojo, J., … Bachmeier, C. (2016). Chronic cerebrovascular abnormalities in a mouse model of repetitive mild traumatic brain injury. Brain Injury, 30, 1414–1427. 27834539 10.1080/02699052.2016.1219060

[ejn14711-bib-0099] Madhukar, A., & Ostoja‐Starzewski, M. (2019). Finite element methods in human head impact simulations: A review. Annals of Biomedical Engineering, 47(9), 1832–1854. 30693442 10.1007/s10439-019-02205-4

[ejn14711-bib-0100] Mannix, R., Meehan, W. P., Mandeville, J., Grant, P. E., Gray, T., Berglass, J., … Whalen, M. (2013). Clinical correlates in an experimental model of repetitive mild brain injury. Annals of Neurology, 74, 65–75. 23922306 10.1002/ana.23858 PMC6312716

[ejn14711-bib-0101] Mathys, H., Adaikkan, C., Gao, F., Young, J. Z., Manet, E., Hemberg, M., … Tsai, L. H. (2017). Temporal tracking of microglia activation in neurodegeneration at single‐cell resolution. Cell Reports, 21, 366–380. 29020624 10.1016/j.celrep.2017.09.039 PMC5642107

[ejn14711-bib-0102] McAteer, K. M., Turner, R. J., & Corrigan, F. (2017). Animal models of chronic traumatic encephalopathy. Concussion, 2(2), CNC32. 30202573 10.2217/cnc-2016-0031 PMC6093772

[ejn14711-bib-0103] McCrory, P., Meeuwisse, W., Aubry, M., Cantu, B., Dvorak, J., Echemendia, R., … Turner, M. (2013) Consensus statement on concussion in sport: the 4th International Conference on Concussion in Sport held in Zurich, November 2012. British Journal of Sports Medicine, 47(5), 250–258. 10.1136/bjsports-2013-092313 23479479

[ejn14711-bib-0104] McKee, A. C., Cairns, N. J., Dickson, D. W., Folkerth, R. D., Keene, C. D., Litvan, I., … TBI/CTE group (2016). The first NINDS/NIBIB consensus meeting to define neuropathological criteria for the diagnosis of chronic traumatic encephalopathy. Acta Neuropathologica, 131, 75–86. 26667418 10.1007/s00401-015-1515-z PMC4698281

[ejn14711-bib-0105] McKee, A. C., Stein, T. D., Nowinski, C. J., Stern, R. A., Daneshvar, D. H., Alvarez, V. E., … Cantu, R. C. (2013). The spectrum of disease in chronic traumatic encephalopathy. Brain, 136, 43–64. 23208308 10.1093/brain/aws307 PMC3624697

[ejn14711-bib-0106] Meehan, W. P. 3rd, Zhang, J., Mannix, R., & Whalen, M. J. (2012). Increasing recovery time between injuries improves cognitive outcome after repetitive mild concussive brain injuries in mice. Neurosurgery, 71, 885–8991. 22743360 10.1227/NEU.0b013e318265a439 PMC5815628

[ejn14711-bib-0107] Mohler, E. G., Baker, P. M., Gannon, K. S., Jones, S. S., Shacham, S., Sweeney, J. A., & Ragozzino, M. E. (2007). The effects of PRX‐07034, a novel 5‐HT6 antagonist, on cognitive flexibility and working memory in rats. Psychopharmacology (Berl), 220, 687–696. 10.1007/s00213-011-2518-7 PMC3636983 21989804

[ejn14711-bib-0108] Montenigro, P. H., Alosco, M. L., Martin, B. M., Daneshvar, D. H., Mez, J., Chaisson, C. E., … Tripodis, Y. (2017). Cumulative head impact exposure predicts later‐life depression, apathy, executive dysfunction, and cognitive impairment in former high school and college football players. Journal of Neurotrauma, 34, 328–340. 27029716 10.1089/neu.2016.4413 PMC5220530

[ejn14711-bib-0109] Mosher, K. I., & Wyss‐Coray, T. (2014). Microglial dysfunction in brain aging and Alzheimer’s disease. Biochemical Pharmacology, 88, 594–604. 24445162 10.1016/j.bcp.2014.01.008 PMC3972294

[ejn14711-bib-0110] Mouzon, B. C., Bachmeier, C., Ferro, A., Ojo, J. O., Crynen, G., Acker, C. M., … Crawford, F. (2014). Chronic neuropathological and neurobehavioral changes in a repetitive mild traumatic brain injury model. Annals of Neurology, 75, 241–254. 24243523 10.1002/ana.24064

[ejn14711-bib-0111] Mouzon, B. C., Bachmeier, C., Ojo, J. O., Acker, C. M., Ferguson, S., Paris, D., … Crawford, F. (2017). Lifelong behavioral and neuropathological consequences of repetitive mild traumatic brain injury. Annals of Clinical and Translational Neurology, 5, 64–80. 29376093 10.1002/acn3.510 PMC5771321

[ejn14711-bib-0112] Mouzon, B., Chaytow, H., Crynen, G., Bachmeier, C., Stewart, J., Mullan, M., … Crawford, F. (2012). Repetitive mild traumatic brain injury in a mouse model produces learning and memory deficits accompanied by histological changes. Journal of Neurotrauma, 29, 2761–2773. 22900595 10.1089/neu.2012.2498 PMC13588329

[ejn14711-bib-0113] Namjoshi, D. R., Good, C., Cheng, W. H., Panenka, W., Richards, D., Cripton, P. A., & Wellington, C. L. (2013). Towards clinical management of traumatic brain injury: A review of models and mechanisms from a biomechanical perspective. Disease Models & Mechanisms, 6, 1325–1338. 24046354 10.1242/dmm.011320 PMC3820257

[ejn14711-bib-0114] Nelson, A. R., Sweeney, M. D., Sagare, A. P., & Zlokovic, B. V. (2016). Neurovascular dysfunction and neurodegeneration in dementia and Alzheimer's disease. Biochimica et Biophysica Acta, 186, 887–900. 10.1016/j.bbadis.2015.12.016 PMC4821735 26705676

[ejn14711-bib-0115] Newsome, M. R., Durgerian, S., Mourany, L., Scheibel, R. S., Lowe, M. J., Beall, E. B., … Rao, S. M. (2015). Disruption of caudate working memory activation in chronic blast‐related traumatic brain injury. NeuroImage Clinical, 8, 543–553. 26110112 10.1016/j.nicl.2015.04.024 PMC4477106

[ejn14711-bib-0116] Niraula, A., Sheridan, J. F., & Godbout, J. P. (2017). Microglia priming with aging and stress. Neuropsychopharmacology, 42, 318–333. 27604565 10.1038/npp.2016.185 PMC5143497

[ejn14711-bib-0117] Norden, D. M., & Godbout, J. P. (2013). Review: Microglia of the aged brain: Primed to be activated and resistant to regulation. Neuropathology and Applied Neurobiology, 39, 19–34. 23039106 10.1111/j.1365-2990.2012.01306.x PMC3553257

[ejn14711-bib-0118] Norden, D. M., Muccigrosso, M. M., & Godbout, J. P. (2015). Microglial priming and enhanced reactivity to secondary insult in aging, and traumatic CNS injury, and neurodegenerative disease. Neuropharmacology, 96(Pt A), 29–41. 25445485 10.1016/j.neuropharm.2014.10.028 PMC4430467

[ejn14711-bib-0119] Ojo, J. O., Mouzon, B., Algamal, M., Leary, P., Lynch, C., Abdullah, L., … Crawford, F. (2016). Chronic repetitive mild traumatic brain injury results in reduced cerebral blood flow, axonal injury, gliosis, and increased T‐tau and tau oligomers. Journal of Neuropathology and Experimental Neurology, 75, 636–655. 27251042 10.1093/jnen/nlw035 PMC4913432

[ejn14711-bib-0120] Ojo, J. O., Mouzon, B., & Crawford, F. (2016). Repetitive head trauma, chronic traumatic encephalopathy and tau: Challenges in translating from mice to men. Experimental Neurology, 275, 389–404. 26054886 10.1016/j.expneurol.2015.06.003

[ejn14711-bib-0121] Ojo, J. O., Mouzon, B., Greenberg, M. B., Bachmeier, C., Mullan, M., & Crawford, F. (2013). Repetitive mild traumatic brain injury augments tau pathology and glial activation in aged hTau mice. Journal of Neuropathology and Experimental Neurology, 72, 137–151. 23334597 10.1097/NEN.0b013e3182814cdf

[ejn14711-bib-0122] Ojo, J. O., Rezaie, P., Gabbott, P. L., & Stewart, M. G. (2015). Impact of age‐related neuroglial cell responses on hippocampal deterioration. Frontiers in Aging Neuroscience, 7, 57. 25972808 10.3389/fnagi.2015.00057 PMC4413780

[ejn14711-bib-0123] Park, S., Sorenson, C. M., & Shelbani, N. (2015). PECAM‐1 isoforms, eNOS and endoglin axis in regulation of angiogenesis. Clinical Science, 129, 217–234. 25976664 10.1042/CS20140714 PMC4716661

[ejn14711-bib-0124] Paterno, R., Metheny, H., & Cohen, A. S. (2018). Memory deficit in an object location task after mild traumatic brain injury is associated with impaired early object exploration and both are restored by branched chain amino acid dietary therapy. Journal of Neurotrauma, 35, 2117–2124. 29774776 10.1089/neu.2017.5170 PMC6098408

[ejn14711-bib-0125] Petraglia, A. L., Plog, B. A., Dayawansa, S., Chen, M., Dashnaw, M. L., Czerniecka, K., … Huang, J. H. (2014). The spectrum of neurobehavioral sequelae after repetitive mild traumatic brain injury: A novel mouse model of chronic traumatic encephalopathy. Journal of Neurotrauma, 31, 1211–1224. 24766454 10.1089/neu.2013.3255 PMC4082360

[ejn14711-bib-0126] Petraglia, A. L., Plog, B. A., Dayawansa, S., Dashnaw, M. L., Czerniecka, K., Walker, C. T., … Nedergaard, M. (2014). The pathophysiology underlying repetitive mild traumatic brain injury in a novel mouse model of chronic traumatic encephalopathy. Surgical Neurology International, 5, 184. 25593768 10.4103/2152-7806.147566 PMC4287910

[ejn14711-bib-0127] Pluvinage, J. V., Haneym, M. S., Smith, B. A. H., Sun, J., Iram, T., Bonanno, L., … Wyss‐Coray, T. (2019). CD22 blockade restores homeostatic microglial phagocytosis in ageing brains. Nature, 568, 187–192. 30944478 10.1038/s41586-019-1088-4 PMC6574119

[ejn14711-bib-0128] Pop, V., & Badaut, J. (2011). A neurovascular perspective for long‐term changes after brain trauma. Translational Stroke Research, 2, 533–545. 22350620 10.1007/s12975-011-0126-9 PMC3281750

[ejn14711-bib-0129] Prins, M. L., Alexander, D., Giza, C. C., & Hovda, D. A. (2013). Repeated mild traumatic brain injury: Mechanisms of cerebral vulnerability. Journal of Neurotrauma, 30, 30–38. 23025820 10.1089/neu.2012.2399 PMC4047842

[ejn14711-bib-0130] Prins, M. L., Hales, A., Reger, M., Giza, C. C., & Hovda, D. A. (2010). Repeat traumatic brain injury in the juvenile rat is associated with increased axonal injury and cognitive impairments. Developmental Neuroscience, 32, 510–518. 20829578 10.1159/000316800 PMC3215244

[ejn14711-bib-0131] Privratsky, J. R., & Newman, P. J. (2014). PECAM‐1: Regulator of endothelial junctional integrity. Cell and Tissue Research, 355, 607–619. 24435645 10.1007/s00441-013-1779-3 PMC3975704

[ejn14711-bib-0132] Raj, D. D., Jaarsma, D., Holtman, I. R., Olah, M., Ferreira, F. M., Schaafsma, W., … Boddeke, H. W. (2014). Priming of microglia in a DNA‐repair deficient model of accelerated aging. Neurobiology of Aging, 35, 2147–2160. 24799273 10.1016/j.neurobiolaging.2014.03.025

[ejn14711-bib-0133] Redell, J. B., & Dash, P. K. (2007). Traumatic brain injury stimulates hippocampal catechol‐o‐methyl transferase expression in microglia. Neuroscience Letters, 413, 36–41. 17240060 10.1016/j.neulet.2006.11.060 PMC1857315

[ejn14711-bib-0134] Reiner, A., Heldt, S. A., Presley, C. S., Guley, N. H., Elberger, A. J., Deng, Y., … Moore, B. M. (2015). Motor, visual and emotional deficits in mice after closed‐head mild TBI are alleviated by the novel CB2 inverse agonist SMM‐189. International Journal of Molecular Sciences, 16, 758–787. 10.3390/ijms16010758 PMC4307274 25561230

[ejn14711-bib-0135] Rodriguez‐Grande, B., Ichkova, A., Lemarchant, S., & Badaut, J. (2017). Early to long‐term alterations of CNS barriers after traumatic brain injury: Considerations for drug development. American Association of Pharmaceutical Scientists Journal, 19(6), 1615–1625. 28905273 10.1208/s12248-017-0123-3

[ejn14711-bib-0136] Roy, S., Ha, J., Trudeau, K., & Beglova, E. (2010). Vascular basement membrane thickening in diabetic retinopathy. Current Eye Research, 35, 1045–1056. 20929292 10.3109/02713683.2010.514659

[ejn14711-bib-0137] Safaiyan, S., Kannaiyan, N., Snaidero, N., Brioschi, S., Biber, K., Yona, S., … Simons, M. (2016). Age‐related myelin degradation burdens the clearance function of microglia during aging. Nature Neuroscience, 19, 995–998. 27294511 10.1038/nn.4325 PMC7116794

[ejn14711-bib-0138] Sandsmark, D. K., Bashir, A., Wellington, C. L., & Diaz‐Arrastia, R. (2019). Cerebral microvascular injury: A potentially treatable endophenotype of traumatic brain injury‐induced neurodegeneration. Neuron, 103(3), 367–379. 31394062 10.1016/j.neuron.2019.06.002 PMC6688649

[ejn14711-bib-0139] Selwyn, R. G., Cooney, S. J., Khayrullina, G., Hockenbury, N., Wilson, C. M., Jaiswal, S., … Byrnes, K. R. (2016). Outcome after repetitive mild traumatic brain injury is temporally related to glucose uptake profile at time of second injury. Journal of Neurotrauma, 33, 1479–1491. 26650903 10.1089/neu.2015.4129

[ejn14711-bib-0140] Shipton, O. A., El‐Gaby, M., Apergis‐Schoute, J., Deisseroth, K., Bannerman, D. M., Paulsen, O., & Kohl, M. M. (2014). Left–right dissociation of hippocampal memory processes in mice. Proceedings of the National Academy of Sciences of the United States of America, 111, 15238–15243. 25246561 10.1073/pnas.1405648111 PMC4210314

[ejn14711-bib-0141] Sierra, A., Beccari, S., Diaz‐Aparicio, I., Encinas, J. M., Comeau, S., & Tremblay, M. È. (2014). Surveillance, phagocytosis, and inflammation: How never‐resting microglia influence adult hippocampal neurogenesis. Neural Plasticity, 2014, 610343. 24772353 10.1155/2014/610343 PMC3977558

[ejn14711-bib-0142] Smith, D. H., Hicks, R., & Povlishock, J. T. (2013). Therapy development for diffuse axonal injury. Journal of Neurotrauma, 30, 307–323. 23252624 10.1089/neu.2012.2825 PMC3627407

[ejn14711-bib-0143] Smith, J. A., Das, A., Ray, S. K., & Banik, N. L. (2012). Role of pro‐inflammatory cytokines released from microglia in neurodegenerative diseases. Brain Research Bulletin, 87, 10–20. 22024597 10.1016/j.brainresbull.2011.10.004 PMC9827422

[ejn14711-bib-0144] Spittau, B. (2017). Aging microglia ‐ phenotypes, functions and implications for age‐related neurodegenerative diseases. Frontiers in Aging Neuroscience, 9, 194. 28659790 10.3389/fnagi.2017.00194 PMC5469878

[ejn14711-bib-0145] Stanimirovic, D. B., & Friedman, A. (2012). Pathophysiology of the neurovascular unit: Disease cause or consequence? Journal of Cerebral Blood Flow and Metabolism, 32, 1207–1221. 22395208 10.1038/jcbfm.2012.25 PMC3390807

[ejn14711-bib-0146] Stojiljkovic, M. R., Ain, Q., Bondeva, T., Heller, R., Schmeer, C., & Witte, O. W. (2019). Phenotypic and functional differences between senescent and aged murine microglia. Neurobiology of Aging, 74, 56–69. 30439594 10.1016/j.neurobiolaging.2018.10.007

[ejn14711-bib-0147] Streit, W. J., Braak, H., Xue, Q. S., & Bechmann, I. (2009). Dystrophic (senescent) rather than activated microglial cells are associated with tau pathology and likely precede neurodegeneration in Alzheimer’s disease. Acta Neuropathologica, 118, 475–485. 19513731 10.1007/s00401-009-0556-6 PMC2737117

[ejn14711-bib-0148] Streit, W. J., Xue, Q. S., Tischer, J., & Bechmann, I. (2014). Microglial pathology. Acta Neuropathologica Communications, 2, 142. 25257319 10.1186/s40478-014-0142-6 PMC4180960

[ejn14711-bib-0149] Tagge, C. A., Fisher, A. M., Minaeva, O. V., Gaudreau‐Balderrama, A., Moncaster, J. A., Zhang, X. L., … Goldstein, L. E. (2018). Concussion, microvascular injury, and early tauopathy in young athletes after impact head injury and an impact concussion mouse model. Brain, 141(2), 422–458. 29360998 10.1093/brain/awx350 PMC5837414

[ejn14711-bib-0150] Tan, X. L., Xue, Y. Q., Ma, T., Wang, X., Li, J. J., Lan, L., … Liao, F. F. (2015). Partial eNOS deficiency causes spontaneous thrombotic cerebral infarction, amyloid angiopathy and cognitive impairment. Molecular Neurodegeneration, 10, 24. 26104027 10.1186/s13024-015-0020-0 PMC4479241

[ejn14711-bib-0151] Tay, T. L., Savage, J. C., Hui, C. W., Bisht, K., & Tremblay, M. È. (2017). Microglia across the lifespan: From origin to function in brain development, plasticity and cognition. Physiol., 595, 1929–1945. 10.1113/JP272134 PMC5350449 27104646

[ejn14711-bib-0152] Taylor, P. A., Ludwigsen, J. S., & Ford, C. C. (2014). Investigation of blast‐induced traumatic brain injury. Brain Injury, 28, 879–895. 24766453 10.3109/02699052.2014.888478 PMC4046872

[ejn14711-bib-0153] Thomsen, M. S., Routhe, L. J., & Moos, T. (2017). The vascular basement membrane in the healthy and pathological brain. Journal of Cerebral Blood Flow and Metabolism, 37, 3300–3317. 28753105 10.1177/0271678X17722436 PMC5624399

[ejn14711-bib-0154] Tischer, J., Krueger, M., Mueller, W., Staszewski, O., Prinz, M., Streit, W. J., & Bechmann, I. (2016) Inhomogeneous distribution of Iba‐1 characterizes microglial pathology in Alzheimer's disease. Glia, 64(9), 1562‐1572. 27404378 10.1002/glia.23024

[ejn14711-bib-0155] Torres‐Platas, S. G., Comeau, S., Rachalski, A., Bo, G. D., Cruceanu, C., Turecki, G., … Mechawar, N. (2014). Morphometric characterization of microglial phenotypes in human cerebral cortex. Journal of Neuroinflammation, 11, 12. 24447857 10.1186/1742-2094-11-12 PMC3906907

[ejn14711-bib-0156] Turner, R. C., Lucke‐Wold, B. P., Logsdon, A. F., Robson, M. J., Dashnaw, M. L., Huang, J. H., … Petraglia, A. L. (2015). The quest to model chronic traumatic encephalopathy: a multiple model and injury paradigm experience. Frontiers in Neurology, 6, 222. 26539159 10.3389/fneur.2015.00222 PMC4611965

[ejn14711-bib-0157] Uryu, K., Laurer, H., McIntosh, T., Pratico, D., Martinez, D., Leight, S., … Trojanowski, J. Q. (2002). Repetitive mild brain trauma accelerates Abeta deposition, lipid peroxidation, and cognitive impairment in a transgenic mouse model of Alzheimer amyloidosis. Journal of Neuroscience, 22, 446–454. 11784789 10.1523/JNEUROSCI.22-02-00446.2002 PMC6758680

[ejn14711-bib-0158] Vagnozzi, R., Signoretti, S., Tavazzi, B., Floris, R., Ludovici, A., Marziali, S., … Lazzarino, G. (2007). Temporal window of metabolic brain vulnerability to concussions: Mitochondrial‐related impairment ‐ Part I. Neurosurgery, 61, 379–389. 10.1227/01.NEU.0000280002.41696.D8 17762751

[ejn14711-bib-0159] Van Buuren, S., & Groothuis‐Oudshoorn, K. (2011). MICE: Multivariate imputation by chained equations in R. Journal of Statistical Software, 45, 1–67.

[ejn14711-bib-0160] Verdonk, F., Roux, P., Flamant, P., Fiette, L., Bozza, F. A., Simard, S., … Danckaert, A. (2016). Phenotypic clustering: A novel method for microglial morphology analysis. Journal of Neuroinflammation, 13, 153. 27317566 10.1186/s12974-016-0614-7 PMC4912769

[ejn14711-bib-0161] Walker, F. R., Beynon, S. B., Jones, K. A., Zhao, Z., Kongsui, R., Cairns, M., & Nilsson, M. (2014). Dynamic structural remodelling of microglia in health and disease: A review of the models, the signals and the mechanisms. Brain, Behavior, and Immunity, 37, 1–14. 10.1016/j.bbi.2013.12.010 24412599

[ejn14711-bib-0162] Walker, K. R., & Tesco, G. (2013). Molecular mechanisms of cognitive dysfunction following traumatic brain injury. Front. Aging Neuroscience, 5, 29. 10.3389/fnagi.2013.00029 PMC3705200 23847533

[ejn14711-bib-0163] Wang, G., Zhang, J., Hu, X., Zhang, L., Mao, L., Jiang, X., … Chen, J. (2013). Microglia/macrophage polarization dynamics in white matter after traumatic brain injury. Journal of Cerebral Blood Flow & Metabolism, 33, 1864–1874. 23942366 10.1038/jcbfm.2013.146 PMC3851898

[ejn14711-bib-0164] Wang, Y., Wei, Y., Oguntayo, S., Wilkins, W., Arun, P., Valiyaveettil, M., … Nambiar, M. P. (2011). Tightly coupled repetitive blast‐induced traumatic brain injury: Development and characterization in mice. Journal of Neurotrauma, 28, 2171–2183. 21770761 10.1089/neu.2011.1990

[ejn14711-bib-0165] Washington, P. M., Villapol, S., & Burns, M. P. (2016). Polypathology and dementia after brain trauma: Does brain injury trigger distinct neurodegenerative diseases, or should they be classified together as traumatic encephalopathy? Experimental Neurology, 275, 381–388. 26091850 10.1016/j.expneurol.2015.06.015 PMC4681695

[ejn14711-bib-0166] Watanabe, J., Shetty, A. K., Hattiangady, B., Kim, D. K., Foraker, J. E., Nishida, H., & Prockop, D. J. (2013). Administration of TSG‐6 improves memory after traumatic brain injury in mice. Neurobiology of Diseases, 59, 86–99. 10.1016/j.nbd.2013.06.017 PMC3790317 23851308

[ejn14711-bib-0167] Weil, Z. M., Gaier, K. R., & Karelina, K. (2014). Injury timing alters metabolic, inflammatory and functional outcomes following repeated mild traumatic brain injury. Neurobiology of Diseases, 70, 108–116. 10.1016/j.nbd.2014.06.016 24983210

[ejn14711-bib-0168] Weiner, M. W., Veitch, D. P., Hayes, J., Neylan, T., Grafman, J., Aisen, P. S., … Department of Defense Alzheimer's Disease Neuroimaging Initiative (2014). Effects of TBI and posttraumatic stress disorder on Alzheimer's disease in veterans. Alzheimer Dementia, 10, S226–S235. 10.1016/j.jalz.2014.04.005 PMC4392759 24924673

[ejn14711-bib-0169] Williams, R. M., Puetz, T. W., Giza, C. C., & Broglio, S. P. (2015). Concussion recovery time among high school and collegiate athletes: A systematic review and meta‐analysis. Sports Medicine (Auckland, N. Z.), 45, 893–903. 10.1007/s40279-015-0325-8 PMC4441834 25820456

[ejn14711-bib-0170] Winston, C. N., Noël, A., Neustadtl, A., Parsadanian, M., Barton, D. J., Chellappa, D., … Burns, M. P. (2016). Dendritic spine loss and chronic white matter inflammation in a mouse model of highly repetitive head trauma. American Journal of Pathology, 186, 552–567. 10.1016/j.ajpath.2015.11.006 PMC4816714 26857506

[ejn14711-bib-0171] Witcher, K. G., Eiferman, D. S., & Godbout, J. P. (2015). Priming the inflammatory pump of the CNS after traumatic brain injury. Trends in Neurosciences, 38, 609–620. 26442695 10.1016/j.tins.2015.08.002 PMC4617563

[ejn14711-bib-0172] Woodfin, A., Voisin, M. B., & Nourshargh, S. (2007). PECAM‐1: A multi‐functional molecule in inflammation and vascular biology. Arteriosclerosis, Thrombosis, and Vascular Biology, 27, 2514–2523. 10.1161/ATVBAHA.107.151456 17872453

[ejn14711-bib-0173] Wright, R. M., Post, A., Hoshizaki, B., & Ramesh, K. T. (2013). A multiscale computational approach to estimating axonal damage under inertial loading of the head. Journal of Neurotrauma, 30(2), 102–118. 22992118 10.1089/neu.2012.2418

[ejn14711-bib-0174] Xu, L., Nguyen, J. V., Lehar, M., Menon, A., Rha, E., Arena, J., … Koliatsos, V. E. (2016). Repetitive mild traumatic brain injury with impact acceleration in the mouse: Multifocal axonopathy, neuroinflammation, and neurodegeneration in the visual system. Experimental Neurology, 275, 436–449. 25450468 10.1016/j.expneurol.2014.11.004

[ejn14711-bib-0175] Yamada, J., & Jinno, S. (2013). Novel objective classification of reactive microglia following hypoglossal axotomy using hierarchical cluster analysis. The Journal of Comparative Neurology, 521, 1184–1201. 22987820 10.1002/cne.23228

[ejn14711-bib-0176] Yang, Z., Wang, P., Morgan, D., Bruijnzeel, A. W., Lin, D., Pan, J., … Wang, K. K. W. (2015). Temporal MRI characterization, neurobiochemical and neurobehavioral changes in a mouse repetitive concussive head injury model. Scientific Reports, 5, 11178. 26058556 10.1038/srep11178 PMC4461921

[ejn14711-bib-0177] Zakaria, N., Kallakuri, S., Bandaru, S., & Cavanaugh, J. M. (2012). Temporal assessment of traumatic axonal injury in the rat corpus callosum and optic chiasm. Brain Research, 1467, 81–90. 22652307 10.1016/j.brainres.2012.05.046

[ejn14711-bib-0178] Zhao, Z., Nelson, A. R., Betsholtz, C., & Zlokovic, B. V. (2015). Establishment and dysfunction of the blood‐brain barrier. Cell, 163, 1064–1078. 26590417 10.1016/j.cell.2015.10.067 PMC4655822

[ejn14711-bib-0179] Ziebell, J. M., Rowe, R. K., Muccigrosso, M. M., Reddaway, J. T., Adelson, P. D., Godbout, J. P., & Lifshitz, J. (2017). Aging with a traumatic brain injury: Could behavioral morbidities and endocrine symptoms be influenced by microglial priming? Brain Behavior & Immunity, 59, 1–7. 10.1016/j.bbi.2016.03.008 26975888

